# Correction: Nash equilibrium of attack and defense behaviors between predators and prey

**DOI:** 10.1371/journal.pcbi.1013969

**Published:** 2026-02-13

**Authors:** The PLOS Computational Biology Staff

## Notice of Republication

Incorrect versions of S2 Data, S3 Data, S4 Data, S5 Data, S6 Data, S8 Data and S9 Data were published in error. This article was republished on January 28, 2026, to replace these with the correct files.

The publisher apologizes for the error. Please download this article again to view the correct version. The originally published, uncorrected article and the republished, corrected articles are provided here for reference.

## Supporting information

S1 File Originally published, uncorrected article. (PDF)

S2 File Republished, corrected article. (PDF)
