# Supplementary figures and images for: Nash equilibrium of attack and defense behaviors between predators and prey
Source: PLoS Comput Biol. 2025 Nov 21;21(11):e1013730. doi: 10.1371/journal.pcbi.1013730 (PMC12671891; doi:10.1371/journal.pcbi.1013730)

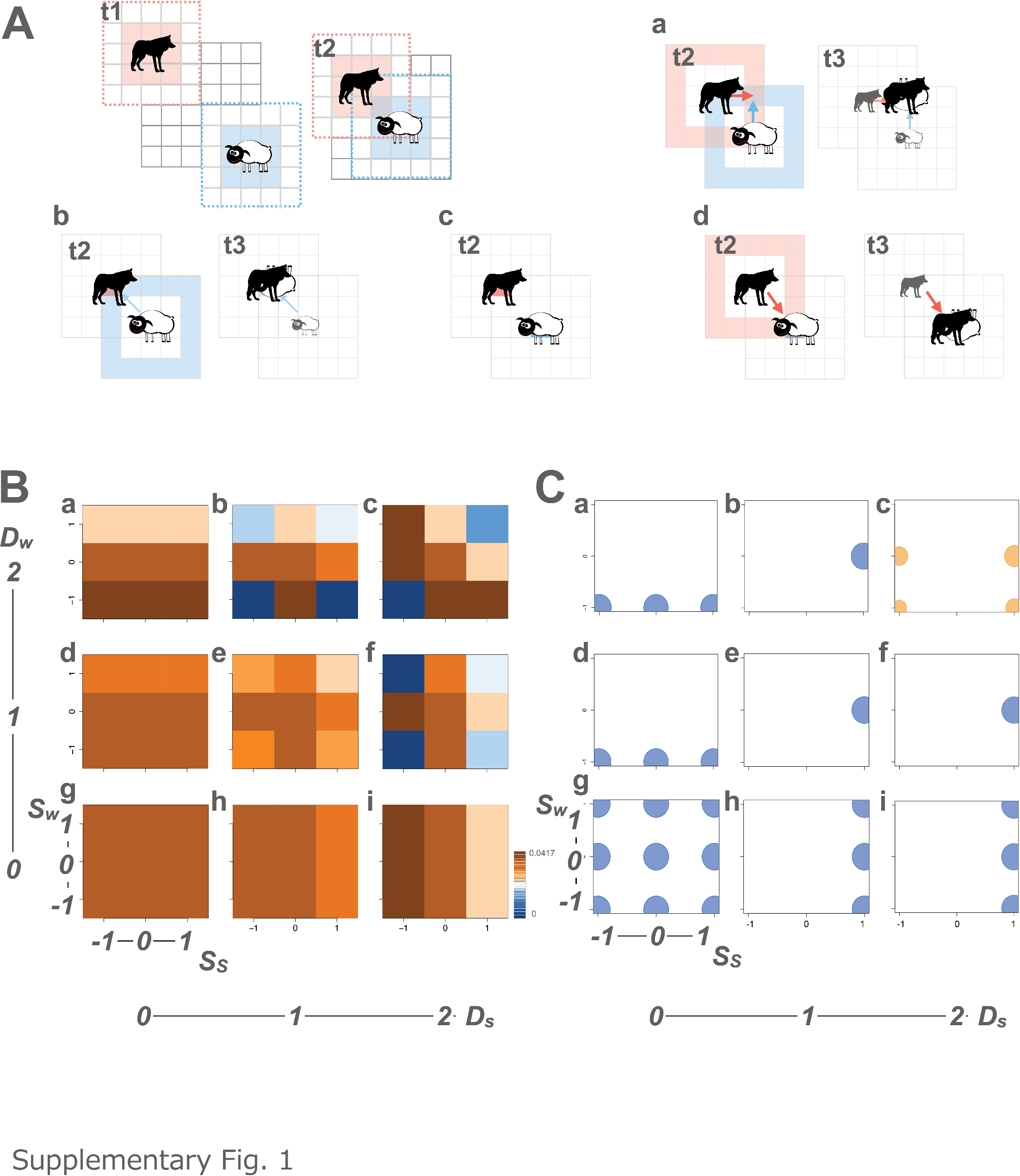

Supplement: S1 Fig — A illustrates the attack and defense behaviors between a single predator and a single prey (agents) in a grid world. Three time steps are shown (t1, t2, t3). The dotted lines represent their detection ranges (Dw = 2 for the predator [wolf] and Ds = 2 for the prey [sheep]). At t1, neither agent detects the opponent and each moves one grid per time step. The shaded grids indicate the possible positions they may occupy at the next time step. At t2, if they detect each other, both adjust their speeds by Si (i = w for wolf and s for sheep), changing their speed to 1 + Si. Aa shows a case where Sw = Ss = 1. The wolf chases the sheep (1 + Sw = 2), and the sheep escapes (1 + Ss = 2). If both occupy the same grid cell, as indicated by the arrows in Aa-t2, the wolf eats the sheep at t3. Ab shows a case where Sw = −1 and Ss = 1. The wolf ambushes (1 + Sw = 0), and the sheep escapes (1 + Ss = 2). If both occupy the same grid cell, as shown by the arrow in Ab-t2, the wolf eats the sheep at t3. Ac shows a case where Sw = −1 and Ss = −1. The wolf ambushes (1 + Sw = 0), and the sheep freezes (1 + Ss = 0). Both remain in place and do not encounter. Ad shows a case where Sw = 1 and Ss = −1. The wolf chases (1 + Sw = 2), and the sheep freezes (1 + Ss = 0). If both occupy the same grid cell, as shown by the arrow in Ad-t2, the wolf eats the sheep at t3. For the illustrations of the wolf and sheep, we used open-source images from Openclipart (https://openclipart.org/detail/254708/wolf-silhouette-2; https://openclipart.org/detail/174830/sheep). B illustrates landscape diagrams that depict encounter probabilities based on combinations of detection distance (Di). For this S1B Fig, we calculated encounter probabilities including combinations in which the agents were adjacent when Di = 2 (S2 File). This program corresponds to calculating encounter probabilities for a two-step interaction. The diagrams show nine combinations, ranging from Ds = 0 and Dw = 2 (a) to Ds = 2 and Dw = 0 (i). In eac [file pcbi.1013730.s001.tif]

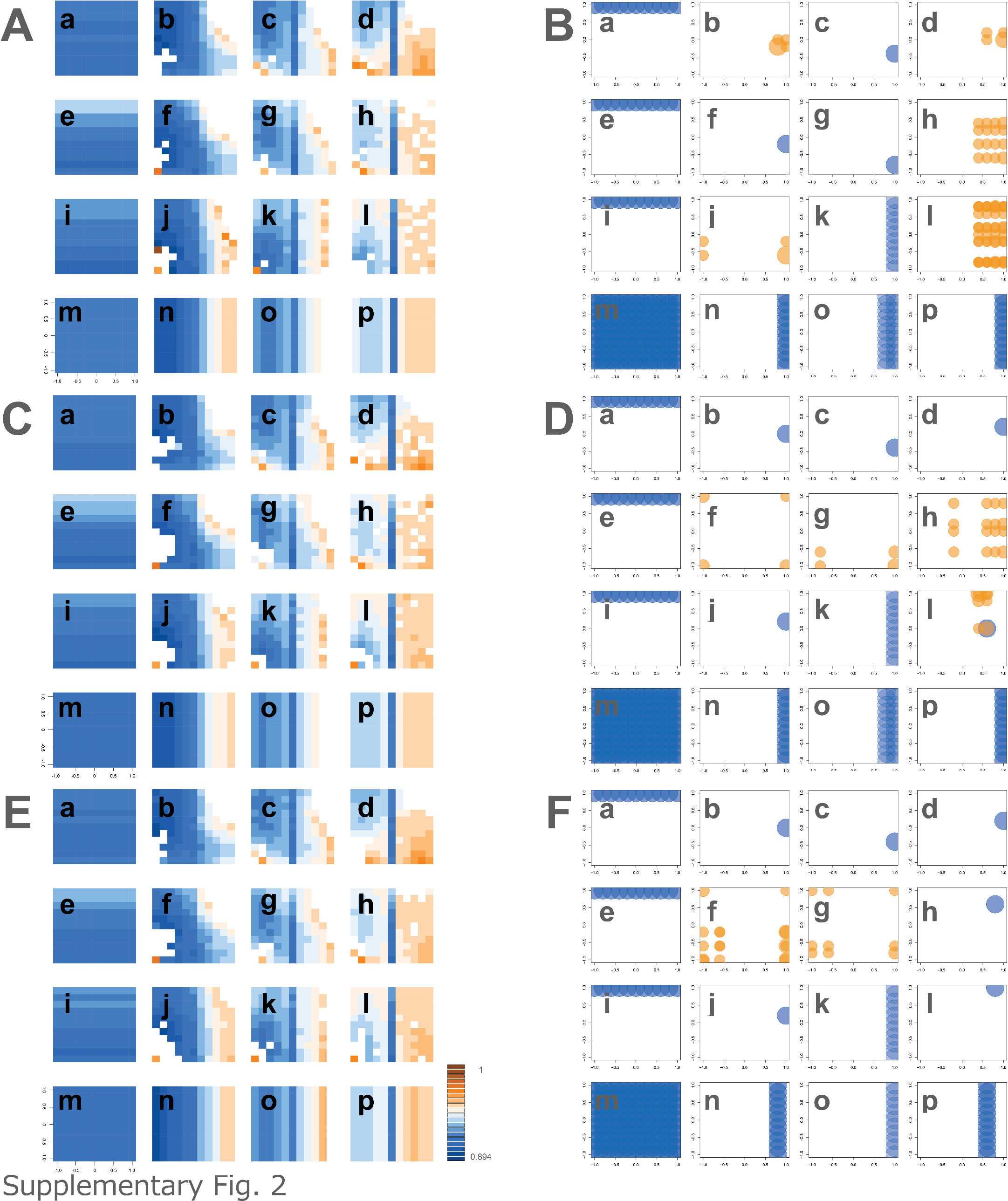

Supplement: S2 Fig — A shows the landscapes of Pdiff under the reproduction coefficient of predators (r = 3.2). Sixteen landscapes depict different combinations of agents’ detection distance, Di (= 0, 1, 2, 3) (a–p). In each landscape, the x-axis represents the prey’s speed change (Ss) and the y-axis represents the predator’s speed change (Sw). Pdiff values are color-coded: darker brown indicates higher payoff differences favoring prey, and darker blue indicates lower payoff differences (or advantage to predators). B depicts Nash equilibrium speed changes in sixteen landscapes under different combinations of agents’ detection distance under r = 3.2 (a–p). Circle positions represent the Nash equilibrium speed changes of prey (S¯s) and predators (S¯w) on the x-axis and y-axis, respectively, at the same coordinates as the Pdiff landscapes. Circle diameters reflect the occurrence probabilities (o―i) of these behaviors. Blue circles indicate uniquely determined behaviors (o―i = 1). Orange circles represent probabilistic behaviors (0 <o―i < 1). C and D show those under r = 3.6. E and F show those under r = 4.0. (TIF) [file pcbi.1013730.s002.tif]

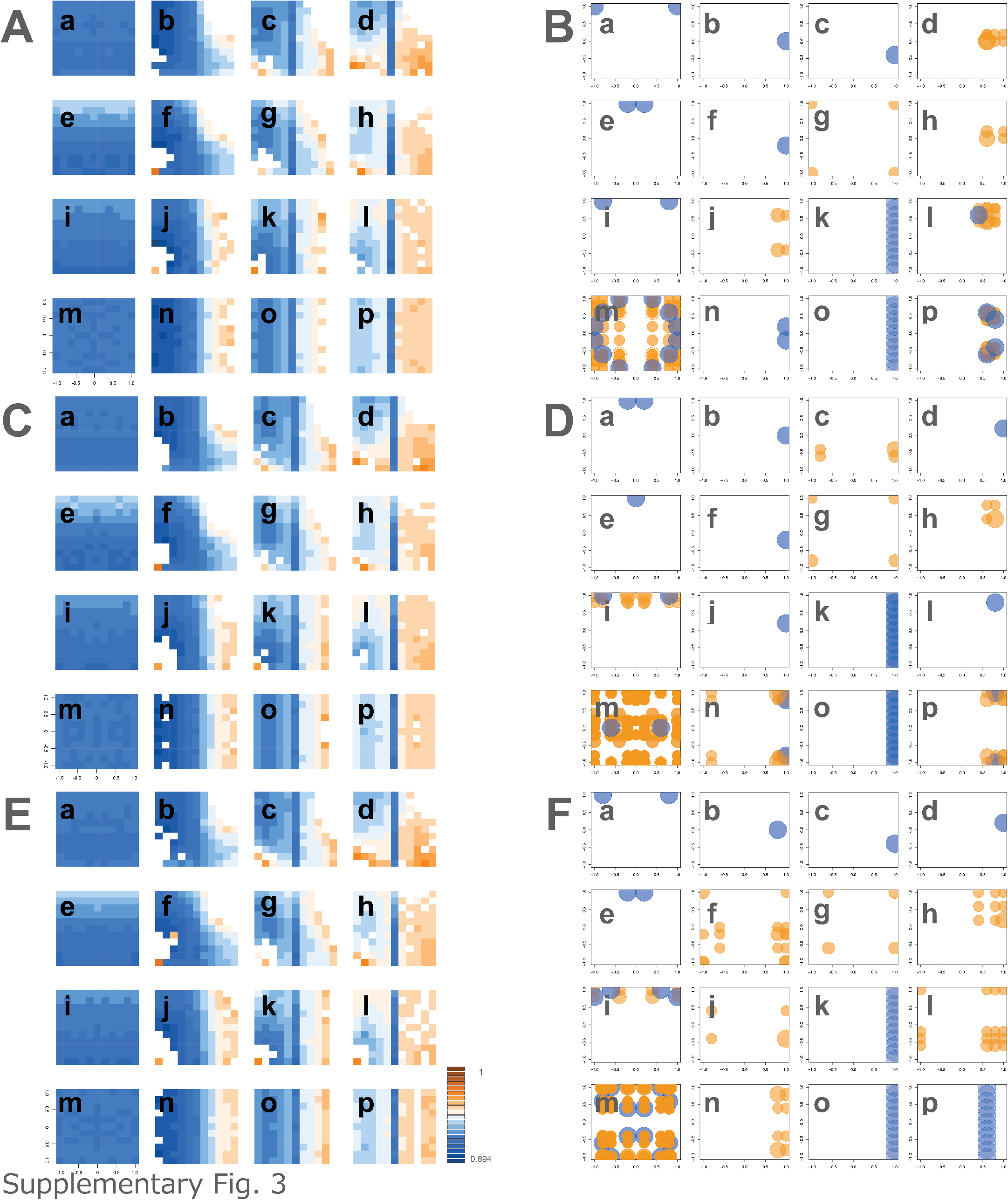

Supplement: S3 Fig — A and B show the landscapes of Pdiff and Nash equilibrium speed changes, respectively, under the reproduction coefficient of predators (r = 3.2). C and D show those under r = 3.6. E and F show those under r = 4.0. (TIF) [file pcbi.1013730.s003.tif]

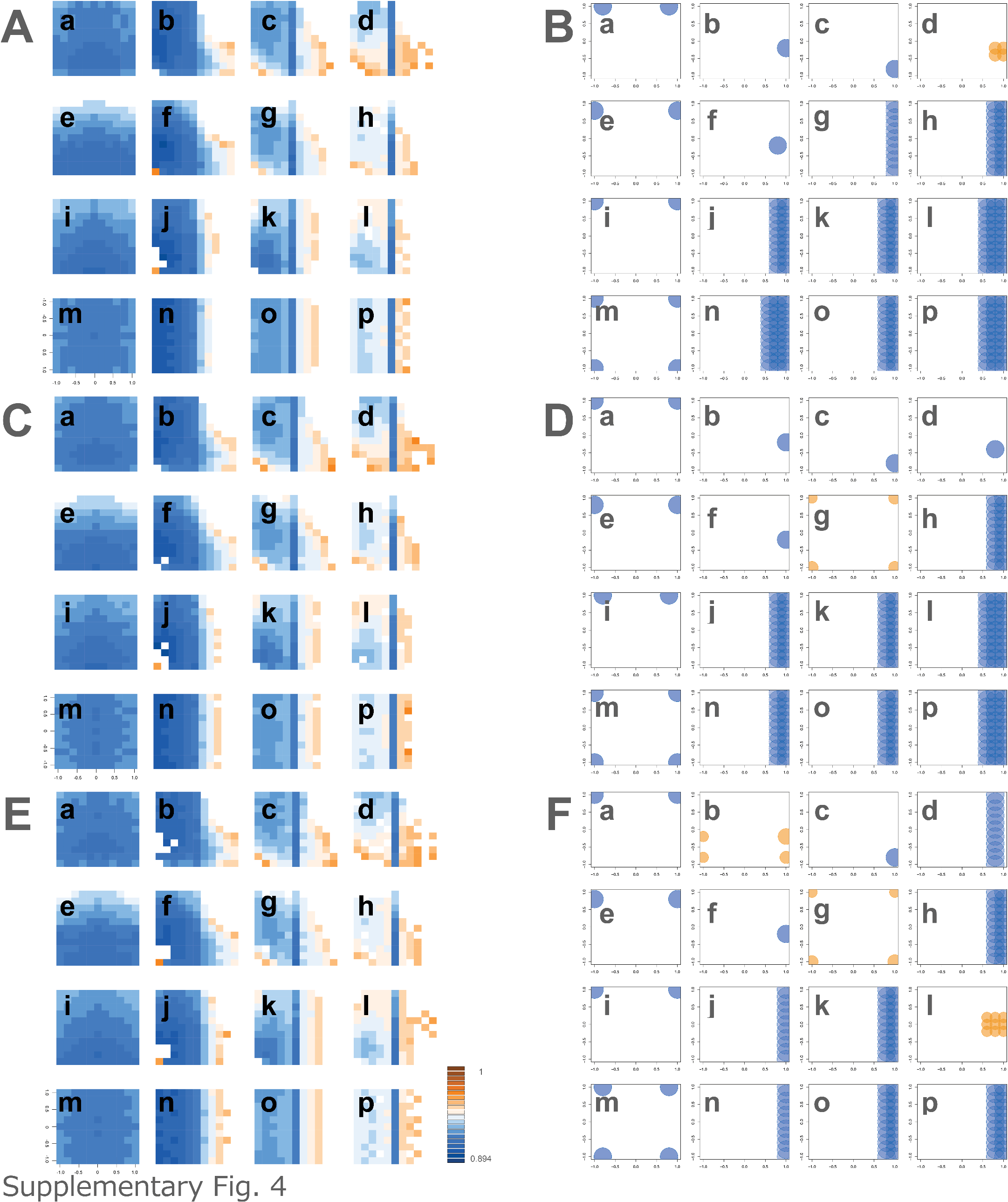

Supplement: S4 Fig — A and B show the landscapes of Pdiff and Nash equilibrium speed changes, respectively, under the reproduction coefficient of predators (r = 3.2). C and D show those under r = 3.6. E and F show those under r = 4.0. (TIF) [file pcbi.1013730.s004.tif]

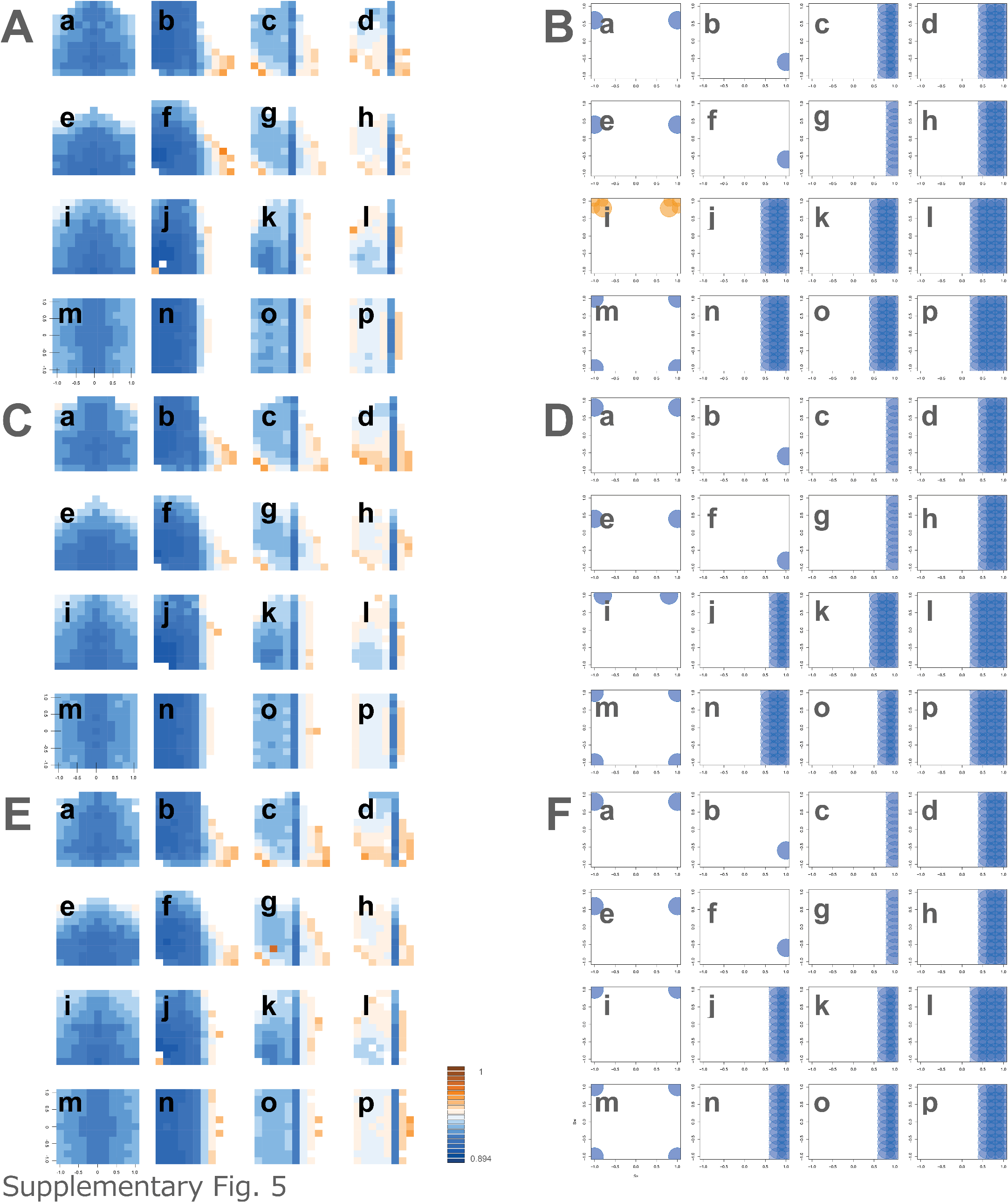

Supplement: S5 Fig — A and B show the landscapes of Pdiff and Nash equilibrium speed changes, respectively, under the reproduction coefficient of predators (r = 3.2). C and D show those under r = 3.6. E and F show those under r = 4.0. (TIF) [file pcbi.1013730.s005.tif]

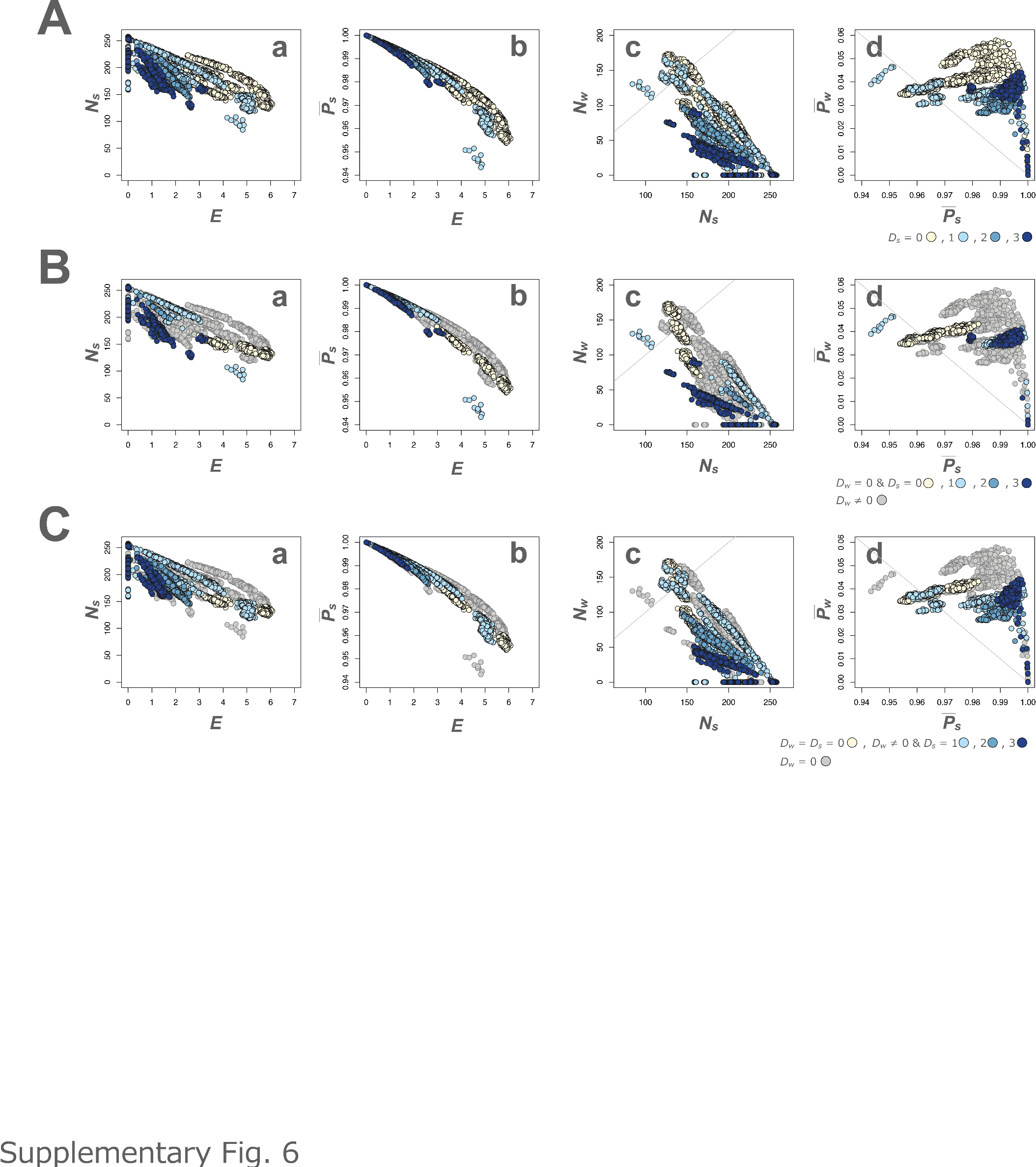

Supplement: S6 Fig — A shows scatter plots of all data across the behavioral cost coefficients (cb) and the reproduction coefficients of predators (r), illustrating the relationships among the encounter frequency (E), number of agents (Ni), and Nash equilibrium payoff (P¯i) (where i = w for wolf and s for sheep), based on actual measurements obtained by assigning Nash equilibrium behaviors. Aa shows a scatter plot of E on the x-axis against Ns on the y-axis, and Ab shows a scatter plot of E on the x-axis against P―s on the y-axis. Ac shows a scatter plot of Ns versus Nw, along the line Ns = Nw. Ad shows a scatter plot of P―s versus P―w, along the line P―s + P―w = 1. Beige, light blue, blue, and dark blue points represent Ds = 0, 1, 2, and 3, respectively, across all values of Dw (0–3). Ba–Bd show the same scatter plots, with Dw = 0 highlighted in the colors and Dw ≠ 0 in gray. Ca–Cd show the same scatter plots, with Dw ≠ 0 highlighted in the colors and Dw = 0 in gray. Beige points represent Ds = Dw = 0 in B and C. Regardless of Dw, increasing Ds reduces E (dark blue points shift to the left side in Aa, Ba, and Ca), increases P―s (dark blue points shift to the upper-left side in Ab, Bb, and Cb, and dark blue points shift to the right side in Ad, Bd, and Cd), and reduces Nw (dark blue points shift to the lower side in Ac, Bc, and Cc). (TIF) [file pcbi.1013730.s006.tif]

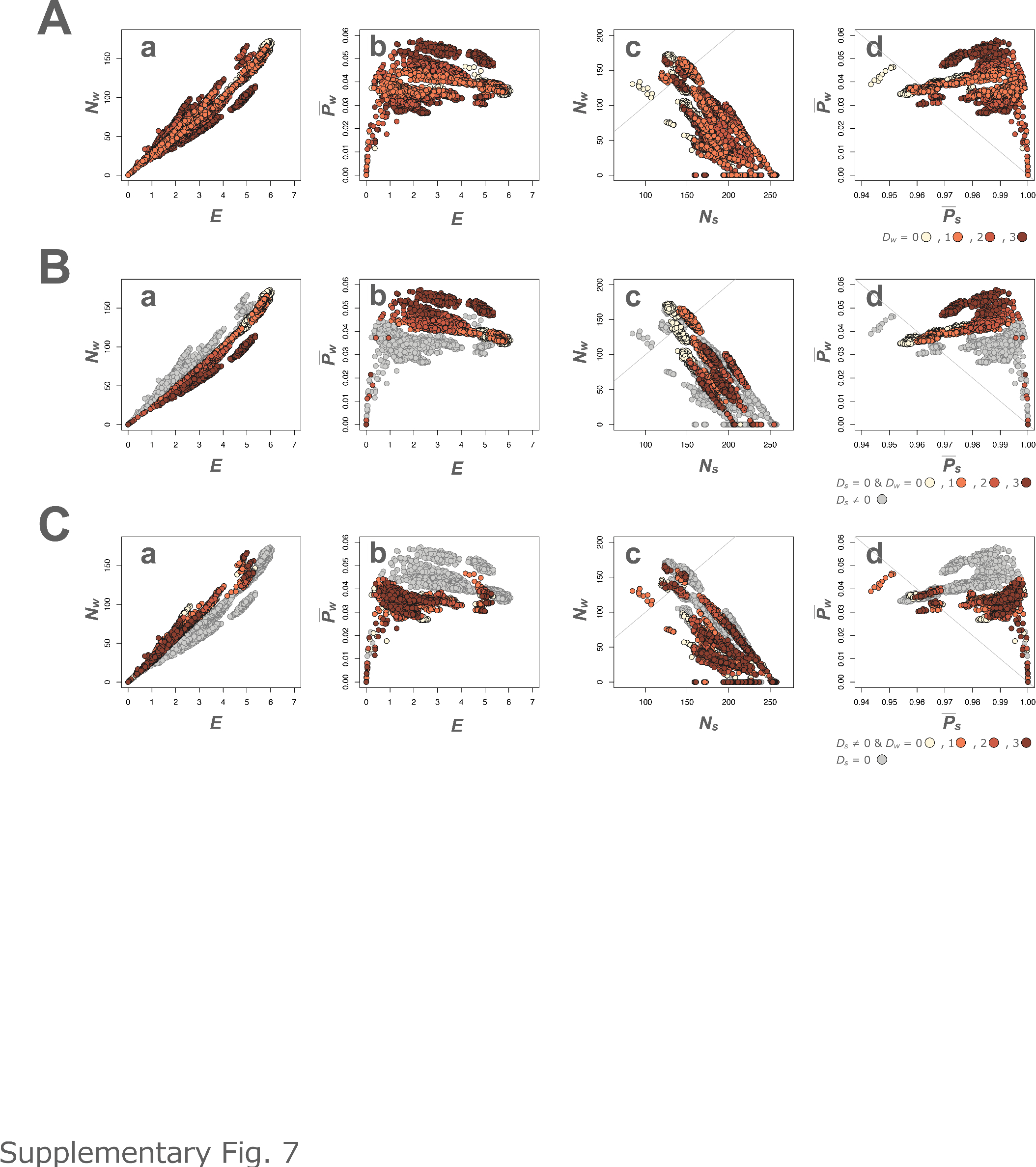

Supplement: S7 Fig — A shows scatter plots of all data across the behavioral cost coefficients (cb) and the reproduction coefficients of predators (r), illustrating the relationships among the encounter frequency (E), number of agents (Ni), and Nash equilibrium payoff (P¯i) (where i = w for wolf and s for sheep), based on actual measurements obtained by assigning Nash equilibrium behaviors. Aa and Ab show scatter plots of E on the x-axis against Nw and P¯w on the y-axis, respectively. Ac shows a scatter plot of Ns versus Nw, along the line Ns = Nw. Ad shows a scatter plot of P―s versus P―w, along the line P―s + P―w = 1. Beige, orange, brown, and dark brown points represent Dw = 0, 1, 2, and 3, respectively, across all values of Ds (Ds = 0–3). Ba–Bd show the same scatter plots, with Ds = 0 highlighted in the colors and Ds ≠ 0 in gray. When Ds = 0, as Dw increases, Nw decreases (dark brown points shift to the lower side in Ba). P―w increases (dark brown points shift to the upper side in Bb). Ns increases and Nw decreases (dark brown points shift to the lower-right side in Bc). P―s and P―w increase (dark brown points shift to the upper-right side in Bd). Ca–Cd show the same scatter plots, with Ds ≠ 0 highlighted in the colors and Ds = 0 in gray. (TIF) [file pcbi.1013730.s007.tif]

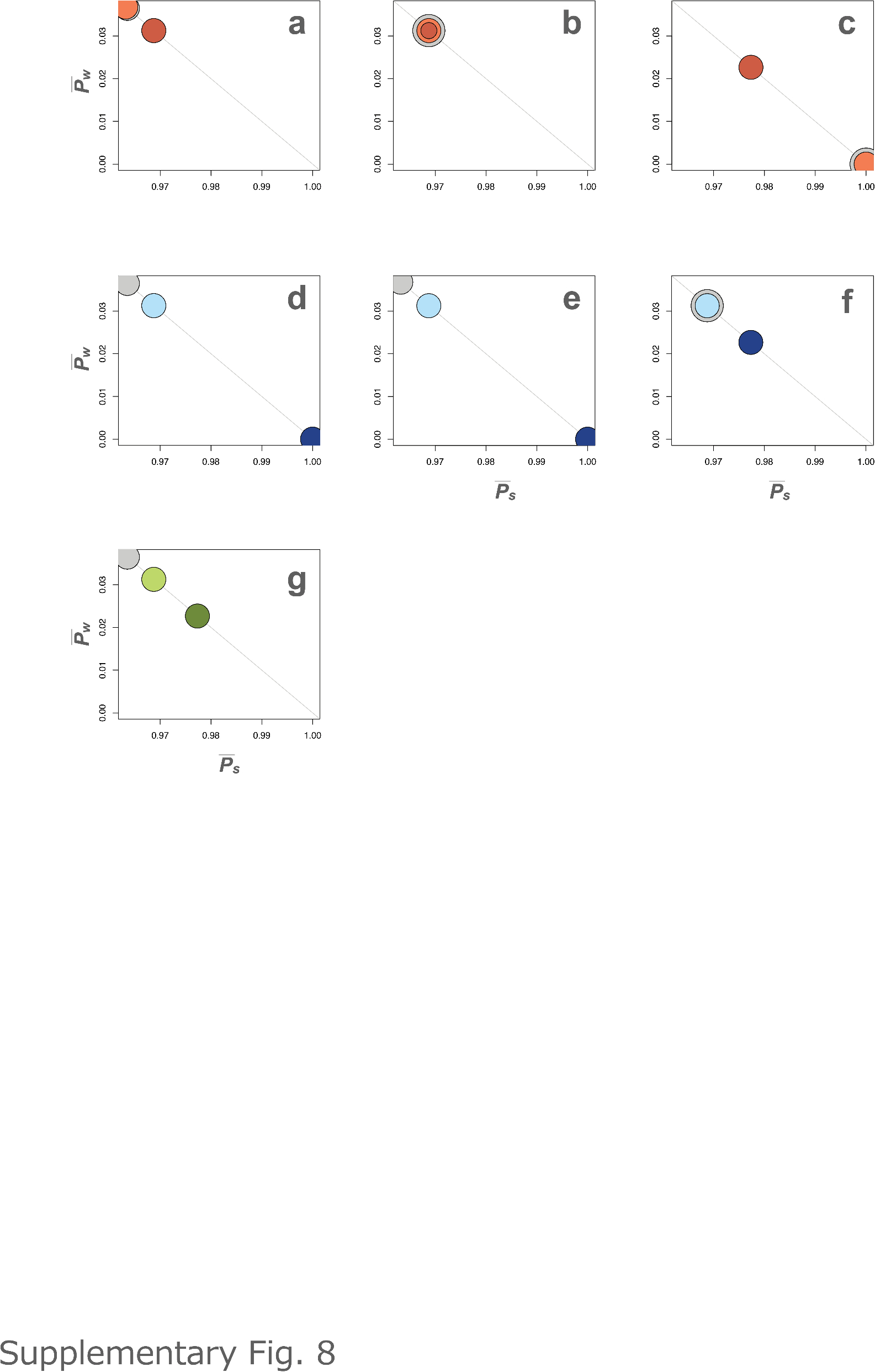

Supplement: S8 Fig — Scatter plots show Nash equilibrium payoffs (P¯i) (where i = w for wolf and s for sheep), with P―s on the x-axis and P―w on the y-axis. a–c show effects of predator’s detection distance (Dw) on the payoffs P―i, while holding the prey’s detection distance (Ds) constant (Ds = 0 in a, 1 in b, and 2 in c). Gray, orange, and brown points represent Dw = 0, 1, and 2, respectively. d–f show the effects of Ds on P―i while holding Dw constant (Dw = 0 in d, 1 in e, and 2 in f). Gray, light blue, and blue points represent Ds = 0, 1, and 2, respectively. g shows the combined effects of Dw and Ds on P―i. Gray, light green, and dark green points represent Dw = Ds = 0, Dw = Ds = 1, and Dw = Ds = 2, respectively. All plots are on the diagonal line (P―s + P―w = 1), demonstrating that the games between a single predator and prey are constant-sum. In b, c, and f, the point sizes are modified to make their overlaps visible. (TIF) [file pcbi.1013730.s008.tif]

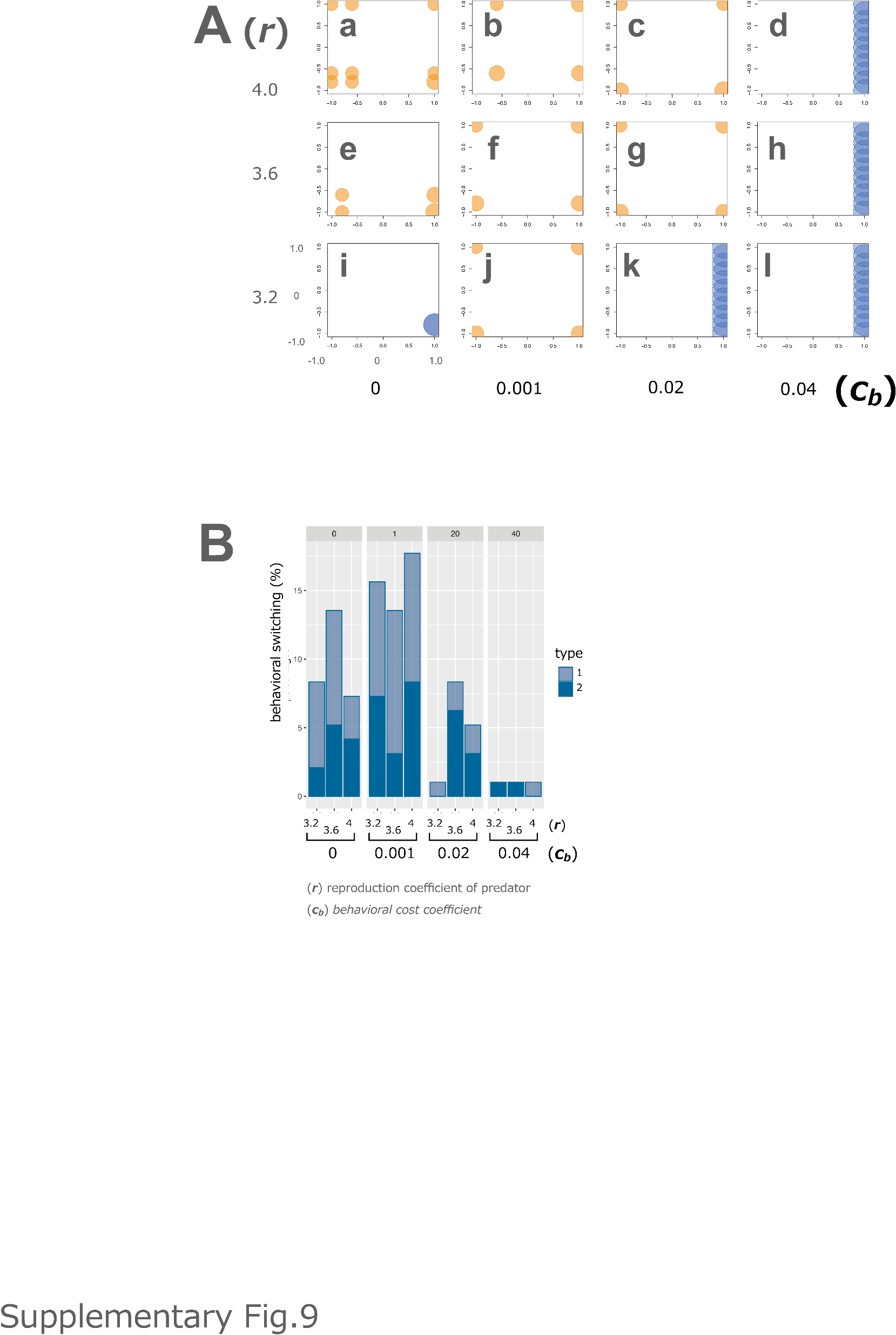

Supplement: S9 Fig — At Nash equilibrium, we observed two forms of probabilistic speed change: partial behavioral switching, in which only one agent changed speed probabilistically (type 1), and behavioral switching, in which both predator and prey did so (type 2). A illustrates both types of switching across various behavioral cost coefficients (cb) and the reproduction coefficients of predators (r) under a detection distance for both agents (Dw = Ds = 2). The upper (a–d), middle (e–h), and lower rows (i–l) correspond to r = 4.0, 3.6, and 3.2, respectively. The left (a, e, and i), the middle-left (b, f, and j), the middle-right (c, g, and k), and the right columns (d, h, and l) correspond to cb = 0, 0.001, 0.02, and 0.04, respectively. Circle positions on the x- and y-axes indicate the Nash equilibrium speed changes of prey (S―s) and predators (S―w), respectively, aligned with the payoff landscape coordinates. Orange circles indicate the cases where the predator and prey probabilistically perform their behaviors with increasing and decreasing speeds. The diameters of the orange circles are proportional to the occurrence probabilities of the behaviors (o―i) (0 <o―i < 1). The prey increase or decrease their speeds (either escape or freeze), whereas the predators do not; i.e., partial behavioral switching is observed in e. Both agents probabilistically increase or decrease their speeds in a, b, c, f, g, and j. Blue circles indicate the cases where S―i is uniquely determined (o―i = 1) in d, h, i, k, and l. B shows percentages of behavioral switching in one of the agents (type 1, partial behavioral switching) and both agents (type 2, behavioral switching) across all combinations of cb and r. (TIF) [file pcbi.1013730.s009.tif]

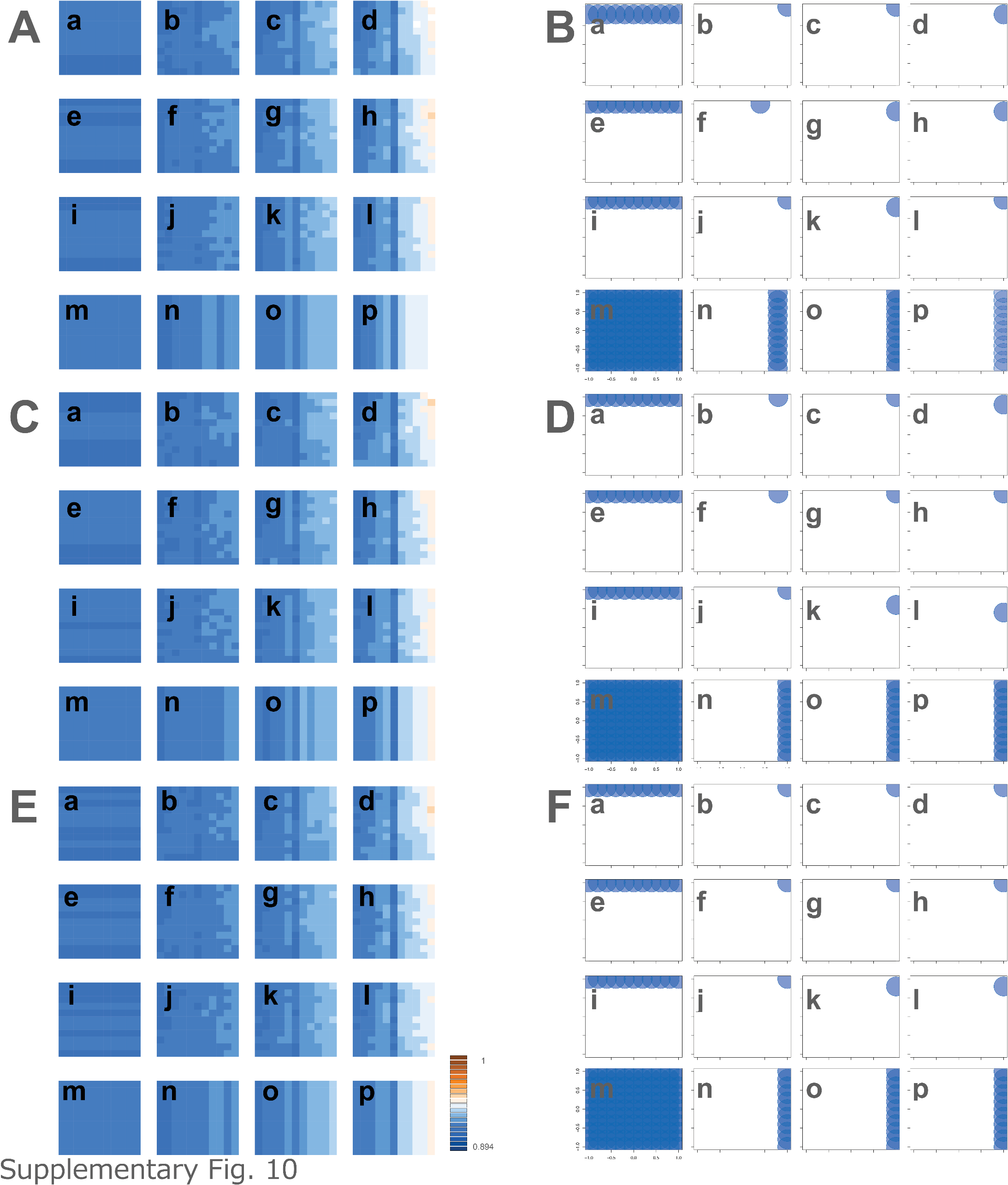

Supplement: S10 Fig — A and B show the landscapes of Pdiff and Nash equilibrium speed changes, respectively, under the reproduction coefficient of predators (r = 3.2). C and D show those under r = 3.6. E and F show those under r = 4.0. Each panel presents the strategic outcomes for different combinations of prey and predator speed changes. Pdiff values are color-coded: darker brown indicates higher payoff differences favoring prey, and darker blue indicates lower payoff differences (or advantage to predators). Circle positions in B, D, and F correspond to the Nash equilibrium speed changes (S―s on the x-axis, S―w on the y-axis). Circle diameters reflect the occurrence probabilities of the behaviors (o―i), with blue circles indicating unique equilibria (o―i = 1). (TIF) [file pcbi.1013730.s010.tif]

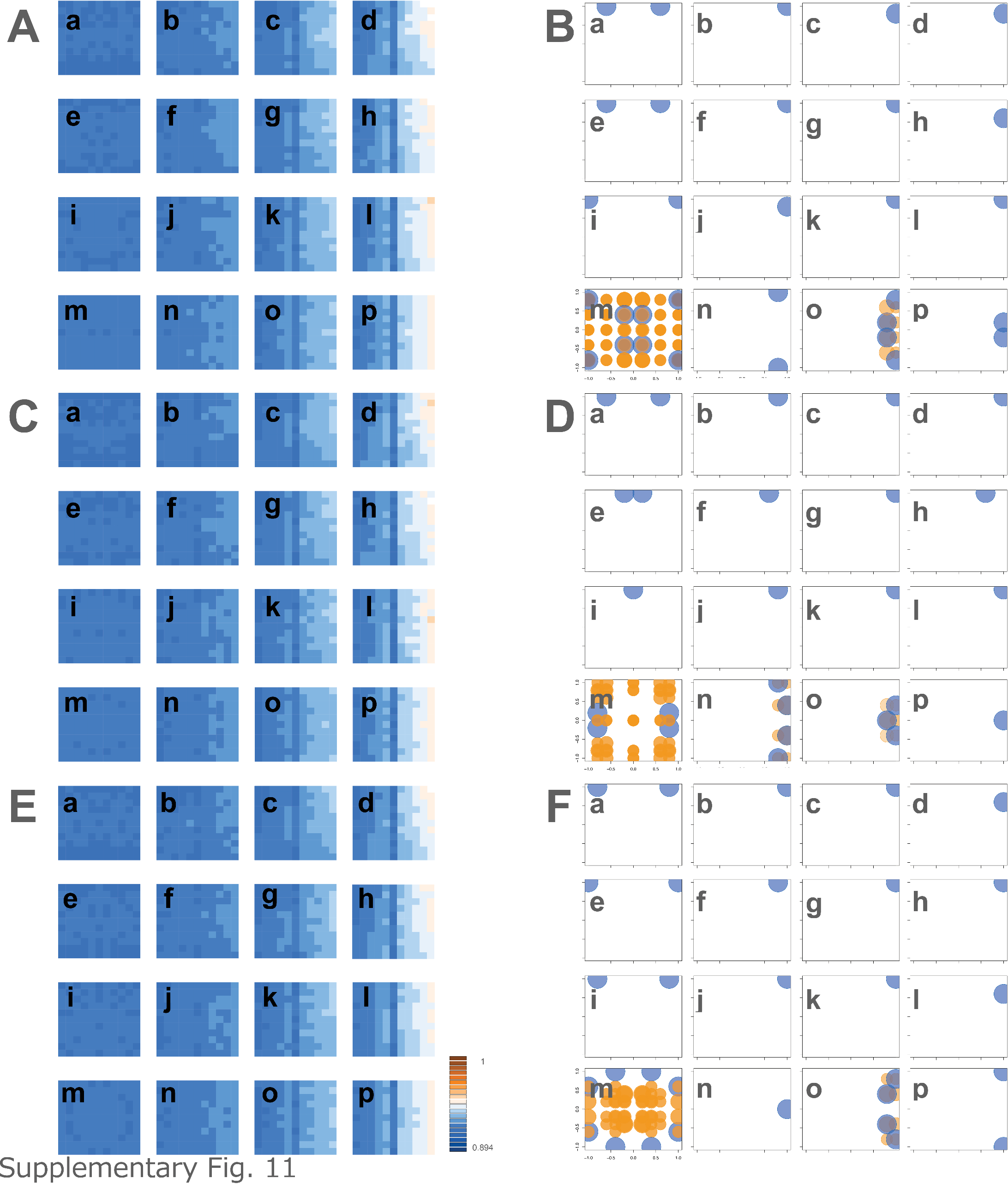

Supplement: S11 Fig — A and B show the landscapes of Pdiff and Nash equilibrium speed changes, respectively, under the reproduction coefficient of predators (r = 3.2). C and D show those under r = 3.6. E and F show those under r = 4.0. Circle diameters reflect the occurrence probabilities of the behaviors (o―i), with blue circles indicating unique equilibria (o―i = 1), and orange circles representing mixed strategies (0 <o―i < 1). (TIF) [file pcbi.1013730.s011.tif]

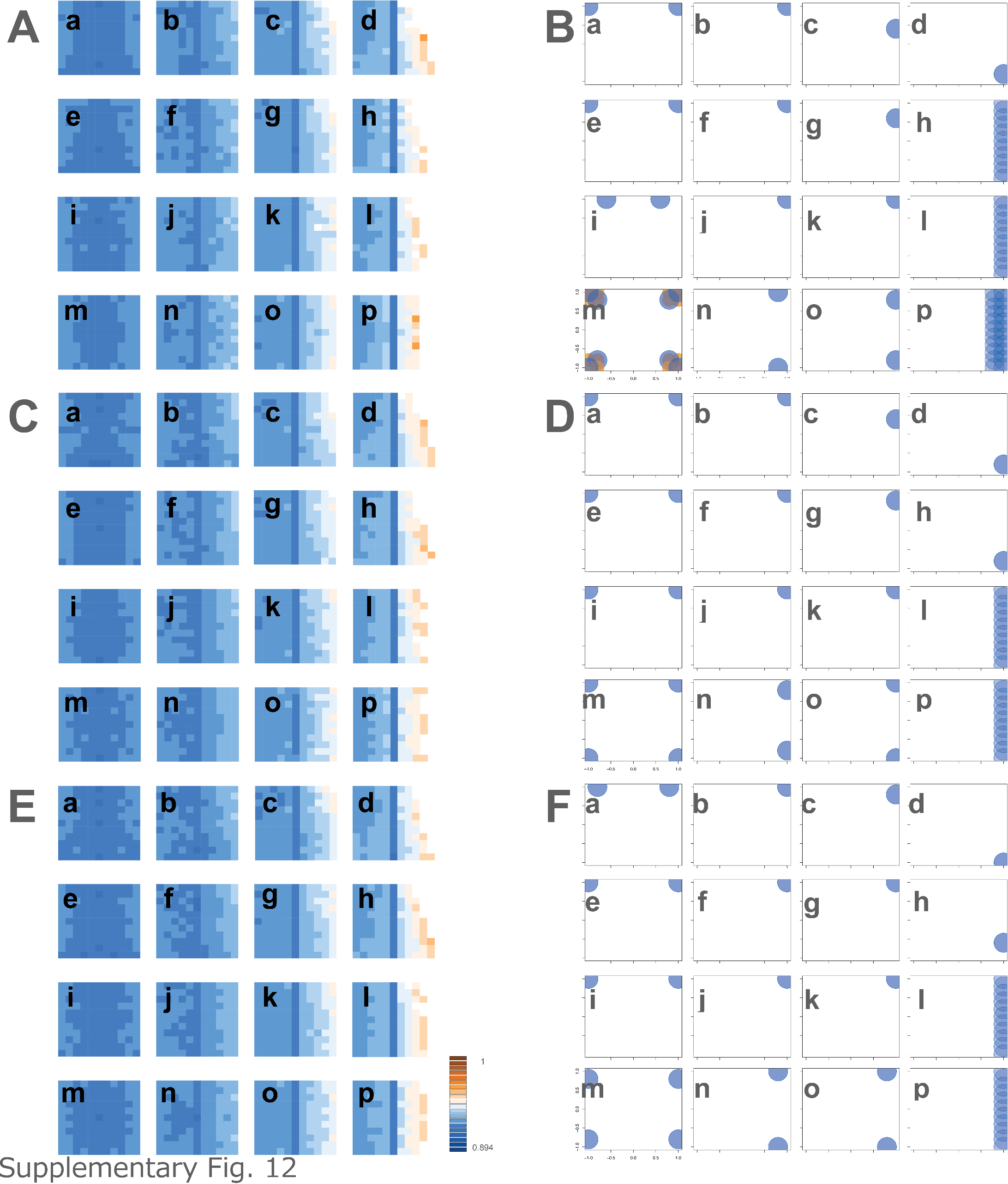

Supplement: S12 Fig — A and B show the landscapes of Pdiff and Nash equilibrium speed changes, respectively, under the reproduction coefficient of predators (r = 3.2). C and D show those under r = 3.6. E and F show those under r = 4.0. Circle diameters reflect the occurrence probabilities of the behaviors (o―i), with blue circles indicating unique equilibria (o―i = 1), and orange circles representing mixed strategies (0 <o―i < 1). (TIF) [file pcbi.1013730.s012.tif]

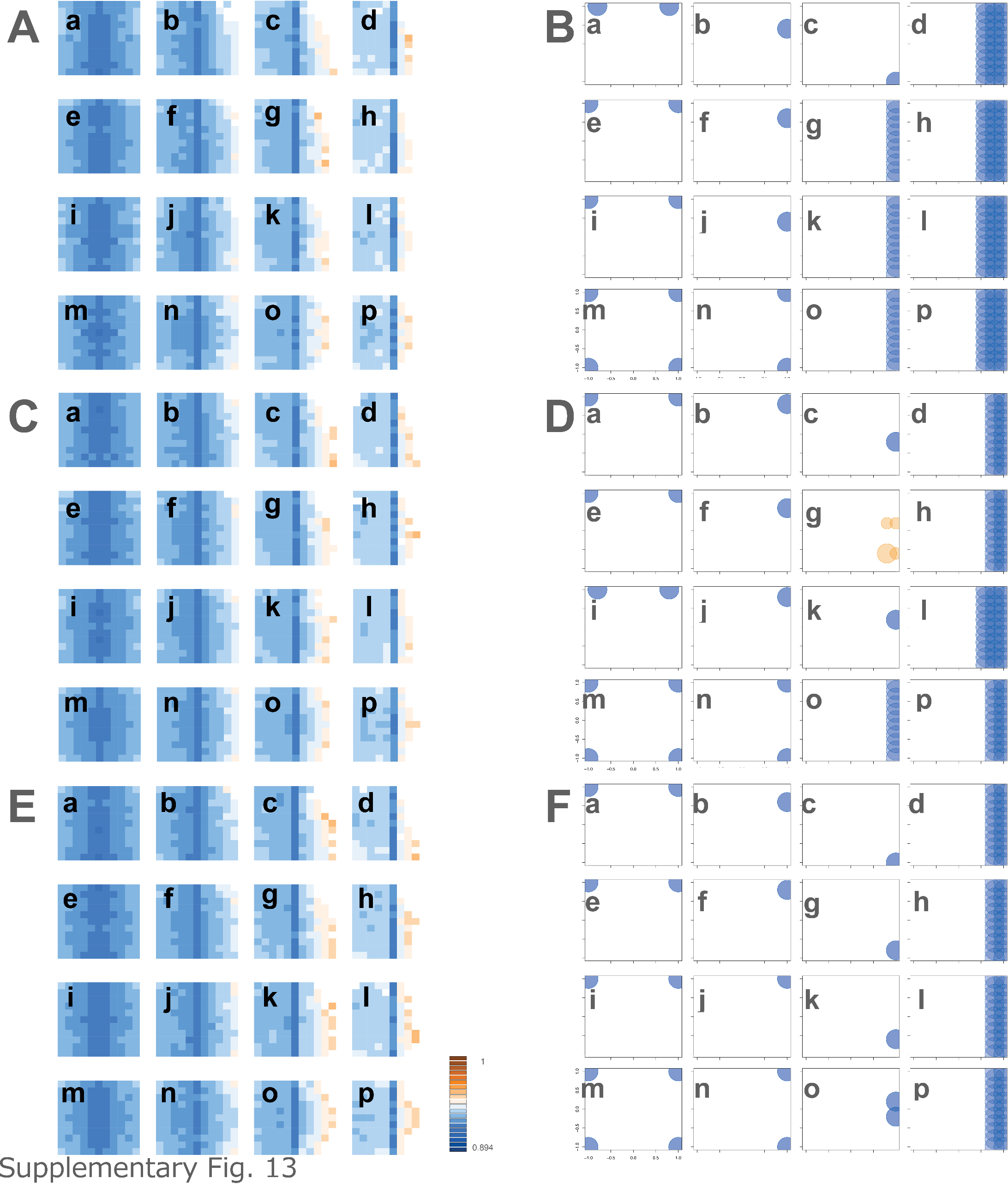

Supplement: S13 Fig — A and B show the landscapes of Pdiff and Nash equilibrium speed changes, respectively, under the reproduction coefficient of predators (r = 3.2). C and D show those under r = 3.6. E and F show those under r = 4.0. Circle diameters reflect the occurrence probabilities of the behaviors (o―i), with blue circles indicating unique equilibria (o―i = 1), and orange circles representing mixed strategies (0 <o―i < 1). (TIF) [file pcbi.1013730.s013.tif]

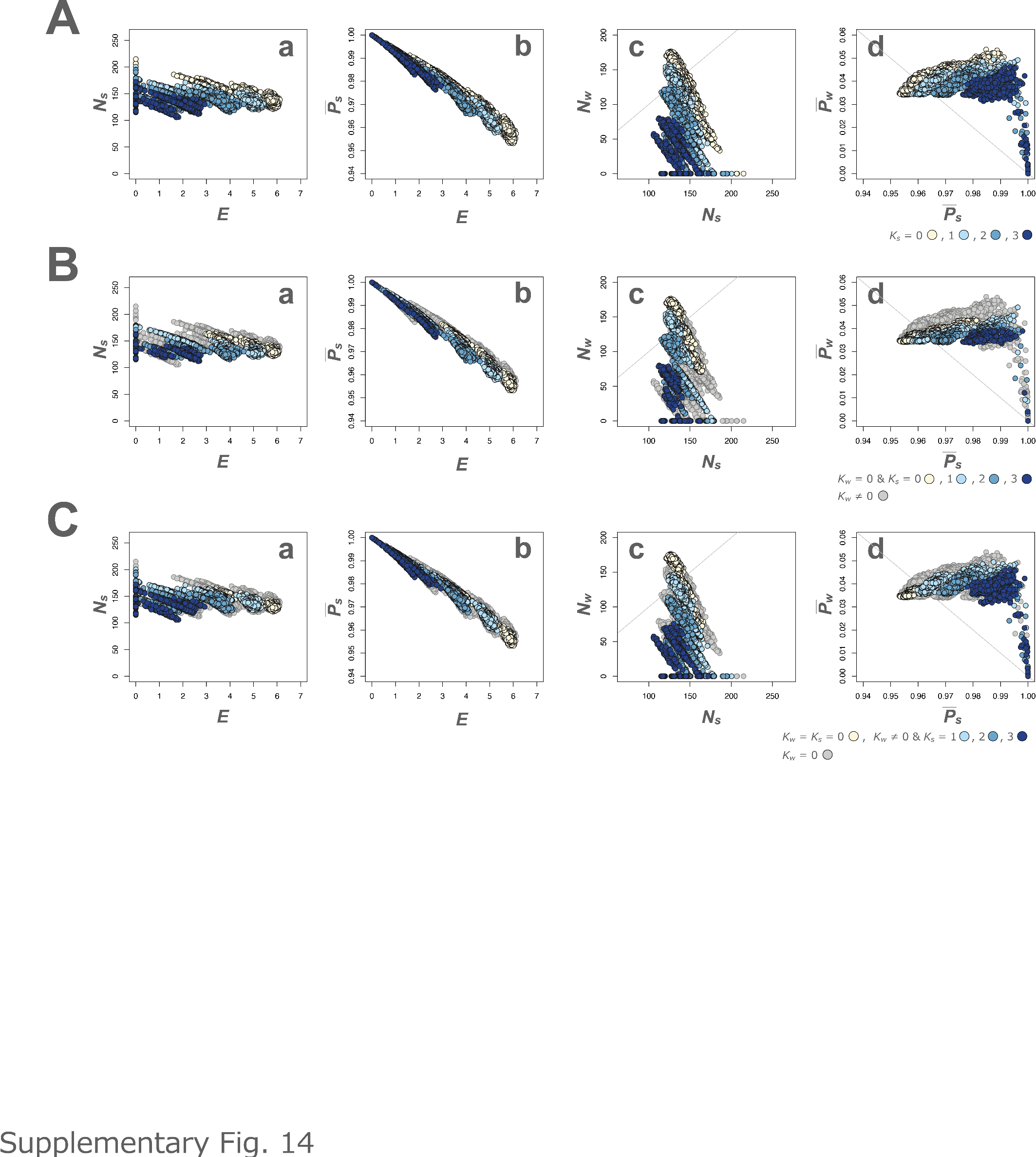

Supplement: S14 Fig — A shows scatter plots for all data of the behavioral cost coefficients (cb) and the reproduction coefficients of predators (r), illustrating the relationships among the encounter frequency (E), number of agents (Ni), and Nash equilibrium payoff (P¯i) (where i = w for wolf and s for sheep), based on actual measurements obtained by assigning Nash equilibrium speed changes. Aa shows a scatter plot of E on the x-axis against Ns on the y-axis, and Ab shows a scatter plot of E on the x-axis against P―s on the y-axis. Ac shows a scatter plot of Ns versus Nw, along the line Ns = Nw. Ad shows a scatter plot of P―s versus P―w, along the line P―s + P―w = 1. Beige, light blue, blue, and dark blue points represent Ks = 0, 0.1, 0.2, and 0.3, respectively, across all values of Kw (Kw = 0–0.3). Ba–Bd show the same scatter plots, with Kw = 0 highlighted in the colors and Kw ≠ 0 in gray. Ca–Cd show the same scatter plots, with Kw ≠ 0 highlighted in the colors and Kw = 0 in gray. Beige points represent Ks = Kw = 0 in B and C. Regardless of Kw, increasing Ks reduces E (dark blue points shift to the left side in Aa, Ba, and Ca), increases P―s (dark blue points shift to the upper-left side in Ab, Bb, and Cb, and dark blue points shift to the right side in Ad, Bd, and Cd), and reduces Nw (dark blue points shift to the lower side in Ac, Bc, and Cc). (TIF) [file pcbi.1013730.s014.tif]

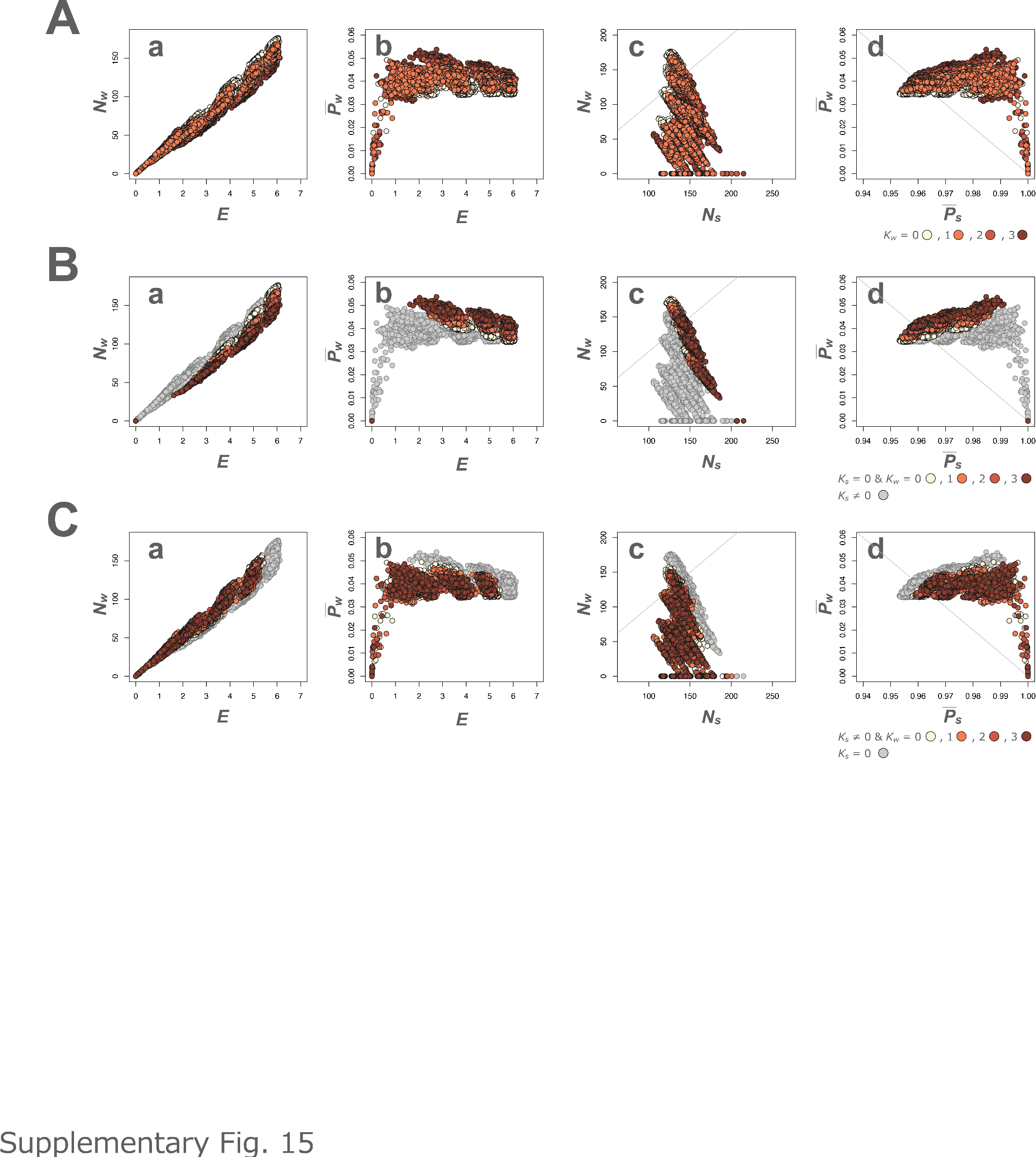

Supplement: S15 Fig — A shows scatter plots for all data of the behavioral cost coefficients (cb) and the reproduction coefficients of predators (r), illustrating the relationships among the encounter frequency (E), number of agents (Ni), and Nash equilibrium payoff (P¯i) (where i = w for wolf and s for sheep), based on actual measurements obtained by assigning Nash equilibrium speed changes. Aa and Ab show scatter plots of E on the x-axis against Nw and P¯w on the y-axis, respectively. Ac shows a scatter plot of Ns versus Nw, along the line Ns = Nw. Ad shows a scatter plot of P―s versus P―w, along the line P―s + P―w = 1. Beige, orange, brown, and dark brown points represent, Kw = 0, 0.1, 0.2, and 0.3, respectively, across all values of Ks (Ks = 0–0.3). Ba–Bd show the same scatter plots, with Ks = 0 highlighted in the colors and Ks ≠ 0 in gray. When Ks = 0, as Kw increases, Nw decreases (dark brown points shift to the lower side in Ba). P―w increases (dark brown points shift to the upper side in Bb). Ns increases and Nw decreases (dark brown points shift to the lower-right side in Bc). P―s and P―w increase (dark brown points shift to the upper-right side in Bd). Ca–Cd show the same scatter plots, with Ks ≠ 0 highlighted in the colors and Ks = 0 in gray. (TIF) [file pcbi.1013730.s015.tif]

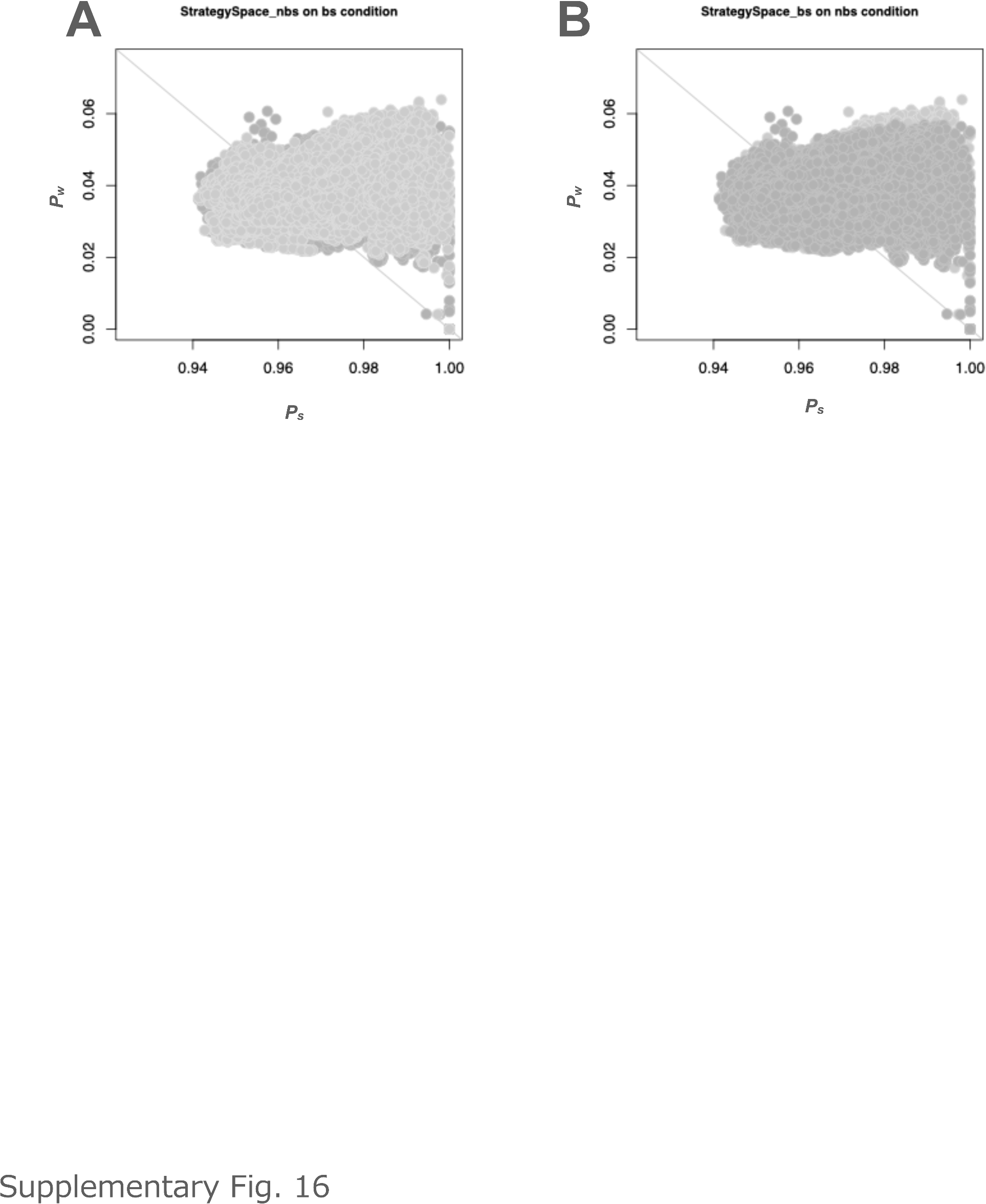

Supplement: S16 Fig — The pooled Ps and Pw data calculated across all Si combinations under Di combinations where behavioral switching occurred are designated as “bs” (behavioral switching) [Ds and Dw are (1, 1), (1, 2), (1, 3), (2, 2), (2, 3), (3, 1), (3, 2), and (3, 3) for all cb and r], and those under all other Di combinations as “nbs” (non-behavioral switching). The full range of payoffs from all behaviors is plotted on a scatter plot with Ps on the x-axis and Pw on the y-axis. The data corresponding to bs are shown in dark gray, and those for nbs in light gray plots. Ranges of each region represent the payoff spaces of bs and nbs. A shows a scatter plot with nbs on top of bs. B shows bs on top of nbs. No marked difference is observed between the two payoff spaces. (TIF) [file pcbi.1013730.s016.tif]
