# Supplementary material for: Nash equilibrium of attack and defense behaviors between predators and prey
Source: PLoS Comput Biol. 2025 Nov 21;21(11):e1013730. doi: 10.1371/journal.pcbi.1013730 (PMC12671891; doi:10.1371/journal.pcbi.1013730)
Supplement: S1 Table — (DOCX) [file pcbi.1013730.s017.docx]

**S1 Table**

**Constant values used in simulations involving multiple agents with the sensory-motor algorithm**

| **Constants** |  |
| --- | --- |
| Initial number of predators (wolf) | 60 |
| Initial number of prey (sheep) | 60 |
| Energy gained by a predator eating a prey (wolf-gain-from-food) | 8 |
| Energy gained by a prey from eating grass (sheep-gain-from-food) | 1 |
| Reproduction coefficient of prey (sheep-reproduce) | 9 |
| Time for grass to grow (grass-regrowth-time) | 30 |
| Movement speed | 1 |
| Coefficient of sensory trait (factor sensitivity, *f*) | 10 |
| Cost associated with basal metabolism (*c_e_*) | 0.1 |
| Movement cost coefficient (*c_m_*) | 0.15 |
