# Supplementary material for: Nash equilibrium of attack and defense behaviors between predators and prey
Source: PLoS Comput Biol. 2025 Nov 21;21(11):e1013730. doi: 10.1371/journal.pcbi.1013730 (PMC12671891; doi:10.1371/journal.pcbi.1013730)
Supplement: S2 Table — (DOCX) [file pcbi.1013730.s018.docx]

**S2 Table**

**Variable values used in simulations involving multiple agents with the sensory-motor algorithm**

| **Variables** | **values** |
| --- | --- |
| Sensory trait (*d*) |  |
| *d* of wolves (*d_w_*) | 0, 0.1, 0.2, 0.3 |
| *d* of sheep (*d_s_*) | 0, 0.1, 0.2, 0.3 |
| Distance to detect the opponent (*D*) * |  |
| *D* of wolves (*D_w_*) | 0, 1, 2, 3 |
| *D* of sheep (*D_s_*) | 0, 1, 2, 3 |
| Motor trait (*S*) for the magnitude of speed change (*S*)† |  |
| *S* of wolves: *S_w_* | -1.0, -0.8, -0.6, -0.4, -0.2, 0.0, 0.2, 0.4, 0.6, 0.8, 1.0 |
| *S* of sheep: *S_s_* | -1.0, -0.8, -0.6, -0.4, -0.2, 0.0, 0.2, 0.4, 0.6, 0.8, 1.0 |
|  |  |
| Behavioral cost coefficient (*c_b_*)‡ | 0, 0.001, 0.02, 0.04 |
| Reproductive coefficient of predator (*r*) | 3.2, 3.6, 4.0 |

*Distance to detect an opponent (*D_i_*) (*i* = *w*, *s*) is *d_i_* multiplied by factor-sensitivity, *f* (*D_i_* = *d_i_* × *f*, where *f* = 10).

†When agents detect the opponent within *D_i_*, their movement changes to 1 + *S_i_*.

‡ Cost dependent on the sensory and motor traits is the sum of the sensory and motor costs. The sensory cost is *d_i_* multiplied by behavioral cost coefficient (*c_b_*). Th motor cost is absolute value of *S_i_* (abs(*S_i_*)) multiplied by *c_b_*. Thus, it is (*d_i_* + (abs(*S_i_*))**c_b_*.
