# Supplementary material for: Nash equilibrium of attack and defense behaviors between predators and prey
Source: PLoS Comput Biol. 2025 Nov 21;21(11):e1013730. doi: 10.1371/journal.pcbi.1013730 (PMC12671891; doi:10.1371/journal.pcbi.1013730)
Supplement: S4 Table — (DOCX) [file pcbi.1013730.s020.docx]

**S4 Table**

**Variable values used in simulations involving multiple agents with the non-sensory motor algorithm**

| **Variables** | **values** |
| --- | --- |
| Probability of speed change (*K*) |  |
| *K* of wolves (*K_w_*) | 0, 0.1, 0.2, 0.3 |
| *K* of sheep (*K_s_*) | 0, 0.1, 0.2, 0.3 |
|  |  |
| Magnitude of speed change (*S*)† |  |
| *S* of wolves: *S_w_* | -1.0, -0.8, -0.6, -0.4, -0.2, 0.0, 0.2, 0.4, 0.6, 0.8, 1.0 |
| *S* of sheep: *S_s_* | -1.0, -0.8, -0.6, -0.4, -0.2, 0.0, 0.2, 0.4, 0.6, 0.8, 1.0 |
|  |  |
| Behavioral cost coefficient (*c_b_*)‡ | 0, 0.001, 0.02, 0.04 |
| Reproductive coefficient of predator (*r*) | 3.2, 3.6, 4.0 |

†Agents change their speed to 1 + *S_i_* with a probability of *K_i_*.

‡ The costs dependent on the probability and the magnitude of speed change are defined as the sum of *K_i_* and absolute value of the motor trait (abs(S_i_)) multiplied by *c_b_*. Thus, it is (*K_i_* + (abs(*S_i_*))**c_b_*.
