# Supplementary material for: Nash equilibrium of attack and defense behaviors between predators and prey
Source: PLoS Comput Biol. 2025 Nov 21;21(11):e1013730. doi: 10.1371/journal.pcbi.1013730 (PMC12671891; doi:10.1371/journal.pcbi.1013730)
Supplement: S11 Data — (PDF) [file pcbi.1013730.s038.pdf]

Appearance of source data for the sensory-motor algorithm between one predator and one prey

Source data

S1_Dataset.xlsx

for payoff matrices in the grid world

S_s prey change in speed

predator change in speed S_w

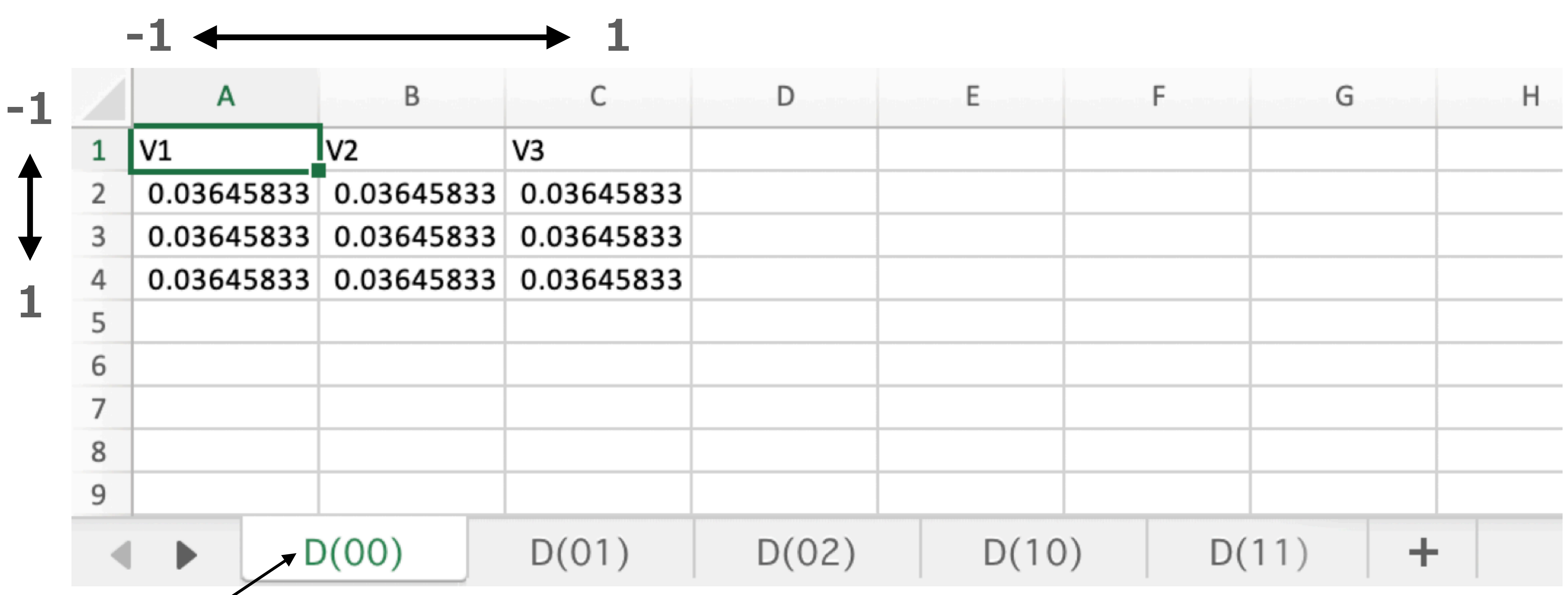

distance to detect the oppoent

(00) means D_s = 0 and D_w = 0

Appearance of source data for the sensory-motor algorithm between one predator and one prey

Source data

S2_Dataset.xlsx

for Nash equilibrium solutions in the grid world

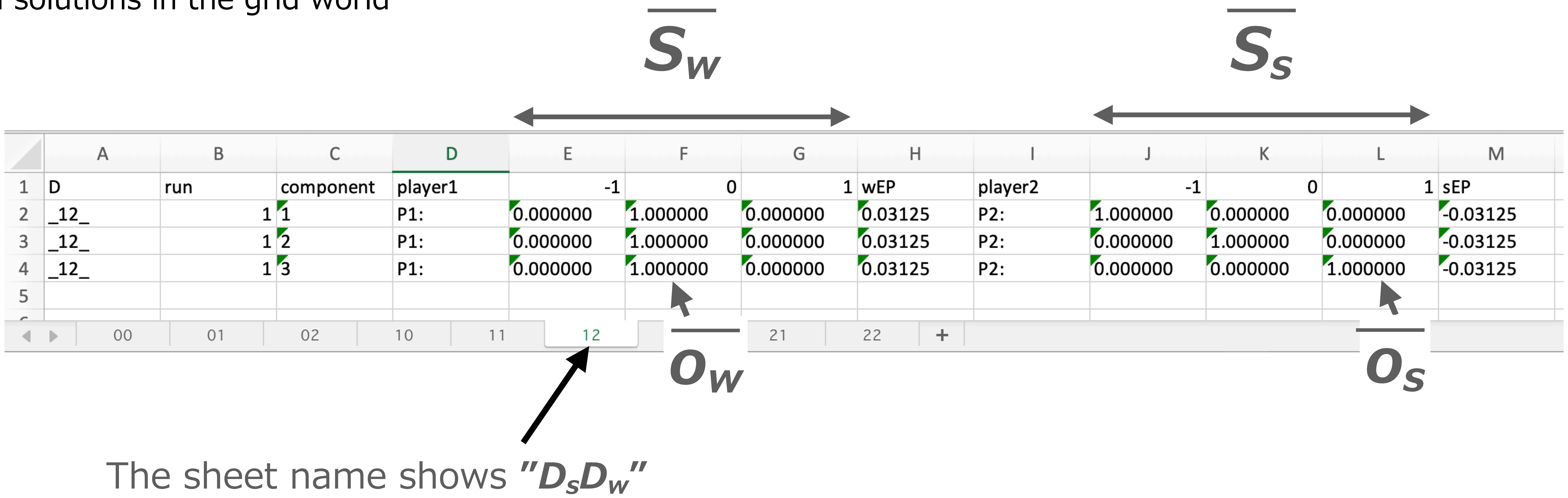

	A	B	C	D	E	F	G	H	I	J	K	L	M
1	D	run	component	player1	-1	0	1	wEP	player2	-1	0	1	sEP
2	_12_	1	1	P1:	0.000000	1.000000	0.000000	0.03125	P2:	1.000000	0.000000	0.000000	-0.03125
3	_12_	1	2	P1:	0.000000	1.000000	0.000000	0.03125	P2:	0.000000	1.000000	0.000000	-0.03125
4	_12_	1	3	P1:	0.000000	1.000000	0.000000	0.03125	P2:	0.000000	0.000000	1.000000	-0.03125
5													

Appearance of source data for the sensory-motor algorithm between multiple agents

Source data

S3_Dataset.xlsx

for payoff matrices in the NetLogo world

There are cases where blanks were included in sheep payoff matrices.
These blanks represented NA, where sheep were extinct by predation. After the extinction of prey, predator became extinct; their payoff became zero.
For drawing, the landscapes are represented by white blanks。For Nash equilibrium, one needs to calculate by substituting 0 (zero) for blank.

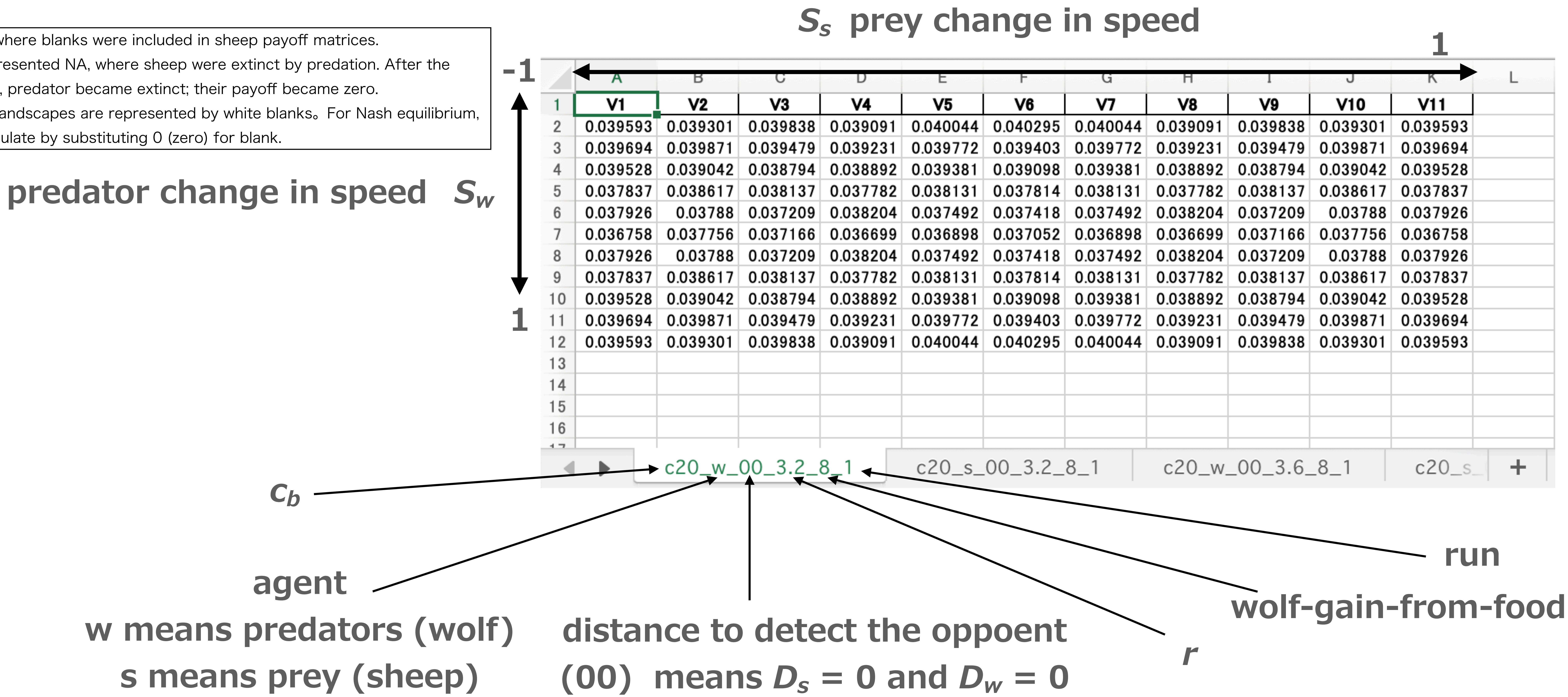

	A	B	C	D	E	F	G	H	I	J	K
1	V1	V2	V3	V4	V5	V6	V7	V8	V9	V10	V11
2	0.039593	0.039301	0.039838	0.039091	0.040044	0.040295	0.040044	0.039091	0.039838	0.039301	0.039593
3	0.039694	0.039871	0.039479	0.039231	0.039772	0.039403	0.039772	0.039231	0.039479	0.039871	0.039694
4	0.039528	0.039042	0.038794	0.038892	0.039381	0.039098	0.039381	0.038892	0.038794	0.039042	0.039528
5	0.037837	0.038617	0.038137	0.037782	0.038131	0.037814	0.038131	0.037782	0.038137	0.038617	0.037837
6	0.037926	0.03788	0.037209	0.038204	0.037492	0.037418	0.037492	0.038204	0.037209	0.03788	0.037926
7	0.036758	0.037756	0.037166	0.036699	0.036898	0.037052	0.036898	0.036699	0.037166	0.037756	0.036758
8	0.037926	0.03788	0.037209	0.038204	0.037492	0.037418	0.037492	0.038204	0.037209	0.03788	0.037926
9	0.037837	0.038617	0.038137	0.037782	0.038131	0.037814	0.038131	0.037782	0.038137	0.038617	0.037837
10	0.039528	0.039042	0.038794	0.038892	0.039381	0.039098	0.039381	0.038892	0.038794	0.039042	0.039528
11	0.039694	0.039871	0.039479	0.039231	0.039772	0.039403	0.039772	0.039231	0.039479	0.039871	0.039694
12	0.039593	0.039301	0.039838	0.039091	0.040044	0.040295	0.040044	0.039091	0.039838	0.039301	0.039593

Appearance of source data for the sensory-motor algorithm between multiple agents

Source data

S4_Dataset.xlsx

for Nash equilibrium solutions in the NetLogo world

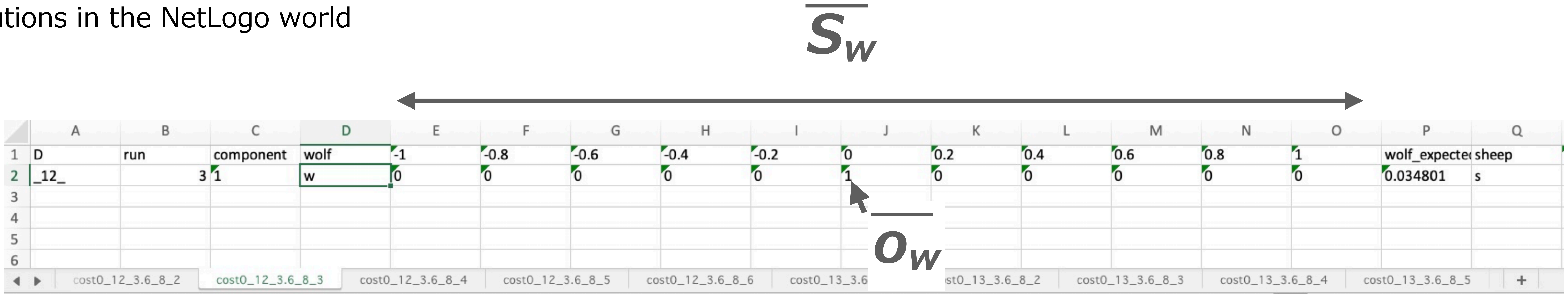

	A	B	C	D	E	F	G	H	I	J	K	L	M	N	O	P	Q
1	D	run	component	wolf	-1	-0.8	-0.6	-0.4	-0.2	0	0.2	0.4	0.6	0.8	1	wolf_expecte	sheep
2	_12_	3	1	w	0	0	0	0	0	1	0	0	0	0	0	0.034801	s
3																	
4																	
5																	
6																	

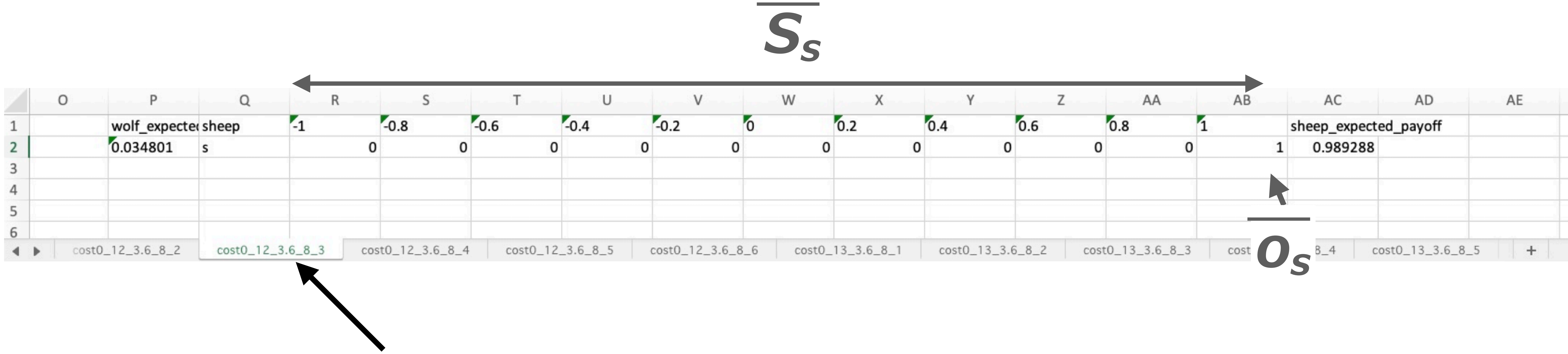

	O	P	Q	R	S	T	U	V	W	X	Y	Z	AA	AB	AC	AD	AE
1		wolf_expecte	sheep	-1	-0.8	-0.6	-0.4	-0.2	0	0.2	0.4	0.6	0.8	1	sheep_expected_payoff		
2		0.034801	s	0	0	0	0	0	0	0	0	0	0	1	0.989288		
3																	
4																	
5																	
6																	

The sheet name shows **"c_b"_"D_sD_w"_"r"_"wolf-gain-from-food"_"run"**.

Appearance of source data for the non-sensory motor algorithm between multiple agents

Source data

S7_Dataset.xlsx

for for payoff matrices in the NetLogo world

S_s prey amount of speed change

-1 ⟷ **1**

predator amount of speed change

S_w (**-1** ⟷ **1**)

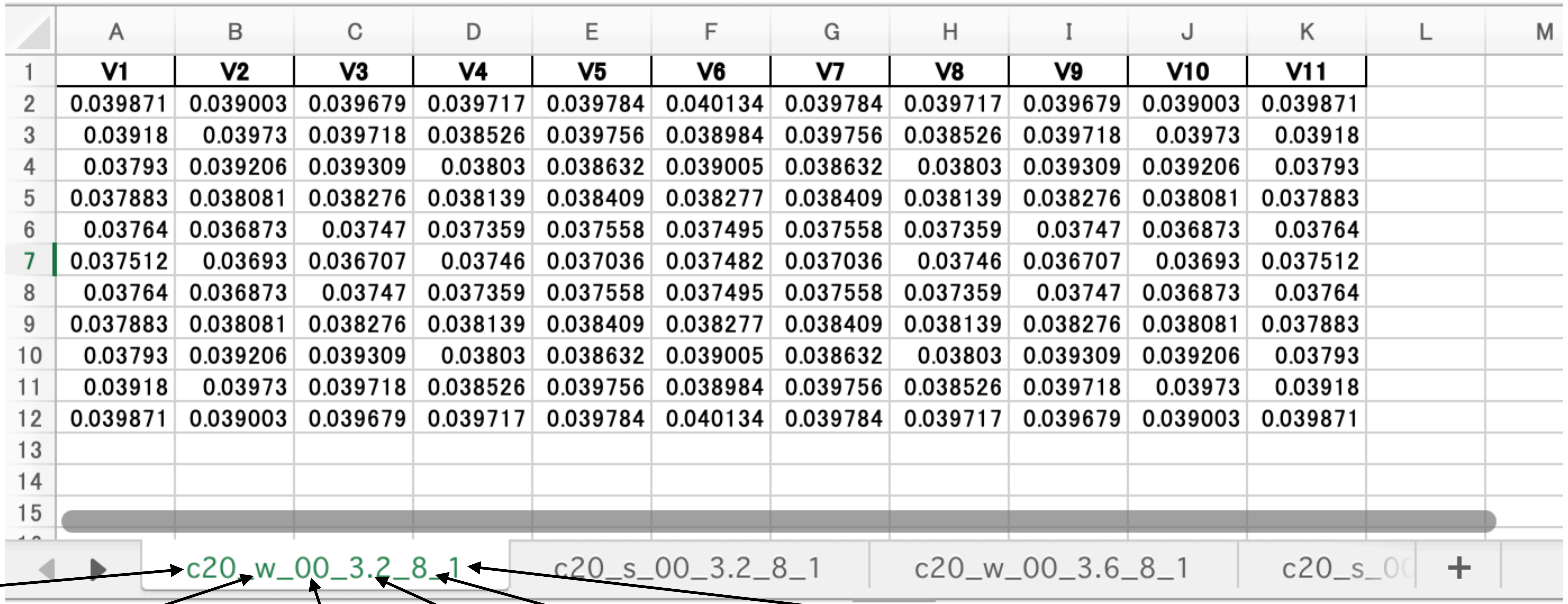

	V1	V2	V3	V4	V5	V6	V7	V8	V9	V10	V11
2	0.039871	0.039003	0.039679	0.039717	0.039784	0.040134	0.039784	0.039717	0.039679	0.039003	0.039871
3	0.03918	0.03973	0.039718	0.038526	0.039756	0.038984	0.039756	0.038526	0.039718	0.03973	0.03918
4	0.03793	0.039206	0.039309	0.03803	0.038632	0.039005	0.038632	0.03803	0.039309	0.039206	0.03793
5	0.037883	0.038081	0.038276	0.038139	0.038409	0.038277	0.038409	0.038139	0.038276	0.038081	0.037883
6	0.03764	0.036873	0.03747	0.037359	0.037558	0.037495	0.037558	0.037359	0.03747	0.036873	0.03764
7	0.037512	0.03693	0.036707	0.03746	0.037036	0.037482	0.037036	0.03746	0.036707	0.03693	0.037512
8	0.03764	0.036873	0.03747	0.037359	0.037558	0.037495	0.037558	0.037359	0.03747	0.036873	0.03764
9	0.037883	0.038081	0.038276	0.038139	0.038409	0.038277	0.038409	0.038139	0.038276	0.038081	0.037883
10	0.03793	0.039206	0.039309	0.03803	0.038632	0.039005	0.038632	0.03803	0.039309	0.039206	0.03793
11	0.03918	0.03973	0.039718	0.038526	0.039756	0.038984	0.039756	0.038526	0.039718	0.03973	0.03918
12	0.039871	0.039003	0.039679	0.039717	0.039784	0.040134	0.039784	0.039717	0.039679	0.039003	0.039871

c_b

run

agent
w means predators (wolf)
s means prey (sheep)

probability of speed chang
_00_ means K_s = 0 and K_w = 0

wolf-gain-from-food

wolf-reproduce

Appearance of source data for the non-sensory motor algorithm between multiple agents

Source data

S8_Dataset.xlsx

for Nash equilibrium solutions in the NetLogo world

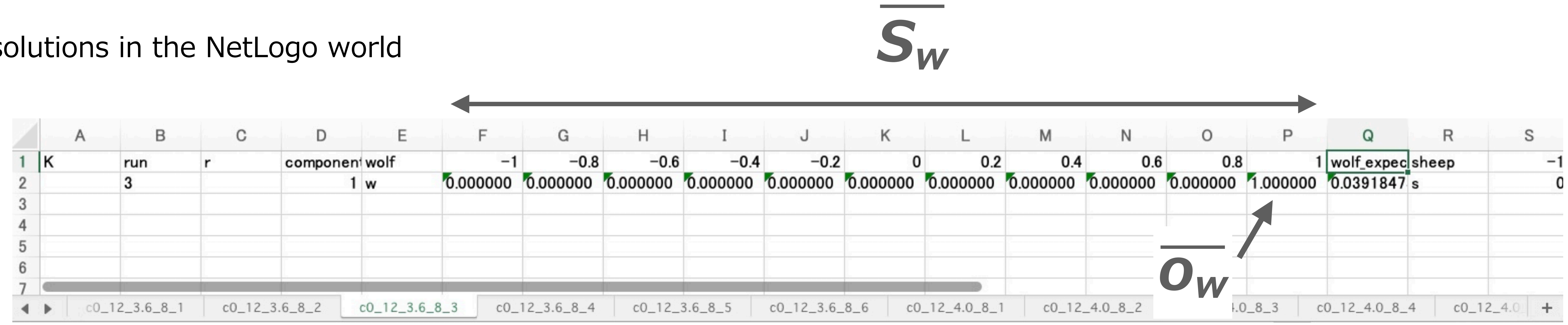

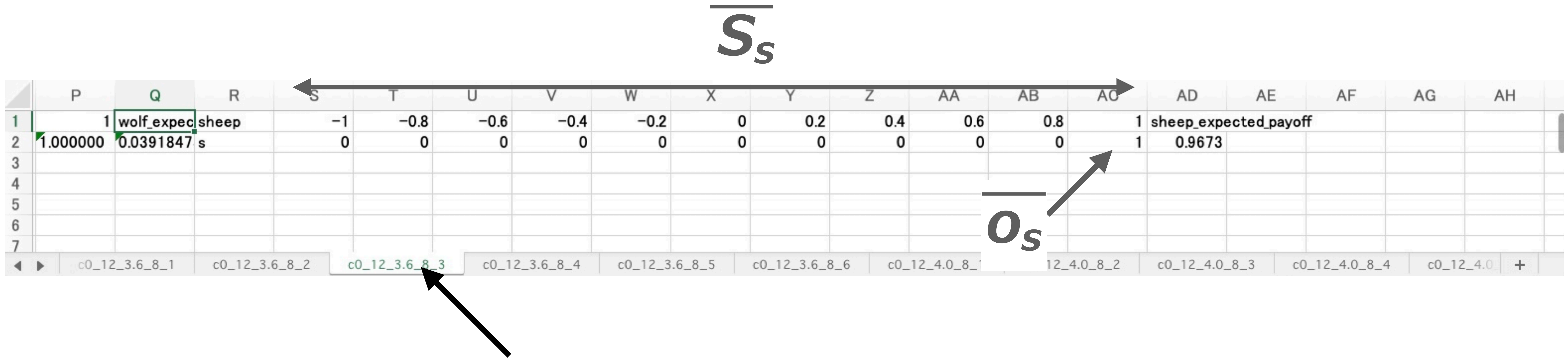

The sheet name shows **"c_b"_"K_sK_w"_"r"_"wolf-gain-from-food"_"run"**.
