# Supplementary material for: Nash equilibrium of attack and defense behaviors between predators and prey
Source: PLoS Comput Biol. 2025 Nov 21;21(11):e1013730. doi: 10.1371/journal.pcbi.1013730 (PMC12671891; doi:10.1371/journal.pcbi.1013730)
Supplement: Attachment — Submitted filename: PCOMPBIOL-D-24-01911_annotated.pdf [file pcbi.1013730.s041.pdf]

PLOS Computational Biology

Nash equilibrium of attack and defense behaviors between predators and prey

--Manuscript Draft--

**Manuscript Number:**	PCOMPBIOL-D-24-01911
**Full Title:**	Nash equilibrium of attack and defense behaviors between predators and prey
**Short Title:**	Nash equilibrium of attack and defense
**Article Type:**	Research Article
**Keywords:**	Nash equilibrium; attack; defense; predator; prey; individual-based model; algorithm; game theory
**Abstract:**	Uncovering the functions of neuronal circuits in attack/defense behaviors requires an algorithmic and computational understanding. We modeled and analyzed these behaviors with game theory. A sensory-motor algorithm without directionality was sufficient for predators and prey (agents) to modify their payoffs based on encounter probabilities and generate Nash equilibrium attack/defense behaviors. Moreover, the sensory-motor algorithm was not necessary because a simplified single-element motor algorithm was sufficient to achieve Nash equilibrium, generating a function comparable to these behaviors. Despite their similarity, these algorithms had distinct adaptive significance. The sensory-motor algorithm produced substantially greater prey payoffs. These demonstrated the fundamental mechanisms for yielding attack/defense behaviors; implementing these algorithms computes agents' payoffs and achieves the Nash equilibrium behaviors. These findings suggest that the evolutionary mechanisms underlying attack/defense behaviors are intrinsic to the model environments where agents are subjected to selection. Therefore, the present approach is valuable for an experimental understanding of their evolution.
**Additional Information:**	
**Question**	**Response**
**Government Employee**<br><br>Are you or any of the contributing authors an employee of the United States government?<br><br>Manuscripts authored by one or more US Government employees are not copyrighted, but are licensed under a CC0 Public Domain Dedication, which allows unlimited distribution and reuse of the article for any lawful purpose. This is a legal requirement for US Government employees.<br><br>This will be typeset if the manuscript is accepted for publication.	No - No authors are employees of the U.S. government.
**Financial Disclosure**<br><br>Enter a financial disclosure statement that describes the sources of funding for the work included in this submission. Review	Yes

the submission guidelines for detailed requirements. View published research articles from *PLOS Computational Biology* for specific examples.<br><br>This statement is required for submission and **will appear in the published article** if the submission is accepted. Please make sure it is accurate.<br><br>**Funded studies**<br>Enter a statement with the following details:<br>• Initials of the authors who received each award<br>• Grant numbers awarded to each author<br>• The full name of each funder<br>• URL of each funder website<br>• Did the sponsors or funders play any role in the study design, data collection and analysis, decision to publish, or preparation of the manuscript?<br><br>Did you receive funding for this work?	
Please add funding details.<br>as follow-up to "**Financial Disclosure**<br><br>Enter a financial disclosure statement that describes the sources of funding for the work included in this submission. Review the submission guidelines for detailed requirements. View published research articles from *PLOS Computational Biology* for specific examples.<br><br>This statement is required for submission and **will appear in the published article** if the submission is accepted. Please make sure it is accurate.	HI JP21K06371<br>TN JP22K07367<br>Grant-in-Aid for Scientific Research(C) of Japan Society for the Promotion of Science (JSPS KAKENHI)<br>https://www.jsps.go.jp/j-grantsinaid/index.html

**Funded studies**<br>Enter a statement with the following details:<br>• Initials of the authors who received each award<br>• Grant numbers awarded to each author<br>• The full name of each funder<br>• URL of each funder website<br>• Did the sponsors or funders play any role in the study design, data collection and analysis, decision to publish, or preparation of the manuscript?<br><br>Did you receive funding for this work?"	
Please select the country of your main research funder (please select carefully as in some cases this is used in fee calculation).<br>as follow-up to "**Financial Disclosure**<br><br>Enter a financial disclosure statement that describes the sources of funding for the work included in this submission. Review the submission guidelines for detailed requirements. View published research articles from *PLOS Computational Biology* for specific examples.<br><br>This statement is required for submission and **will appear in the published article** if the submission is accepted. Please make sure it is accurate.<br><br>**Funded studies**<br>Enter a statement with the following details:<br>• Initials of the authors who received each award<br>• Grant numbers awarded to each author<br>• The full name of each funder<br>• URL of each funder website<br>• Did the sponsors or funders play any role in the study design, data collection and analysis, decision to publish, or preparation of the manuscript?<br><br>Did you receive funding for this work?"	JAPAN - JP
**Competing Interests**	The authors declare no competing interests.

On behalf of all authors, disclose any competing interests that could be perceived to bias this work.<br><br>This statement will be typeset if the manuscript is accepted for publication.<br><br>Review the instructions link below and PLOS Computational Biology's competing interests policy to determine what information must be disclosed at submission.	
**Data and Code Availability**<br><br>From the time of publication, Authors are required to make fully available and without restriction all data and computational code underlying their findings. Please see our PLOS Data Policy page for detailed policy information, and our Code Sharing page for specific information on code sharing.<br><br>A **Data Availability Statement**, detailing where the data (and code, if applicable) can be accessed, is required at first submission. Insert your Data Availability Statement in the box below. The statement you provide **will be published in the article**, if accepted.<br><br>Please see the Data Reporting section of our submission guidelines for instructions on what you need to include in your Data Availability Statement.<br><br>If the data and code are all contained in your submission files, please state: *All relevant data are within the manuscript and its Supporting Information files.*<br><br>**PLOS allows rare exemptions to address legal and ethical concerns.** If you have legal or ethical restrictions, please detail these in your Data Availability Statement below for the Journal team to consider.	All supplemental programs and source data its supporting information files are stored in Figshare:<br>https://doi.org/10.6084/m9.figshare.27166392.v1,<br>https://doi.org/10.6084/m9.figshare.27166377.v1,<br>and<br>https://doi.org/10.6084/m9.figshare.27166365.v1.

Dear Editor,

Below is the response from the editor regarding my submission (PBIOLOGY-D-24-03015). As stated, the manuscript did not proceed to the reviewer stage as a result of the editorial assessment. The content of the submitted manuscript remains unchanged from PBIOLOGY-D-24-03015.

Thank you for your consideration.

Sincerely,

Hiroyuki Ichijo

##########
件名: Your PLOS Biology Submission (PBIOLOGY-D-24-03015) - [EMID:728894b91dca863c]
送信者: "Roland G Roberts" <em@editorialmanager.com>
宛先: "Hiroyuki Ichijo" <ichijo@med.u-toyama.ac.jp>
送信日時: 2024 年 10 月 24 日(木) 20:04:38

Dear Dr Ichijo,

Thank you for submitting your manuscript entitled "Nash equilibrium of attack and defense behaviors between predators and prey" to PLOS Biology for consideration. I regret to inform you that after careful discussion within the editorial team, we have decided that we cannot consider it for publication.

We were interested to read about your use of simulation to explore the algorithmic requirements to recover a Nash equilibrium between predators and prey, and we appreciate the potential implications of your findings for our ability to understand the underlying neuronal processes underlying predation and evasion. Regretfully, however, we do not feel that your study represents the strength of advance that we require for publication in PLOS Biology.

As you may know, we decline a substantial proportion of manuscripts without sending them to reviewers, so that they may be sent elsewhere without delay. In such cases, even if reviewers were to certify the manuscript as technically correct, we consider that the work does not represent the type of advance that PLOS Biology seeks to publish. This editorial assessment is based on considerations such as the degree of advance provided and the breadth of potential interest of the research question to our broad readership.

While we cannot consider your manuscript for publication in PLOS Biology, we suggest that you consider transferring it to PLOS Computational Biology. The PLOS journals are editorially independent, so we cannot guarantee it will be reviewed there.

If you would like to transfer your manuscript, as suggested, please click the following link:

https://www.editorialmanager.com/pbiology/l.asp?i=1104061&l=0IJFTAM1

If you do NOT wish to transfer your manuscript, please click this link to decline:

https://www.editorialmanager.com/pbiology/l.asp?i=1104062&l=02D20BHR

Please note, you can log into the submission sites with the same login that you used to submit to this journal.

Should you choose to transfer your submission you will receive a confirmation email within 24-48 hours after accepting the transfer. If you have any questions, please feel free to contact the journal at plosbiology@plos.org.

Thank you again for your interest in PLOS Biology. I am sorry that we cannot be more positive on this occasion. Please be assured that this editorial decision does not represent a criticism of the quality of your work, nor are we questioning its value to others working in this area. I hope you will consider PLOS Biology for other submissions in the future. Thank you for your support of PLOS and of Open Access publishing.

Sincerely,

Roli Roberts

Roland Roberts, PhD
Senior Editor
PLOS Biology
rroberts@plos.org

Title page

Click here to access/download;Manuscript;Title_Page_PMFY.docx

1. Manuscript Title:

Nash equilibrium of attack and defense behaviors between predators and prey

2. Abbreviated Title:

Nash equilibrium of attack and defense

3. List all Author Names and Affiliations in order as they would appear in the published article:

Hiroyuki Ichijo

Department of Anatomy, Faculty of Medicine, University of Toyama

Research Centre for Idling Brain Science, University of Toyama

Sugitani 2630, Toyama 930-0194, Japan

ORCID ID 0000-0002-9663-9997

E-mail: ichijo@med.u-toyama.ac.jp

Yuichiro Kawamura

School of Medicine, University of Toyama

Sugitani 2630, Toyama 930-0194, Japan

E-mail: s2150029@ems.u-toyama.ac.jp

Tomoya Nakamura

Department of Anatomy, Faculty of Medicine, University of Toyama

Research Centre for Idling Brain Science, University of Toyama

Sugitani 2630, Toyama 930-0194, Japan

ORCID ID 0000-0001-7425-6066

E-mail: ntomoya@med.u-toyama.ac.jp

4. Author Contributions:

H.I. was responsible for conceptualization. Y.K., T.N., and H.I. were responsible for the methodology. Y.K., T.N., and H.I. performed the formal analysis. Y.K., T.N., and H.I. carried out the investigations. H.I. wrote the original draft. Y.K., and T.N. reviewed and edited the report. H.I. and T.N. acquired funding.

5. Correspondence should be addressed to (include email address):

Hiroyuki Ichijo

ORCID ID 0000-0002-9663-9997

Department of Anatomy, Faculty of Medicine, Research Centre for Idling Brain Science, University of Toyama, Sugitani 2630, Toyama 930-0194, Japan

E-mail: ichijo@med.u-toyama.ac.jp

Telephone: +81-76-434-7205

Fax: +81-76-434-5010

6. Number of Figures: 5

7. Number of Tables: 3

8. Supplementary Information:

Number of Supplementary Tables: 4

Number of Supplementary Figures: 15

Number of Supplementary Programs: 5

Number of Supplementary Source Data: 22

9. Number of words for Abstract: 149

10. Number of words for Main text: 4983

11. Conflict of Interest:

The authors declare no competing interests.

Main manuscript Click here to access/download;Manuscript;manuscript_PMFY.pdf

Abstract

Uncovering the functions of neuronal circuits in attack/defense behaviors requires an algorithmic and computational understanding. We modeled and analyzed these behaviors with game theory. A sensory-motor algorithm without directionality was sufficient for predators and prey (agents) to modify their payoffs based on encounter probabilities and generate Nash equilibrium attack/defense behaviors. Moreover, the sensory-motor algorithm was not necessary because a simplified single-element motor algorithm was sufficient to achieve Nash equilibrium, generating a function comparable to these behaviors. Despite their similarity, these algorithms had distinct adaptive significance. The sensory-motor algorithm produced substantially greater prey payoffs. These demonstrated the fundamental mechanisms for yielding attack/defense behaviors; implementing these algorithms computes agents' payoffs and achieves the Nash equilibrium behaviors. These findings suggest that the evolutionary mechanisms underlying attack/defense behaviors are intrinsic to the model environments where agents are subjected to selection. Therefore, the present approach is valuable for an experimental understanding of their evolution.

Introduction

Attacking prey and defending against predators are basic survival behaviors. Attack and defense behaviors are traits in which predators capture prey animals, and prey animals evade predators, respectively. Predators find and attack their prey by chasing (i.e., actively increasing their speed) or ambushing (i.e., stopping their movement, passively waiting, and making a surprise attack) it[1,2]. On the other hand, threat stimuli: such as predator smells, looming shadows, and warning sounds, automatically evoke defense behaviors in prey animals, including escaping (i.e., actively increasing their speed) and freezing (i.e., stopping their movement and passively waiting for the threat to pass)[3–15].

How predators capture prey and how prey evade predators, these challenges in information processing are likely to recur frequently in predator-prey interactions. Neuronal circuits implementing attack and defense behaviors physically embody programs at the algorithmic level, allowing for the efficient computation of solutions to these information-processing problems at the computational level, as described in the three levels postulated by Marr: implementation, algorithm, and computation[8,16]. The fundamental mechanisms of defense behaviors in mice are innate and implemented by genetically hard-wired neuronal circuits[3,17–20]. Recent studies have demonstrated the level of implementation of defense behaviors. However, algorithms and computations underlying attack and defense behaviors have been overlooked.

Therefore, numerically evaluating the modifications caused by the agents' behaviors aids in understanding the algorithms and computations of the behaviors. The mutual influence of attack and defense behaviors can be analyzed using game theory[21–27], which has been described as a noncooperative constant-sum game[28–30]. The solution of the game is defined by the Nash equilibrium, which ensures stable outcomes, in which neither the predators nor the prey increase their payoffs by replacing alternative behaviors[31]. Determining the Nash equilibrium behaviors can elucidate how agents engage in behavioral patterns and achieve stable payoffs[22,28,32,33]. However, determining the Nash equilibrium presents challenges, because it involves several components. For example, multiple defense behavioral processes occur when a prey animal encounters a predator. Individual prey animals

sense multiple stimuli, pay attention to the spatial locations of each stimulus source, select salient stimuli, compare the competing events in the situation, make a decision, drive their motor systems under temporal and spatial regulation, evade danger, and survive[15]. This study addresses this challenge and reveals a game-theoretic solution for the Nash equilibrium in the interaction between attack and defense.

To understand the algorithms and computations underlying attack and defense behaviors, this study does not reproduce the complexity but rather extracts the elements within the complexity and presents them in games between a single predator and a single prey animal, or between multiple predators and prey animals, in an individual-based model (IBM). We modeled them using an algorithm of sensory and motor abilities, where the agents move at a constant speed by default, use the distance from the opponent as the sensory input[34], and change the speed as the motor output, although the agents have no directionality regarding which direction the opponent is or in which direction they move. We demonstrate that this algorithm is sufficient for agents to modify their encounter probabilities. By constructing payoff matrices based on the encounter probabilities, we demonstrate that agents generate a Nash equilibrium[35]. Moreover, we show that the sensory-motor algorithm is not necessary because a simplified single-element motor algorithm is sufficient to achieve the Nash equilibrium. The single-element motor and sensory-motor algorithms represent the minimal and fundamental components for computing the agents' payoffs, establishing a Nash equilibrium and its combinations between the agents under varying conditions, and yielding a function comparable to attack/defense and a function of attack/defense, respectively. Finally, we discuss the evolvability of attack/defense behaviors.

Results

Nash equilibrium for attack and defense in one predator and one prey game

This study defined attack and defense behaviors as traits by which a predator captures prey and a prey animal evades predators, respectively. The attacks and defenses of one predator and one prey species were examined in the grid (Fig. 1A, Supplementary Fig. 1). Grid worlds refer to abstract environments composed of grid-like arrangements of squares, commonly used as simplified models in various fields, such as artificial

intelligence and reinforcement learning[36]. They are called lattice models in the field of ecology[37]. To identify the game-theoretic solution, we defined the behaviors in the grid world, where the agents moved at a constant speed between squares (default speed = 1), sensed the opponent, and changed their speed. The behaviors included decreasing the speed to 0, not changing it to 1, and increasing it to 2. These behaviors corresponded to ambush, no change, and chase for predators, and freeze, no change, and escape for prey. The predators and prey were nicknamed “wolves” (w) and “sheep” (s), respectively. The variables and measurements used in the simulations are listed in Tables 1 and 2, respectively.

The agents had a sensory ability (distance to detect the opponent (D_i) ($i = w$ and s) as the range in which the agents detected the opponent) and motor ability (amount of speed change (S_i) when they detected the opponent); however, the agents had no directionality of which direction the opponent was or which direction they moved. Combinations of D_i and S_i generated the agents' behaviors. We calculated the encounter probabilities (p) by combining these behaviors (Supplementary Program, EncounterProbabilities_gridworld.txt). We assumed here that encountering a predator means predation (i.e., p was equivalent to the probability of predation), while not encountering a predator means evasion (i.e., $1 - p$ was the probability of evading predation). The landscape diagrams of p for the nine combinations of D_i are shown in Fig. 1B. These results show that when employing the sensory-motor algorithm without directionality, combinations of behaviors produce diverse encounter probabilities, generating various payoffs. By comparing the colors in the small squares, it was evident that p depended on the S_i of the agent and the opponent, as well as D_i[29].

We regarded p and $1 - p$ as the predator's and prey's payoffs, respectively, and constructed payoff matrices of a 2-player strategy game (Source Data, PayoffMatrices_grid_world.xlsx, source_data.pdf). This is a noncooperative constant-sum game because the sum of both payoffs is constant ($p + (1 - p) = 1$). To determine the solutions to the game, we calculated the Nash equilibria from the payoff matrices of the predator and prey for D_i combinations. In the Nash equilibrium, the values of S_i are denoted as $\bar{S}_i$. $\bar{S}_i$ was plotted for combinations of D_i at the same coordinates as the landscape (Fig. 1C) (Source Data, NashEq_Si_grid_world.xlsx). For the combinations of D_i, a single $\bar{S}_i$ value was determined (pure strategy) depending on D_i. The effect of D_s

on $\bar{S}_i$ is substantial. For the predator, $\bar{S}_w$ tended to depend on the opponent D_s. When D_w = 1, the predator adopted $\bar{S}_w$ = -1 (ambush) if D_s = 0, and $\bar{S}_w$ = 0 (no change) if D_s = 1. The predator adopted any $\bar{S}_w$ value with no difference in the payoff when D_s = 2. For the prey, $\bar{S}_s$ tended to depend on their own D_s. When D_s = 1, the prey adopted $\bar{S}_s$ = 1 (escape); if D_w = 0 or 1. When D_s = 2, the prey adopted $\bar{S}_s$ = -1 (freeze) if D_w = 0 or 1. The prey adopted any $\bar{S}_s$ value with no difference in payoff if D_w = 2.

When the agents detected their opponents two grids apart ($D_w = D_s = 2$), both agents adopted the three $\bar{S}_i$ values and probabilistically changed their speeds, as indicated by the orange circles. Their diameters were proportional to their occurrence probability ($\bar{o}_i$) (mixed strategy) (Fig. 1Cc). The prey escaped with an occurrence probability of 0.363 ($\bar{S}_{s,1}$= 1, $\bar{o}_{s,1}$ = 0.363), maintained its speed with a probability of 0.499 ($\bar{S}_{s,2}$= 0, $\bar{o}_{s,2}$ = 0.499), and froze with a probability of 0.139 ($\bar{S}_{s,3} = -1$, $\bar{o}_{s,3}$ = 0.139). The predator chased its prey with an occurrence probability of 0.363 ($\bar{S}_{w,1}$= 1, $\bar{o}_{w,1}$ = 0.363), maintained its speed with a probability of 0.499 ($\bar{S}_{w,2}$ = 0, $\bar{o}_{w,2}$ = 0.499), or ambushed its prey with a probability of 0.139 ($\bar{S}_{w,3} = -1$, $\bar{o}_{w,3}$ = 0.139). When the agents did not detect the opponent and did not change their speed ($D_w = D_s = 0$), they did not behave; $\bar{S}_i$ was not determined but took any value (Fig. 1Cg). These results show that different D_i combinations resulted in different values of $\bar{S}_i$ and that the effect of D_s was substantial on $\bar{S}_i$.

Nash equilibrium for attack and defense in multiple predators and prey game

The game in the grid world does not consider the agent densities, costs dependent on sensory and motor traits, or costs dependent on the resulting speed. Therefore, we developed an IBM that considers the effects in which multiple agents move, eat food, reproduce, pay costs, and die. Using a programming language and an integrated development environment called NetLogo[38], we created a simulator and solved a game (Fig. 2A, B) (Supplementary Program, EncounterProbabilities_sensory_motor.nlogo). This approach is called empirical game-theoretic analysis[39]. The agents had a sensory trait (d_i), which caused the sensory ability to detect the opponent regardless of direction within a certain distance range (D_i). D_i is a value obtained by multiplying d_i by a constant coefficient representing the sensitivity of the sensory trait (factor sensitivity, f), that is, $D_i = d_i \times f$. These agents

exhibited motor traits without directionality (S_i). The cost dependent on the sensory and motor traits was the sum of the sensory trait (d_i) and the absolute value of the motor trait (S_i) multiplied by the behavioral cost coefficient (c_b); that is, (d_i + abs(S_i)) × c_b, which corresponds to the cost associated with implementing neuronal circuit mechanisms for the behaviors. The cost dependent on the resulting speed was (1 or 1 + S_i) multiplied by a constant, that is, the movement cost coefficient (c_m). Therefore, different values were subtracted from the energy of the agent at each time point when the agent changed or did not change its speed.

Fig. 2C depicts the landscapes of encounter frequencies (E) with the values of c_b (= 0.02) and the reproductive coefficient of the predators (r = 3.6). The combinations of D_i and S_i produced diverse encounter frequencies. The landscape shifted continuously depending on S_i when D_s = 0 and 1 (Fig. 2Ca, b, e, f, i, j, m, and n). Discontinuous transitions in the encounter frequencies were observed at S_s = 0 when D_s = 2 and 3 (Fig. 2Cc, d, g, h, k, l, o, and p), indicating a marked decrease in the encounter frequencies when the prey changed its speed.

We defined predator payoffs (P_w) as probabilities that predators encountered their prey ($P_w = E/N_w$, where N_w is the number of predators) and prey payoffs (P_s) as probabilities that the prey did not encounter predators ($P_s = 1 - E/(E + N_s)$, where N_s is the number of prey animals). Diverse encounter probabilities were generated. The P_s and P_w landscapes are shown in Fig. 2D, and 2E, respectively. The P_s landscapes were similar to the encounter frequencies in the pattern but with inverted colors. Individual prey obtained greater payoffs when their speed changed (Fig. 2Dc, d, g, h, k, l, o, and p). By contrast, the P_w landscapes shifted gradually and continuously with no discontinuous transitions (Fig. 2E).

We constructed payoff matrices using P_w and P_s for the combinations of D_i (Source Data: PayoffMatrices_r3.2-4.0_c0 ~ 40_run1-6.xlsx), computed $\bar{S}_i$ values (Source Data: NashEq_Si_c0 ~ 40_r3.2-4.0.xlsx), and plotted them on the same coordinates as the payoff landscape (Fig. 2F). The blue circles represent pure strategies with a probability of 1 ($\bar{o}_i = 1$). Depending on D_i, the $\bar{S}_i$ value was determined. The effect of D_s on $\bar{S}_i$ is noticeable. For the predator, $\bar{S}_w$ tended to depend on the opponent D_s. When $D_s = 0$, the predator adopted $\bar{S}_w > 0$ (chase) (Fig. 2Fa, e, i). When $D_s \neq 0$, the

predator adopted $\bar{S}_w < 0$ (ambush) (Fig. 2Fb, c, d, f). For the prey, $\bar{S}_s$ tended to depend on D_s. When $D_s \neq 0$, the prey adopted $\bar{S}_s > 0$ (escape) (Fig. 2Fb, c, d, f, h, j, k, l, n, o, p). When the predator did not detect prey or detected prey nearby, or the prey detected predators from afar ($D_w = 0, 1, 2$, and $D_s = 3$, or $D_w = 0, 1$, and $D_s = 1, 2$), the predator was extinct; therefore, the $\bar{S}_w$ was not determined and assumed any value (Fig. 2Fh, j, k, l, n, o, and p). When the prey did not detect the predator or change its speed ($D_w = 1, 2, 3$, and $D_s = 0$), the prey adopted $\bar{S}_s = -1, -0.8, 0.8$, or 1, where high costs were required for the motor traits (Fig. 2Fa, e, and i).

When both agents detected their opponents from afar ($D_w = D_s = 2$), sets of probabilistic behavior switching were the Nash equilibrium (Fig. 2Fg, orange circles). The predators chased their prey with an occurrence probability of 0.462 ($\bar{S}_{w,1} = 1$, $\bar{o}_{w,1} = 0.462$) and ambushed their prey with a probability of 0.538 ($\bar{S}_{w,2} = -1$ $\bar{o}_{w,2} = 0.538$). The prey escaped with a probability of 0.605 ($\bar{S}_{s,1} = 1$, $\bar{o}_{s,1} = 0.605$) and froze with a probability of 0.395 ($\bar{S}_{s,2} = -1$, $\bar{o}_{s,2} = 0.395$) (Table 3). This resulted in the four types of behavioral pairs: chase×escape (1), ambush×escape (2), ambush×freeze (3), and chase×freeze (4), located at the corners of the diagram.

When the agents did not detect the opponent ($D_w = D_s = 0$), $\bar{S}_s$ values were determined; although they did not change their speeds, they were located at the corners of the diagram with large absolute values of motor traits (Fig. 2Fm). This was likely because of the high cost of the motor traits, which increased P_s (Fig. 2Dm) by reducing E (Fig. 2Cm). These findings confirmed the results in the grid world, where different D_i combinations resulted in different $\bar{S}_i$ values, and the effect of D_s on $\bar{S}_i$ was substantial. Furthermore, the results of the Nash equilibria for all c_b (0, 0.001, 0.02, 0.04) and r (3.2, 3.6, 4.0) are shown in Supplementary Figs. 2, 3, 4, and 5, demonstrating that distinct Nash equilibrium combinations were established under varying conditions.

The Nash equilibrium behavioral payoffs in attack and defense

The game between multiple predators and prey was a constant-sum game only when the agents did not encounter each other or the numbers of both agents were the same (when $E = 0$ or $N_w = N_s$, $P_s + P_w = 1 - E/(E + N_s) + E/N_w = 1$). However, in general, the game between multiple predators and prey was typically a non-constant-sum game when they did encounter each other and their numbers were different (when $E \neq 0$ and $N_w \neq N_s$, P_s

$+ P_w = 1 - E/(E + N_s) + E/N_w \neq 1$). Furthermore, it was predicted that when N_s was greater than N_w, the sum of the agents' payoffs was greater than one ($P_s + P_w > 1$) because of $E/N_w - E/(E + N_s) > 0$.

To examine effects of the Nash equilibrium behaviors on agents, we assigned agent sets of D_i and $\bar{S}_i$ (Source Data, cb_r_Di_oi1_Si1_Si2_sensory_motor.xlsx) to perform the Nash equilibrium behaviors (Supplementary program, NashEq_ActualMesurement_sensory_motor.nlogo) and measured E, N_i, and the Nash equilibrium payoffs ($\bar{P}_i$) (Source Data, NashEq_ActualMesurement_sensory_motor.csv). In the IBM, N_s was usually larger than N_w (Fig. 3Aa); a line ($N_s = N_w$) was drawn on the diagram, and most points were distributed in the lower-right area ($N_s > N_w$). As E increased, N_s decreased (Fig. 3Ab) and $\bar{P}_s$ ($= 1 - E/(E + N_s)$) became smaller (Fig. 3Ac). However, because N_w was proportional to E (Fig. 3Ad), $\bar{P}_w$ ($= E/N_w$) did not change markedly as E increased (Fig. 3Ae). We scatter-plotted all the data of $\bar{P}_i$ (Fig. 3Af) for all c_b and r values for $\bar{P}_s$ and $\bar{P}_w$ on the x and y axes, respectively. A line ($\bar{P}_s + \bar{P}_w = 1$) was drawn on the diagrams. Most of the points were in the upper-right area ($\bar{P}_s + \bar{P}_w > 1$), indicating that the sum of the agents' payoffs is greater than 1, as predicted.

To examine the effects of D_s, we compared $\bar{P}_i$ between different values of D_s, with a constant value for D_w. As D_s increased, E decreased (the dark blue points shifted to the left side of Fig. 3B), resulting in increasing $\bar{P}_s$ (the dark blue points shifted to the upper side of Fig. 3B). This indicated that the prey's Nash equilibrium defense increased payoffs by reducing their encounters. To examine the effects of D_w, we compared the $\bar{P}_i$ between different D_w, holding D_s constant. The effects of D_w were dependent on D_s. When $D_s = 0$, as D_w increased, E did not change markedly; however, N_w decreased (the dark brown points shifted to the lower side of Fig. 3Ca), resulting in increasing $\bar{P}_w$ ($= E/N_w$) (the dark brown points shifted to the upper side of Fig. 3Cb). By reducing N_w, N_s increased (the dark brown points shifted to the right side of Fig. 3Cc), causing the increase of $\bar{P}_s$ ($= 1 - E/(E+N_s)$) (the dark brown points shifted to the right side of Fig. 3Cd). Consequently, both $\bar{P}_w$ and $\bar{P}_s$ increased as D_w increased (the dark brown points shifted to the upper-right side of Fig. 3Cd). Therefore, the predators' Nash equilibrium attack increased payoffs for both predators and prey when the prey did not

defend itself. These findings were independent of c_b and r. Plots of the E-N_i, E-$\bar{P}_i$, N_i, and $\bar{P}_i$ for different D_i values are shown in Supplementary Figs. 6 and 7. For comparison, all plots of the Nash equilibrium payoffs in the grid world are shown in Supplementary Fig. 8, which illustrates a noncooperative, constant-sum game.

Effects of Nash equilibrium behavioral switching.

The Nash equilibrium behavior was either pure or mixed when the combinations of D_s and D_w were (1, 1), (1, 2), (1, 3), (2, 2), (2, 3), (3, 1), (3, 2), and (3, 3) for all c_b and r. The mixed strategy resulted in probabilistic switching with increasing and decreasing speeds, predators chasing and ambushing their prey, and prey animals escaping and freezing (Fig. 2Fg, Supplementary Figs. 2Df, 2Ff, 2Fg, 3Bg, 3Dg, 3Ff, 3Fg, 3Fj, 3Fl, 4Dg, and 4Fg). To examine the effects of behavioral switching, we compared E between behavioral switching and non-switching. Behavioral switching provided a large E (E > 4) (Fig. 3Da), which was observed specifically for a small value of D_s (D_s = 1) (Fig. 3Db), but did not depend on D_w (Fig. 3Dc). We scatter-plotted the $\bar{o}_i$, N_i, and $\bar{P}_i$ of behavioral switching with $E > 4$ (dark orange), behavioral switching with $E \leq 4$ (orange), and non-switching (blue). $\bar{o}_i$ were spread over a broad range in the scatter plot (Fig. 3Dd), indicating that behavioral switching was heterogeneous with varying combinations of the $\bar{o}_i$ between predator and prey. Among them, $\bar{o}_i$ with $E > 4$ were mainly distributed in the center, indicating that the switching probabilities of the two behaviors were relatively close. This behavioral switching with $E > 4$ increased N_w and decreased N_s (Fig. 3De), decreasing $\bar{P}_s$ with slight changes in $\bar{P}_w$ (Fig. 3Df). Therefore, behavioral switching with $E > 4$ is advantageous for predators.

Moreover, Nash equilibrium behavior switching frequently occurred for small values of c_b (Fig. 3Dg). In addition, we observed that one of the agents performed Nash equilibrium probabilistic switching, that is, partial behavioral switching (Supplementary Fig. 9).

Nash equilibrium speed changes in the single-element motor algorithm.

To ascertain whether the sensory-motor algorithm is necessary for agents to achieve the Nash equilibrium, we formulated a simplified single-element motor algorithm in which agents change their speed stochastically without sensory information. We defined stochastic speed change as the probability of speed change (K_i) and S_i (Fig. 4A).

Examining the encounter probability with a program created by NetLogo (Supplementary Program, EncounterProbabilities_single_element_motor.nlogo), we showed that combinations of K_i and S_i produced diverse encounter probabilities (Fig. 4B), generated agent payoffs (Fig. 4C, D), and achieved Nash equilibrium (Fig. 4E) (c_b = 0.02 and r = 3.6). For the predators, either positive or negative $\bar{S}_w$ settled irrespective of K_w but depended on K_s, which indicated the increases in the predators' speed (such as while chasing prey) when K_s = 0, 0.1, or 0.2 (Fig. 4Ea, b, c, e, f, g, i, j, and k), and the decrease (such as while ambushing prey) when K_s = 0.3 (Fig. 4Ed, h). For the prey, positive $\bar{S}_s$ settled irrespective of K_i, increasing the speed of the prey (such as while escaping) (Fig. 4Eb, c, d, f, g, h, j, k, n, and o). When K_w = 0, 0.1, and K_s = 0.3, positive $\bar{S}_s$ settled (such as while escaping), causing predator extinction; therefore, $\bar{S}_w$ was not determined and did not have a single value (Fig. 4El, p). When $K_w = K_s = 0$, $\bar{S}_i$ had large absolute values of motor traits, as in the sensory-motor algorithm (Fig. 4Em). Therefore, the sensory-motor algorithm is not necessary, but the single-element motor algorithm is sufficient for the agents to achieve a Nash equilibrium speed change. In addition, different K_i combinations resulted in different values of $\bar{S}_i$, and the effect of K_s on $\bar{S}_w$ was substantial. All landscapes of the payoff difference between the prey and predator and the Nash equilibrium with the single-element motor algorithm for all c_b and r are presented in Supplementary Figs. 10, 11, 12, and 13, showing that distinct Nash equilibrium combinations were established under varying conditions.

To examine the effects of the Nash equilibrium speed change on the agents, we assigned agent sets of K_i and $\bar{S}_i$ (Source Data, cb_r_Ki_oi1_Si1_Si2_single_element_motor.xlsx) to perform the Nash equilibrium speed change (Supplementary program, NashEq_ActualMesurement_single_element_motor.nlogo), measuring E, N_i, and $\bar{P}_i$ (Source Data, NashEq_ActualMesurement_single_element_motor.csv). N_s was usually larger than N_w (Fig. 4Fa). As E increased, N_s remained constant (Fig. 4Fb), and $\bar{P}_s$ decreased (Fig. 4Fc). However, as N_w was proportional to E (Fig. 4Fd), $\bar{P}_w$ remained relatively constant (Fig. 4Fe). We scatter-plotted $\bar{P}_s$ and $\bar{P}_w$ (Fig. 4Ff) on the x and y axes, respectively. Most of the plots were in the upper-right area ($\bar{P}_s + \bar{P}_w > 1$). To examine the effects of K_s, we compared $\bar{P}_i$ between different K_s, holding K_w constant.

As K_s increased, E decreased, increasing $\bar{P}_s$ (the dark blue points shifted to the upper-left side of Fig. 4G). To examine the effects of K_w, we compared $\bar{P}_i$ between different K_w, holding K_s constant. The effect of K_w depended on K_s. When $K_s = 0$, as K_w increased, E did not change considerably but N_w decreased (the dark brown points shifted to the lower side of Fig. 4Ha), increasing $\bar{P}_w$ (the dark brown points shifted to the upper side of Fig. 4Hb). By reducing N_w, N_s increased (the dark brown points shifted to the right side of Fig. 4Hc), increasing $\bar{P}_s$ (the dark brown points shifted to the right side of Fig. 4Hd). Consequently, both $\bar{P}_w$ and $\bar{P}_s$ increased as K_w increased (the dark brown points shifted to the upper-right side of Fig. 4Hd). Therefore, the prey's Nash equilibrium speed changes increased their payoffs by reducing encounters, and the predators' Nash equilibrium speed changes increased payoffs for both the predators and prey when the prey did not change speed. These results are similar to those obtained using the sensory-motor algorithm. These findings were independent of c_b and r. Plots of the E-N_i, E-$\bar{P}_i$, N_i, and $\bar{P}_i$ for different K_i are shown in Supplementary Figs. 14 and 15.

Despite this similarity, we found substantial differences in the E, N_i, and $\bar{P}_i$ values of the single-element motor and sensory-motor algorithms. Compared with the single-element motor algorithm, the sensory-motor algorithm decreased E (Fig. 5A) and N_w (Fig. 5B), while increasing N_s (Fig. 5C) and $\bar{P}_s$ (Fig. 5E). Additionally, the sensory-motor algorithm resulted in numerous instances of $\bar{P}_w = 0$, indicating predator extinctions (Fig. 5D). These results indicate that the sensory-motor algorithm is more adaptive for the prey.

Discussion

In this study, we established a method for analyzing the interaction between predators and prey using game theory and demonstrated that the implementation of a single-element motor or sensory-motor algorithm computes agents' payoffs based on encounter probabilities and enables agents to achieve a Nash equilibrium. When the game was constructed between single agents in the sensory-motor algorithm, it was constant-sum and noncooperative (Supplementary Fig. 8), as has been considered in previous studies[28,30,40]. However, when we constructed a game between multiple agents in

the sensory-motor algorithm, the game was a non-constant sum, and the sum of $\bar{P}_i$ was greater than 1 when N_s was greater than N_w. This predicts that both agents can obtain greater payoffs depending on their conditions.

As predicted above, under an attack without defense, both agents obtained a greater $\bar{P}_i$. In behavioral combinations in which both agents' payoffs increase together, both agents have an incentive to choose those behaviors. Therefore, predators have an incentive to attack, and prey have an incentive to receive an attack. Moreover, both agents' $\bar{P}_i$ values increased when the attack opportunities increased as D_w increased. When the prey defended against a predator's attack, $\bar{P}_s$ increased; thus, the prey have an incentive to defend itself. These findings indicate the initiation of the attack and defense behaviors for each of the agents (Fig. 3B, C, Supplementary Figs. 6 and 7). When engaging in attack and defense behaviors, predators can approach their $\bar{P}_w$ empirically or prey with $\bar{P}_s$ have a greater chance of survival. Various Nash equilibrium behaviors appeared, depending on the combinations of D_i; for example, predators chasing their prey (Fig. 2Fa, e, and i), predators ambushing their prey and prey escaping a predator (Fig. 2Fb, c, d, and f), or prey escaping a predator (Fig. 2Fh, j, k, l, n, o, and p), as in wild.

In particular, when the agents detected opponents from afar, switching the two behaviors probabilistically became a Nash equilibrium (Fig. 2Fg and Supplementary Figs. 2–4, 9). The predators chased or ambushed, and the prey escaped or froze in different combinations of the $\bar{o}_i$ values under combinations of D_i, c_b, and r, resulting in a Nash equilibrium (Fig. 3Dd). In this Nash equilibrium behavioral switching, the prey obtained a lower $\bar{P}_s$. These results suggest that the implementation of an algorithm that controls $\bar{o}_i$ in neuronal circuits is reasonable, although it has not been considered previously[41,42]. The increase and decrease in their $\bar{P}_i$, through attacking without defense and behavioral switching, respectively, were owing to alterations in E and N_i under the effects of the interaction between multiple agents. All findings illustrate that the agents generated various combinations of attack and defense behaviors in the Nash equilibrium under different conditions and interactions.

Moreover, we demonstrated that the sensory-motor algorithm was not necessary because the single-element motor algorithm was sufficient to modify P_i and

achieve a Nash equilibrium, generating a function comparable to attack/defense behaviors (Fig. 4). Any variation in the agent's movement mechanism, for example, in neuronal circuitry[43–45] or even in simpler devices, can cause stochastic fluctuations in speed, producing variances in speed change and its probability. Thus, the neuronal circuits for movement can implement a single-element motor algorithm through intrinsic variations of their own. These findings highlight the fundamental mechanism driving attack/defense behaviors in the sensory-motor algorithm, as well as the minimal mechanism responsible for comparable behaviors in the single-element motor algorithm.

Considering the innate nature of defense behaviors, we discuss a potential mechanism through which predators and prey coevolve their attack and defense behaviors in complex environments[46]. Neuronal circuits that implement the sensory-motor algorithms enable agents to derive payoffs as computational outcomes. Because attack and defense behaviors are directly subject to selection through predation, agents with neural circuits that can perform these behaviors to obtain $\bar{P}_i$ are selected frequently. Therefore, when agents with the sensory-motor algorithm are under selection, an evolutionary mechanism may inherently exist within the environment, potentially leading to the automatic selection of neuronal circuits that derive $\bar{P}_i$. The predators can empirically adjust, seek, and reach $\bar{P}_w$ in the payoff landscape through learning. By contrast, prey can have a greater chance of survival with $\bar{P}_s$ but face the risk of dying otherwise; thus, there is no opportunity for them to learn from defense failures[47]. These suggest that attack and defense behaviors can be implemented in neuronal circuits as the acquired and innate behaviors of predators and prey, respectively. Consequently, the Nash equilibrium attack/defense behaviors will coevolve; moreover, their diverse behaviors, patterns of behavioral combinations, and interactions, including increasing and reducing their $\bar{P}_i$ can coevolve. The present findings may explain the evolvability of attack/defense behaviors.

Furthermore, the implementation in the single-element motor algorithm is also directly subject to selection. This suggests that combinations of Nash equilibrium speed changes may have coevolved as a prototype, generating characteristics similar to those of the sensory-motor algorithm. Despite these similarities, the functions of these

algorithms were distinct, indicating a difference in adaptive significance. The sensory-motor algorithm yielded a greater $\bar{P}_s$ and was adaptive for the prey (Fig. 5). Moreover, among the behaviors produced by the sensory-motor algorithm, behavioral switching was specifically advantageous to predators (Fig. 3D). The functional and adaptive differences between the single-element motor and sensory-motor algorithms, as well as between non-switching and switching behaviors in the sensory-motor algorithm, raise questions about their phylogenetic relationships.

This study had some limitations. First, we do not understand algorithmic effects of the sensory element or its concatenation with the motor elements. A numerical evaluation will help address the evolvability of both the single-element motor algorithm and the sensory-motor algorithm, as well as their interrelationship. Second, we do not know in the sensory-motor algorithm, the mechanism by which Nash equilibrium behavior switching frequently occurs for small values of c_b (Fig. 3Dg). Third, we do not know how different Nash equilibrium behaviors exist in a sensory-motor algorithm with or without directionality. Finally, we did not account for population dynamics because we used the average number of individuals in this study; further research may require ecological approaches and methodologies.

Methods

Formulation of attack and defense behaviors with a sensory-motor algorithm

In this study, we defined attack and defense behaviors as traits by which a predator captures prey and a prey evades predators, respectively. Furthermore, we defined chase-ambush as the attack behavior of predators; chase is used to detect prey and increase speed, whereas ambush is used to detect prey and reduce speed[48,49]. We defined escape-freeze as the defense behavior of the prey; escape is used to detect predators and increase speed, whereas freeze is used to detect predators and reduce speed[15,18]. The IBMs consisted of two types of agents: predators and prey (Figs. 1A and 2A). We modeled the attack and defense behaviors with an algorithm using the distance from the opposing agent as sensory input[34] to change the speed as the motor output, but did not include sensory and motor directionalities. Agents have no directionality in the direction in which the opponent is or the direction in which they move. The formulation was based on Edelaar's method for representing phenotypic plasticity[50]. The agents had a sensory ability, a distance to detect the opponent (D_i) ($i = w$ and s, as predator and prey, respectively). D_i was the sensory trait (d_i) multiplied by a constant coefficient of the sensory trait (factor sensitivity, f), that is, $D_i = d_i \times f$, where $f = 10$. The agents had a motor ability, an amount of speed change (S_i) when they detected their opponent. The predator had the sensory ability D_w ($0 \leq D_w$) and the motor ability S_w ($-1 \leq S_w \leq 1$) for its behavior, where $_w$ indicates the predator (wolf). The predator moved in two-dimensional space at a speed of 1 and changed its speed to $1 + S_w$ when it found prey within a radius of D_w. When S_w was greater than 0 ($0 < S_w$), the predator's speed increased (chase). When S_w was less than 0 ($S_w < 0$), the predator's speed decreased (ambush). Without a prey, it moved at a speed of 1. Similarly, the prey had D_s ($0 \leq D_s$) and S_s ($-1 \leq S_s \leq 1$), where $_s$ indicates the prey (sheep). The prey moving at speed 1 changed its speed to $1 + S_s$ when it detected a predator within radius D_s. At $0 < S_s$, the prey's speed increased (escape). At $S_s < 0$, the prey's speed decreased (freeze). Without a predator, it moved at a speed of 1. In addition to excluding sensory and motor directionalities, we did not consider the distance in the radius (far or close) or the sensory modality detecting the opponent[15].

Landscape diagrams of encounter probabilities for one predator and one prey with

the sensory-motor algorithm

We created a grid world model to investigate the attack and defense behaviors of a predator and prey in a discrete space (Fig. 1A). One predator and one prey were located on a two-dimensional grid (Fig. 1A-t1) and moved one grid per time step (Fig. 1A-t2). An agent detected the opponent in the D_i range (Moore neighborhood) and changed its speed to $1 + S_i$ (Fig. 1Aa-t2, b-t2, c-t2, and d-t2). D_i assumed the values of 2, 1, or 0 (an agent detected the opponent two grids apart (= 2), detected the opponent in adjacent grids (= 1), or did not detect the opponent (= 0)). S_i assumed the values 1, 0, or −1 (an agent changed its speed to 2 (= 1 + 1), left the speed unchanged at 1 (= 1 + 0), or changed the speed to 0 (= 1 − 1)). When the predator and prey occupied the same grid, the prey was eaten; thus, the prey died (Fig. 1Aa-t3, b-t3, and d-t3). We examined the encounters between agents in a single time step. Because the maximum value of D_i was 2, we prepared 5 × 5 grids in the x–y plane with coordinates (x, y) = (0 ~ 4, 0 ~ 4). To set the relative positions of the agents, a predator was placed at coordinate (2, 2), and the prey was placed at arbitrary coordinates. When $D_i = 2$, the agents recognized the opponents at the outer edge, and the combinations in which they were adjacent to each other were excluded. For all combinations of agent coordinates, the next positions were determined based on D_i and S_i. We wrote a script to calculate the encounter probability (p) from the number of encounters and the total number of combinations using R[51] (Supplementary program, EncounterProbabilities_gridworld.txt). For combinations of D_i, p was represented by S_s and S_w on the x and y axes, respectively (Fig. 1B). The grid world does not include the effects of agent density or the costs associated with their behavior.

Landscape diagrams of encounter probabilities for multiple predators and prey with the sensory-motor algorithm

We constructed a model in which multiple agents (predators and prey) moved and interacted in a continuous two-dimensional space by modifying the sheep–wolf predation model in the NetLogo model library[38] (Fig. 2A, Supplementary program, EncounterProbabilities_sensory_motor.nlogo). The predators moved, gained energy by eating prey when they encountered prey in the same patch, reproduced, lost energy as they paid costs, reproduced, and died when their energy reached zero. The prey moved,

gained energy by eating grass, reproduced, lost energy as they paid costs, reproduced, and died when their energy reached zero or when they encountered a predator in the same patch (Fig. 2B). We considered three types of costs: basal metabolism, cost dependent on sensory and motor traits, and cost dependent on the resulting speed. Basal metabolism remained constant (c_e). The cost dependent on sensory and motor traits was the sum of the sensory trait (d_i) and the absolute value of the motor trait (S_i) multiplied by the behavioral cost coefficient (c_b); thus, it was calculated as follows.

$$(d_i + \mathrm{abs}(S_i)) \times c_b$$

This corresponded to the cost associated with implementing neuronal circuit mechanisms for these behaviors. The cost of the resulting speed was calculated by multiplying the speed (1 or 1 + S_i) by a constant, the movement cost coefficient (c_m), as follows.

$$(1 \text{ or } 1 + S_i) \times c_m$$

Thus, in the simulation, different cost values were subtracted from the energy of the agent at each time point when the agent changed or did not change its speed. When the speed of the agent changed to 1 + S_i, the following value was subtracted.

$$c_e + (d_i + \mathrm{abs}(S_i)) \times c_b + (1 + S_i) \times c_m$$

While the following value was subtracted when the agent did not change its speed.

$$c_e + (d_i + \mathrm{abs}(S_i)) \times c_b + 1 \times c_m$$

Predators and prey were predetermined to perform a single behavior in the IBMs. The sensory ability (D_i) was d_i multiplied by a constant, the coefficient of the sensory trait (factor sensitivity, f), that is, $D_i = d_i \times f$, where $f = 10$; D_i had 4 different values (0, 1, 2, and 3). The motor ability (S_i) was equal to S_i, which considered 11 different values ranging from −1 to 1 in intervals of 0.2. The combinations of D_i and S_i

generated 44 different sets of behaviors. The following parameters were set as constants: energy gained by a predator eating prey, energy gained by prey from eating grass, reproduction coefficient of prey, time for grass to grow, c_e, c_m, and f. The following variables were used: d_i, D_i, S_i, c_b, and r. Values of the constants and variables used in the simulations with the sensory-motor algorithm are listed in Supplementary Tables 1 and 2, respectively.

Simulations were performed for all predator-prey behavior pairs using nlrx[52], which controls NetLogo in R. When each simulation was run from the beginning up to 10,000-time steps and the interaction between predators and prey reached a steady state, the following data were collected from 10,000 to 11,000-time steps: time step, number of encounters at each time point (encounter frequency, E), number of wolves (N_w), and number of sheep (N_s). The encounter probabilities of the agents (encounter probability of wolves: $p_w = E/N_w$, encounter probability of sheep: $p_s = E/(E + N_s)$), payoffs of the agents (payoff of wolves: $P_w = p_w$, payoff of sheep: $P_s = 1 - p_s$), and differences in payoffs ($P_{diff} = P_s - P_w$) were calculated for each time step. The measured values fluctuated periodically, and the number of cycles was less than 1000 time steps. The averages of the measured values from 10,000 to 11,000 time steps were represented. We examined the effects of c_b (0.001, 0.02, and 0.04) and r (1.4, 1.8, 2.8, 3.2, 3.6, and 4.0) on E, P_w, P_s, and P_{diff}. For each combination of D_i, 121 simulations were run, in which each engaged in 11 different behaviors (11 S_w and 11 S_s). E, P_w, P_s, and P_{diff} were placed on the diagrams, with S_s and S_w on the x and y axes, respectively. Sixteen landscape diagrams were depicted for the combinations of D_i. The experiments were repeated six times.

Nash equilibrium behaviors in the sensory-motor algorithm

We studied the functions of these behaviors using game theory[28]. Attack and defense behaviors were considered as predator and prey strategies, respectively, and consisted of a strategic game between the two players[21]. In the grid world, encounter probability (p) was the payoff for the predator and $1 - p$ was the payoff for the prey. The bimatrices with p and $1 - p$ were the payoff matrices of constant-sum games. The S values of the Nash equilibrium ($\bar{S}_i$) were calculated under combinations of D_i by using Game Theory Explorer (GTE) (https://cgi.csc.liv.ac.uk/~rahul/bimatrix_solver/)[31,53]. A script was written in Python to automate the calculations using GTE. $\bar{S}_i$ were shown at the same

coordinates as those in the payoff landscape diagrams.

In the NetLogo world, although many agents were included, the game was considered a 2-player noncooperative strategy game because an agent sought payoffs by interacting with the opponent at each time point. For each combination of D_i, the bimatrix of P_i was assumed to be the payoff bimatrix of a game in which either agent had 11 different strategies against the opponent. This was regarded as a game between the predator and prey[54,55]. The calculations of $\bar{S}_i$ using the GTE[53] were automated using a script written in Python. $\bar{S}_i$ was shown for the combinations of D_i at the same coordinates as the payoff landscape diagrams.

To examine effects of the Nash equilibrium behaviors on agents, we assigned agent sets of D_i and $\bar{S}_i$ (Source Data, cb_r_Di_oi1_Si1_Si2_sensory_motor.xlsx) to perform the Nash equilibrium behaviors (Supplementary program, NashEq_ActualMesurement_sensory_motor.nlogo) and measured E, N_i, and Nash equilibrium payoffs ($\bar{P}_i$) (Source Data, NashEq_ActualMesurement_sensory_motor.csv). The experiment was repeated ten times. We examined the effects of Nash equilibrium behaviors in the sensory-motor algorithm on E, N_i, $\bar{P}_i$, switching probability, and percentages of behavioral switching by drawing scatter, violin, and bar plots.

In this study, we did not use statistical significance tests on p-values to interpret the simulation model results. Statistical methods are considered valuable for data with limited sample sizes, but not for simulation data with unlimited sample sizes[56].

Formulation of agents' speed changes with a single-element motor algorithm and Nash equilibrium in game theory.

The agents' movements were formulated using a single-element motor algorithm. The agents set the probabilities of speed change (K_i) and S_i for their stochastic speed changes (Fig. 4A). The predator moving with speed 1 changed its speed to $1 + S_w$ ($-1 \leq S_w \leq 1$) with the probability K_w ($0 \leq K_w \leq 1$). When S_w was greater than 0 ($0 < S_w$), the speed of the predator increased, corresponding to that of the predators' chasing. When S_w was less than 0 ($S_w < 0$), the speed decreased, corresponding to that of the predators' ambushing. With a probability of $1 - K_w$, the predator moved at a speed of 1. The prey moving with speed 1 changed its speed to $1 + S_s$ ($-1 \leq S_s \leq 1$) with the probability K_s (0

$\leq K_s \leq 1$). When S_s was greater than 0 ($0 < S_s$), the prey speed increased, corresponding to that of the prey's escaping. When S_s was less than 0 ($S_s < 0$), the speed decreased, corresponding to that of the prey's freezing. With a probability of $1 - K_s$, the prey moved at a speed of 1.

We constructed a model with a single-element motor algorithm in the IBM using NetLogo (Supplementary program: EncounterProbabilities_single_element_motor.nlogo). The life history of the agents was the same as that in the sensory-motor algorithm model, consisting of movement, feeding, energy gain or loss, reproduction, and death. The "costs" comprised basal metabolism, the cost of probability and motor traits, and the cost of the resulting speed. Basal metabolism and the cost of the resulting speed were the same as those in the sensory-motor algorithm model. The cost of the probability and motor traits was defined as the sum of K_i and the absolute value of the motor trait (abs(S_i)) multiplied by c_b, which corresponds to the cost associated with implementing neuronal circuit mechanisms for their stochastic speed change, as follows:

$$(K_i + \mathrm{abs}(S_i)) \times c_b$$

The following value was subtracted when the agent changed its speed to $1 + Si$.

$$c_e + (K_i + \mathrm{abs}(S_i)) \times c_b + (1 + S_i) \times c_m$$

Conversely, the following value was subtracted when the agent did not change its speed.

$$c_e + (K_i + \mathrm{abs}(S_i)) \times c_b + 1 \times c_m$$

The probability of speed change (K_i) had four different values (0, 0.1, 0.2, and 0.3). Except for K_i, the other parameters (values of the constants and variables) were the same as those of the sensory-motor algorithm model. The values of the constants and variables used in the simulations with the single-element motor algorithm are listed in Supplementary Tables 3 and 4, respectively. In addition, the measurements and data

collection were the same as those for the sensory-motor algorithm model. This experiment was repeated 6 times. The supplementary source data show the payoff matrices for all the experiments (PayoffMatrices_single_element_motor_r3.2-4.0_c0 ~ 40_run1-6.xlsx).

The Nash equilibrium speed changes were computed in the same manner as those in the sensory-motor algorithm using the GTE with the script written in Python. The results for all the experiments are shown in the Supplementary Source Data (single_element_motor_NashEq_Si_c0 ~ 40_wr3.2-4.0.xlsx). The results of the single-element motor algorithm model were depicted in the same manner as those of the sensory-motor algorithm model; E, P_i, and $\bar{S}_i$ were shown in the landscape diagrams.

To examine the effects of the Nash equilibrium speed changes on agents, we assigned agent sets of K_i and $\bar{S}_i$ (Source Data, cb_r_Ki_oi1_Si1_Si2_single_element_motor.xlsx) to perform the Nash equilibrium speed changes (Supplementary program, NashEq_ActualMesurement_single_element_motor.nlogo) and measured E, N_i, and $\bar{P}_i$ (Source Data, NashEq_ActualMesurement_single_element_motor.csv). The experiment was repeated ten times. We examined the effects of the Nash equilibrium speed changes on E, N_i, and $\bar{P}_i$ in the single-element motor algorithm model by drawing scatter plots. Furthermore, we compared the effects of the single-element motor and the sensory-motor algorithms on E, N_i, and $\bar{P}_i$ by drawing histograms.

Acknowledgements

We thank Dr. Masato Yamamichi for discussion and critical reading of the manuscript, Profs. Hiroshi Nishimaru and Rahul Savani for critical reading of the manuscript.

References

1. Moyer K. Kinds of aggression and their physiological basis. Communications in Behavioral Biology. 1968;2: 65–87.
2. Rossier D, Franca V La, Salemi T, Natale S, Gross CT. A neural circuit for competing approach and defense underlying prey capture. Proc Natl Acad Sci U S A. 2021;118: e2013411118. doi:10.1073/pnas.2013411118
3. Yilmaz M, Meister M. Rapid innate defensive responses of mice to looming visual stimuli. Current Biology. 2013;23: 2011–2015. doi:10.1016/j.cub.2013.08.015
4. Wei P, Liu N, Zhang Z, Liu X, Tang Y, He X, et al. Processing of visually evoked innate fear by a non-canonical thalamic pathway. Nat Commun. 2015;6: 6756. doi:10.1038/ncomms7756
5. De Franceschi G, Vivattanasarn T, Saleem AB, Solomon SG. Vision Guides Selection of Freeze or Flight Defense Strategies in Mice. Current Biology. 2016;26: 2150–2154. doi:10.1016/j.cub.2016.06.006
6. Tovote P, Esposito MS, Botta P, Chaudun F, Fadok JP, Markovic M, et al. Midbrain circuits for defensive behaviour. Nature. 2016;534: 206–212. doi:10.1038/nature17996
7. Vale R, Evans DA, Branco T. Rapid Spatial Learning Controls Instinctive Defensive Behavior in Mice. Current Biology. 2017;27: 1342–1349. doi:10.1016/j.cub.2017.03.031
8. Evans DA, Stempel AV, Vale R, Ruehle S, Lefler Y, Branco T. A synaptic threshold mechanism for computing escape decisions. Nature. 2018;558: 590–594. doi:10.1038/s41586-018-0244-6
9. Shang C, Chen Z, Liu A, Li Y, Zhang J, Qu B, et al. Divergent midbrain circuits orchestrate escape and freezing responses to looming stimuli in mice. Nat Commun. 2018;9: 1232. doi:10.1038/s41467-018-03580-7
10. Zacarias R, Namiki S, Card GM, Vasconcelos ML, Moita MA. Speed dependent descending control of freezing behavior in Drosophila melanogaster. Nat Commun. 2018;9: 3697. doi:10.1038/s41467-018-05875-1
11. Lee KH, Tran A, Turan Z, Meister M. The sifting of visual information in the superior colliculus. Elife. 2020;9: e50678. doi:10.7554/eLife.50678
12. Dielenberg RA, McGregor IS. Defensive behavior in rats towards predatory odors: A review. Neurosci Biobehav Rev. 2001;25: 597–609. doi:10.1016/S0149-7634(01)00044-6
13. Domenici P, Blagburn JM, Bacon JP. Animal escapology I: Theoretical issues and emerging trends in escape trajectories. Journal of Experimental Biology. 2011;214: 2463–2473. doi:10.1242/jeb.029652
14. Tovote P, Fadok JP, Lüthi A. Neuronal circuits for fear and anxiety. Nat Rev Neurosci. 2015;16: 317–331. doi:10.1038/nrn3945
15. Branco T, Redgrave P. The Neural Basis of Escape Behavior in Vertebrates. Annu Rev Neurosci. 2020;43: 417–439. doi:10.1146/annurev-neuro-100219-122527
16. Marr D. Vision: a computational investigation into the human representation and processing of visual information. Vision: a computational investigation into the human representation and processing of visual information. 1982. doi:10.1016/0022-2496(83)90030-5
17. Choi JS, Brown TH. Central amygdala lesions block ultrasonic vocalization and freezing as conditional but not unconditional responses. Journal of Neuroscience. 2003;23: 8713–8721. doi:10.1523/jneurosci.23-25-08713.2003
18. Eilam D. Die hard: A blend of freezing and fleeing as a dynamic defense - Implications for the control of defensive behavior. Neuroscience and Biobehavioral Reviews. 2005. pp. 1181–1191. doi:10.1016/j.neubiorev.2005.03.027
19. Motta SC, Goto M, Gouveia F V., Baldo MVC, Canteras NS, Swanson LW. Dissecting the brain's fear system reveals the hypothalamus is critical for responding in subordinate conspecific intruders. Proc Natl Acad Sci U S A. 2009;106: 4870–4875. doi:10.1073/pnas.0900939106
20. Pagani JH, Rosen JB. The medial hypothalamic defensive circuit and 2,5-dihydro-2,4,5-trimethylthiazoline (TMT) induced fear: Comparison of electrolytic and neurotoxic lesions. Brain Res. 2009;1286: 133–146. doi:10.1016/j.brainres.2009.06.062

21. Smith JM, Price GR. The logic of animal conflict. Nature. 1973;246: 15–18. doi:10.1038/246015a0
22. Lima SL. Putting predators back into behavioral predator -prey interactions. Trends Ecol Evol. 2002;17: 70–75. doi:10.1016/S0169-5347(01)02393-X
23. Nowak MA, Sigmund K. Evolutionary Dynamics of Biological Games. Science. 2004. pp. 793–799. doi:10.1126/science.1093411
24. McNamara JM, Houston AI. Integrating function and mechanism. Trends Ecol Evol. 2009;24: 670–675. doi:10.1016/j.tree.2009.05.011
25. Hammond JI, Luttbeg B, Brodin T, Sih A. Spatial scale influences the outcome of the predator-prey space race between tadpoles and predatory dragonflies. Funct Ecol. 2012;26: 522–531. doi:10.1111/j.1365-2435.2011.01949.x
26. Ramesh D, Mitchell WA. Evolution of signalling through pursuit deterrence in a two-prey model using game theory. Anim Behav. 2018;146: 155–163. doi:10.1016/j.anbehav.2018.10.012
27. McNamara JM. Game Theory in Biology: Moving beyond Functional Accounts. American Naturalist. 2022;199: 179–193. doi:10.1086/717429
28. Alpern S, Gal S, Lee V, Casas J. A stochastic game model of searching predators and hiding prey. J R Soc Interface. 2019;16: 20190087. doi:10.1098/rsif.2019.0087
29. Chen S, Bao S, Yan L, Huang C. A Predation Behavior Model Based on Game Theory. arXiv preprint q-bio/0505026. 2005 [cited 14 Jul 2023]. Available: https://arxiv.org/abs/q-bio/0505026
30. Zoroa N, Fernández-Sáez MJ, Zoroa P. A foraging problem: Sit-and-wait versus active predation. Eur J Oper Res. 2011;208: 131–141. doi:10.1016/j.ejor.2010.08.001
31. Nash JF. Equilibrium points in n -person games. Proceedings of the National Academy of Sciences. 1950;36: 48–49. doi:10.1073/pnas.36.1.48
32. Lee D. Game theory and neural basis of social decision making. Nat Neurosci. 2008;11. doi:10.1038/nn2065
33. Mobbs D, Trimmer PC, Blumstein DT, Dayan P. Foraging for foundations in decision neuroscience: Insights from ethology. Nat Rev Neurosci. 2018;19: 419–427. doi:10.1038/s41583-018-0010-7
34. Ydenberg RC, Dill LM. The Economics of Fleeing from Predators. Adv Study Behav. 1986;16: 229–249. doi:10.1016/S0065-3454(08)60192-8
35. Ichijo H, Nakamura T, Yamamichi M. Nash equilibrium of attack and defense behaviors between predators and prey in game theory. The 46th Annual Meeting of the Japan Neuroscience Society. Sendai; 2023.
36. Doya K, Samejima K, Katagiri KI, Kawato M. Multiple model-based reinforcement learning. Neural Comput. 2002;14: 1347–1369. doi:10.1162/089976602753712972
37. De Roos AM, McCauley E, Wilson WG. Mobility versus density-limited predator-prey dynamics on different spatial scales. Proc R Soc Lond B Biol Sci. 1991;246: 117–122. doi:10.1098/rspb.1991.0132
38. Wilensky U, Evanston I. NetLogo: Center for connected learning and computer-based modeling. Northwestern University, Evanston, Illinois, US. 1999.
39. Wellman MP. Methods for Empirical Game-Theoretic Analysis (Extended Abstract). AAAI. 2006;980: 1552–1556. Available: www.aaai.org
40. Chen S, Bao S. A Game Theory Based Predation Behavior Model. International Conference on Game Theory. 2010 [cited 14 Jul 2023]. Available: http://dev.gtcenter.org/Archive/2010/Conf/Chen956.pdf
41. Ichijo H, Nakamura T, Kawaguchi M, Takeuchi Y. An evolutionary hypothesis of binary opposition in functional incompatibility about habenular asymmetry in vertebrates. Front Neurosci. 2017;10: 595. doi:10.3389/fnins.2016.00595
42. Mizumori SJY, Baker PM. The Lateral Habenula and Adaptive Behaviors. Trends in Neurosciences. 2017. pp. 481–493.
43. Yuste R, Bonhoeffer T. Morphological changes in dendritic spines associated with long-term synaptic plasticity. Annu Rev Neurosci. 2001;24: 1071–1089.

doi:10.1146/annurev.neuro.24.1.1071

44. Kanemoto M, Nakamura T, Sasahara M, Ichijo H. Stress-Related Neuronal Clusters in Sublenticular Extended Amygdala of Basal Forebrain Show Individual Differences of Positions. Front Neural Circuits. 2020;14: 29. doi:10.3389/fncir.2020.00029
45. Roberts RJV, Pop S, Prieto-Godino LL. Evolution of central neural circuits: state of the art and perspectives. Nat Rev Neurosci. 2022;23. doi:10.1038/s41583-022-00644-y
46. Tinbergen N. On aims and methods of ethology. Z Tierpsychol. 1963;20: 410–433. Available: www.brill.nl
47. Dawkins R, Krebs JR. Arms races between and within species. Proceedings of the Royal Society of London - Biological Sciences. 1979;205. doi:10.1098/rspb.1979.0081
48. Zoroa N, Fernández-Sáez MJ, Zoroa P. A foraging problem: Sit-and-wait versus active predation. Eur J Oper Res. 2011;208. doi:10.1016/j.ejor.2010.08.001
49. Ross CT, Winterhalder B. Sit-and-wait versus active-search hunting: A behavioral ecological model of optimal search mode. J Theor Biol. 2015;387: 76–87. doi:10.1016/j.jtbi.2015.09.022
50. Edelaar P, Jovani R, Gomez-Mestre I. Should I Change or Should I Go? Phenotypic Plasticity and Matching Habitat Choice in the Adaptation to Environmental Heterogeneity. Am Nat. 2017;190: 506–520. doi:10.1086/693345
51. Team RC. R: A Language and Environment for Statistical Computing. R Foundation for Statistical Computing. 2021.
52. Salecker J, Sciaini M, Meyer KM, Wiegand K. The nlrx r package: A next-generation framework for reproducible NetLogo model analyses. Methods Ecol Evol. 2019;10: 1854–1863. doi:10.1111/2041-210X.13286
53. Avis D, Rosenberg GD, Savani R, von Stengel B. Enumeration of Nash equilibria for two-player games. Econ Theory. 2009;42: 9–37. doi:10.1007/s00199-009-0449-x
54. Katz MW, Abramsky Z, Kotler BP, Roth I, Livne S, Altstein O, et al. A predator-prey foraging game: How does prey density influence tactics? Evol Ecol Res. 2014;16: 323–335.
55. Schmitz O. Predator and prey functional traits: Understanding the adaptive machinery driving predator-prey interactions. F1000Research. Faculty of 1000 Ltd; 2017. doi:10.12688/f1000research.11813.1
56. White JW, Rassweiler A, Samhouri JF, Stier AC, White C. Ecologists should not use statistical significance tests to interpret simulation model results. Oikos. 2014;123. doi:10.1111/j.1600-0706.2013.01073.x

Table 1. Variables of simulations

Variables	The sensory-motor algorithm
d	Sensory trait
d_w	d of predators (wolves)
d_s	d of prey (sheep)
D	Distance to detect the opponent*
D_w	D of predators (wolves)
D_s	D of prey (sheep)
	**The single-element motor algorithm**
K	Probability of a speed change
K_w	K of predators (wolves)
K_s	K of prey (sheep)
	**Common in both algorithms**
S	Motor trait for amount of speed change †
S_w	S of predators (wolves)
S_s	S of prey (sheep)
c_b	Behavioral cost coefficient in the NetLogo world
r	Reproductive coefficient of predators in the NetLogo world

* The distance to detect an opponent (D_i) ($i = w, s$) is d_i multiplied by the sensitivity factor f ($D_i = d_i \times f$, where $f = 10$).

† Motor traits are represented by the amount of speed change (S_i). When agents detect an opponent within radius D_i, they change their speed to $1 + S_i$ in the sensory-motor algorithm. The agents change their speed to $1 + S_i$ with a probability of K_i in the single-element motor algorithm.

Table 2. Measurements of simulations

**Measurements**	
p	Encounter probability in the grid world
E	Encounter frequency in the NetLogo world
N_w	Number of predators (wolves) in the NetLogo world
N_s	Number of prey (sheep) in the NetLogo world
p_w	Encounter probability of predators ($p_w = E/N_w$)
p_s	Encounter probability of prey ($p_s = E/(E + N_s)$)
P_w	Payoff of the predator in the NetLogo world ($P_w = p_w$)
P_s	Payoff of the prey in the NetLogo world ($P_s = 1 - p_s$)
P_{diff}	Difference between the prey and predator payoffs ($P_{diff} = P_s - P_w$)
$\bar{S}$	Nash equilibrium amount of speed change
$\bar{S}_w$	$\bar{S}$ of predators (wolves)
$\bar{S}_s$	$\bar{S}$ of prey (sheep)
$\bar{P}$	Nash equilibrium payoff
$\bar{P}_w$	$\bar{P}$ of predators (wolves)
$\bar{P}_s$	$\bar{P}$ of prey (sheep)
$\bar{o}$	Occurrence probability of a Nash equilibrium behavior
$\bar{o}_w$	$\bar{o}$ of predators (wolves)
$\bar{o}_s$	$\bar{o}$ of prey (sheep)

Table 3. Occurrence probabilities of behavioral switching in multiple predators and prey game when both the D_w and D_s are 2.

**Agent**	$\bar{S}_{w,1} = 1$ (chase)	$\bar{S}_{w,2} = -1$ (ambush)
Predator	0.462	0.538

**Agent**	$\bar{S}_{s,1} = 1$ (escape)	$\bar{S}_{s,2} = -1$ (freeze)
Prey	0.605	0.395

The Nash equilibrium is a mixed strategy when both D_w and D_s are 2; agents switch their behaviors randomly with the indicated occurrence probabilities.

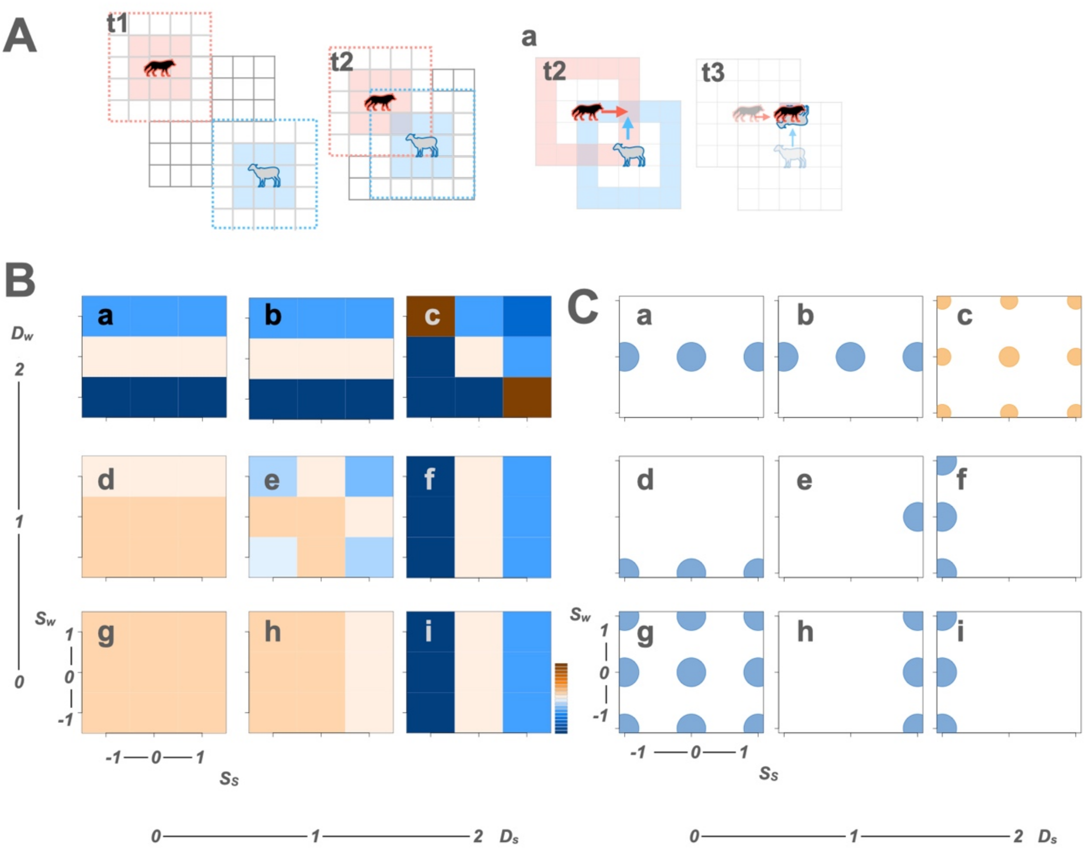

Fig. 1

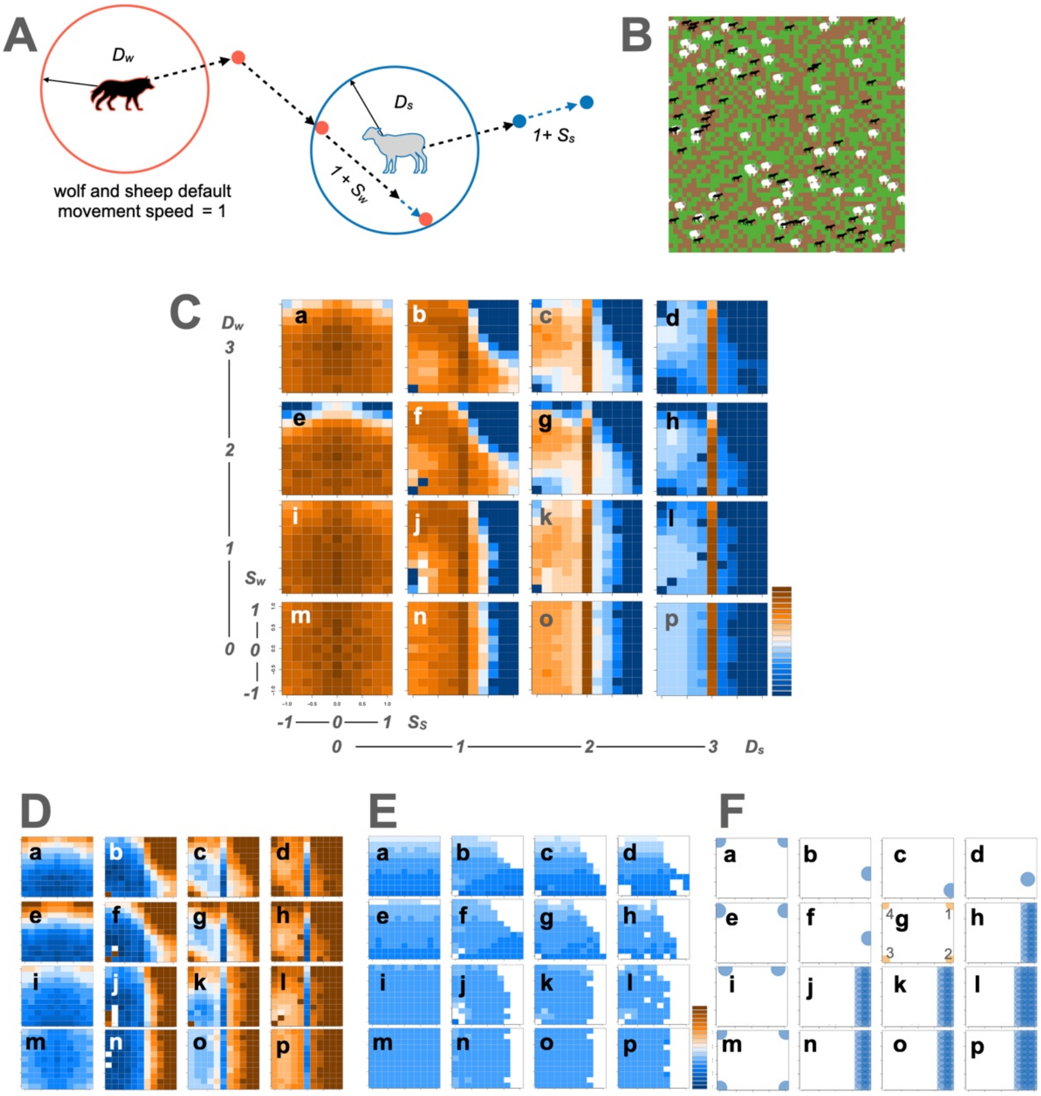
A
B
C
D
E
F
D_w
D_s
$1+ S_s$
$1 + S_w$
wolf and sheep default movement speed = 1
S_w
S_s

Fig. 2

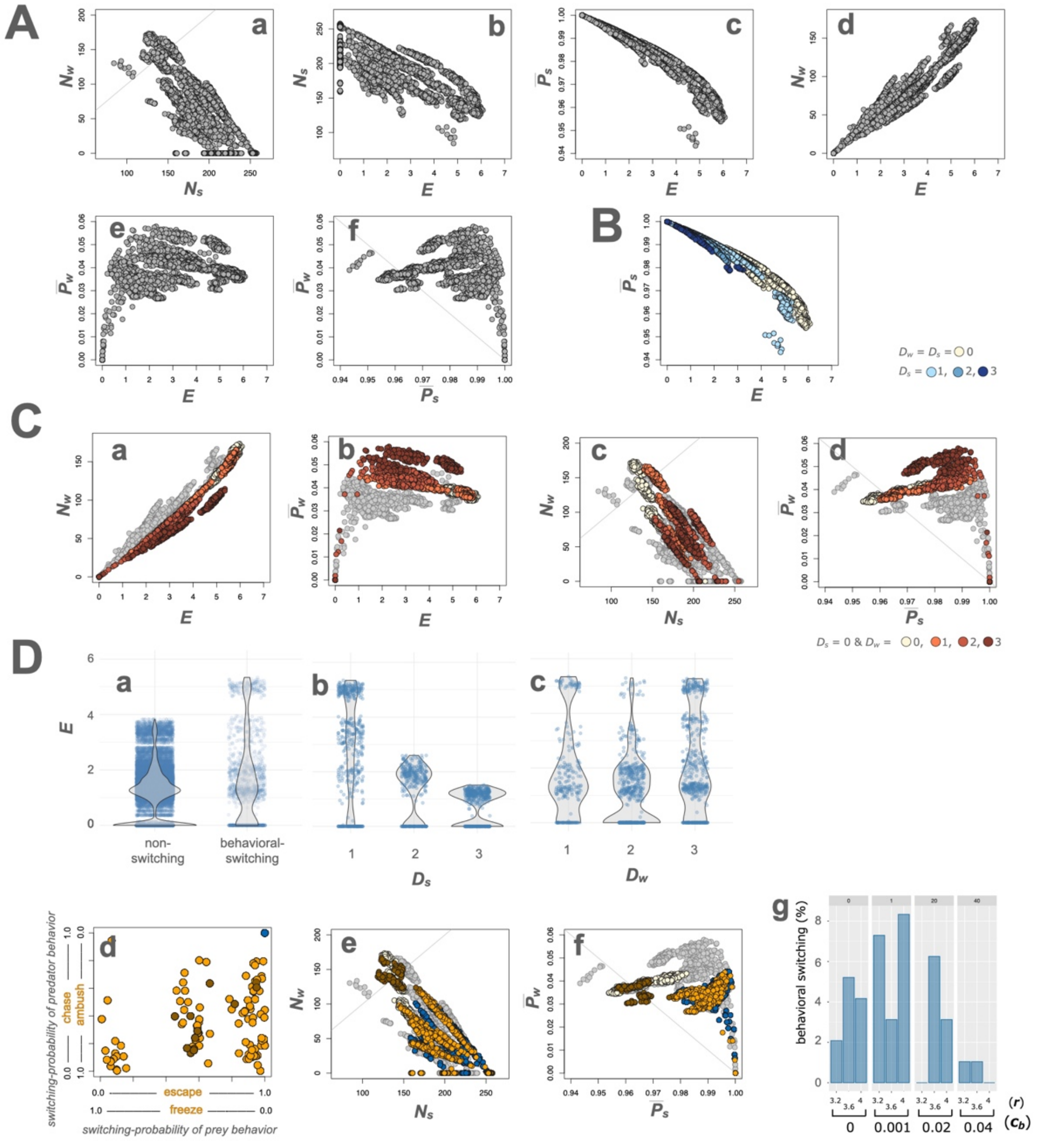
A
B
C
D
N_w
N_s
E
$\overline{P}_s$
$\overline{P}_w$
$D_w = D_s = 0$
$D_s = 1, 2, 3$
$D_s = 0$ & $D_w = 0, 1, 2, 3$
non-switching
behavioral-switching
D_s
D_w
switching-probability of predator behavior
chase
ambush
escape
freeze
switching-probability of prey behavior
behavioral switching (%)
(r)
(c_b)
0
0.001
0.02
0.04

Fig. 3

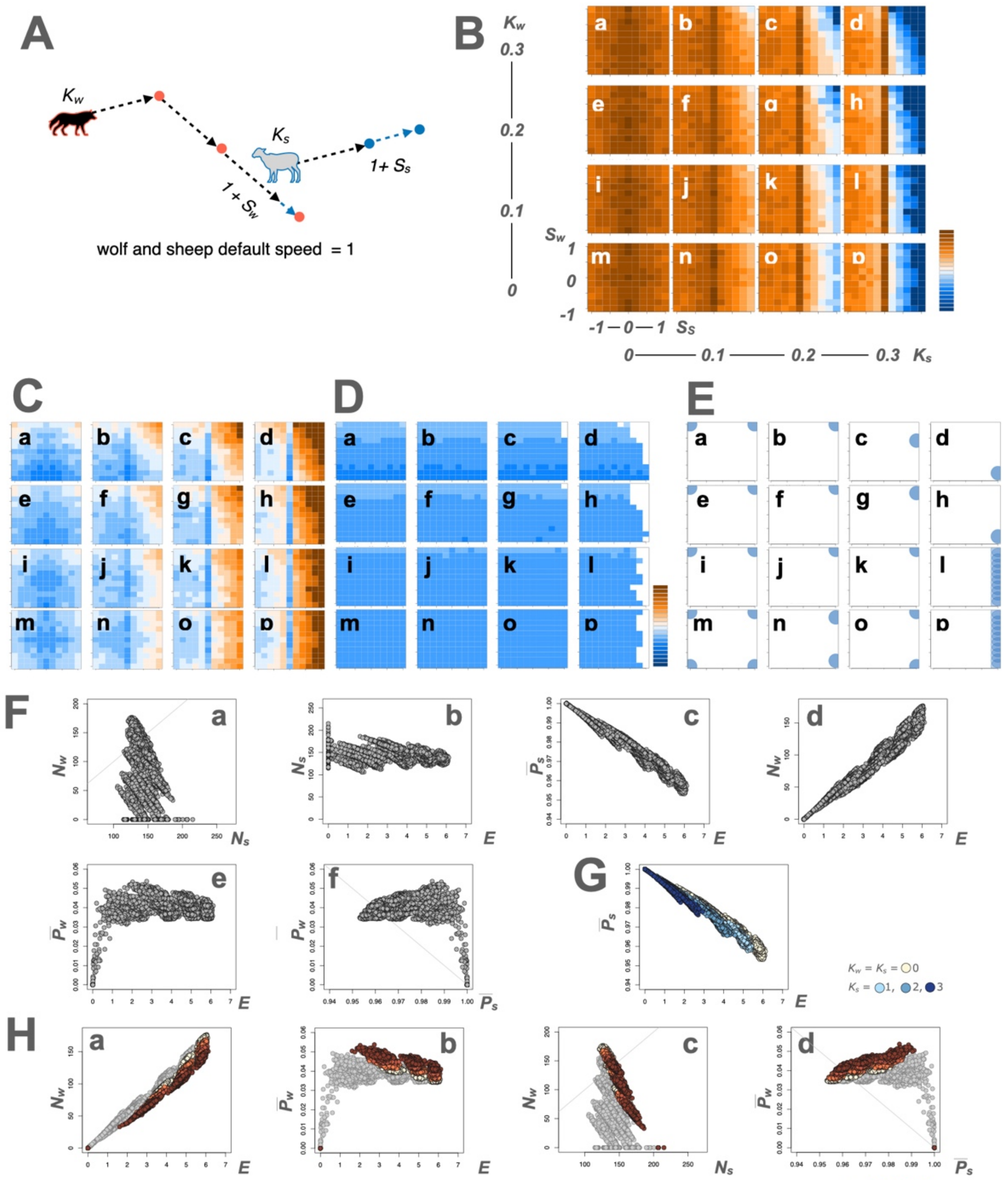
A
B
C
D
E
F
G
H
wolf and sheep default speed = 1
K_w
K_s
$1+S_w$
$1+S_s$
S_w
S_s
0.3
0.2
0.1
0
0 — 0.1 — 0.2 — 0.3 K_s
-1 — 0 — 1 S_s
N_w
N_s
$\overline{P}_s$
$\overline{P}_w$
E
$K_w = K_s =$ 0
$K_s =$ 1, 2, 3
$K_s = 0$ & $K_w =$ 0, 1, 2, 3

Fig. 4

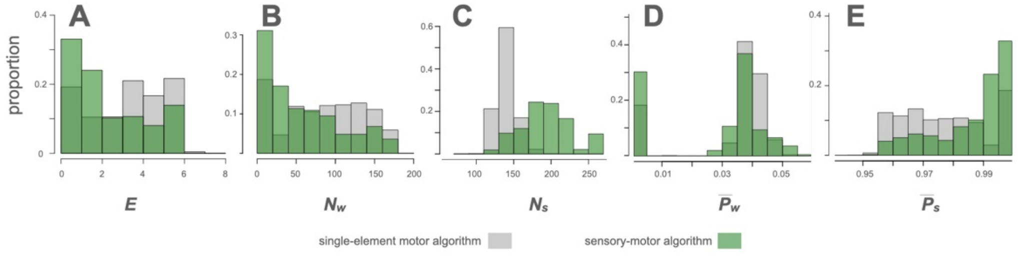

A
B
C
D
E
proportion
E
N_w
N_s
$\overline{P}_w$
$\overline{P}_s$
single-element motor algorithm
sensory-motor algorithm

Fig. 5

Figure legends

Fig. 1. Attack and defense behaviors with the sensory-motor algorithm between one predator and one prey.

A illustrates attack and defense behaviors between one predator and prey (agents) in the grid world. Three time steps are shown (**t1**, **t2**, **t3**). At **t1**, agents detect an opponent within a specific range. The dotted lines illustrate their sensory abilities of detection ranges ($D_w = 2$ for the predator (wolf) and $D_s = 2$ for the prey (sheep)). When no opponent is detected, both agents move 1 grid per time step. The shaded grids represent possible positions they could move to at the next time step. However, if detection occurs at **t2**, they adjust their speeds by S_i ($i = w$ for wolf and s for sheep), changing their speed to $1 + S_i$. In case $S_w = S_s = 1$ (**Aa**), the wolf chases the sheep ($1 + S_w = 2$), and the sheep escapes ($1 + S_s = 2$). If both agents occupy the same grid (as shown by arrows in **Aa**-**t2**), the wolf eats the sheep at **t3**.

B illustrates landscape diagrams that depict encounter probabilities based on combinations of sensory abilities (D_i). The diagrams show nine combinations, ranging from $D_s = 0$ and $D_w = 2$ (**a**) to $D_s = 2$ and $D_w = 0$ (**i**). The x-axis represents the sheep's speed change (S_s), while the y-axis represents the wolf's speed change (S_w). Color-coded squares within each landscape represent the encounter probabilities (p) associated with different combinations of D_i and S_i. High and low probabilities are indicated in dark brown and dark blue, respectively.

C presents the Nash equilibrium behaviors. The positions of circles on the x and y axes indicate the prey's and predator's Nash equilibrium motor abilities ($\overline{S}_s$ and $\overline{S}_w$), respectively, corresponding to the same coordinates as in the encounter probability landscapes (**a**–**i**). The diameter of each circle reflects the occurrence probability of these behaviors ($\overline{o_i}$). Blue circles represent cases where Nash equilibrium behavior is uniquely determined (diameter of 1), while orange circles indicate cases where behaviors are performed probabilistically.

Fig. 2. Attack and defense behaviors with the sensory-motor algorithm between multiple predators and prey.

A illustrates the agents' behaviors ((predators (wolves, w) and prey (sheep, s)) in the

NetLogo world. Both agents move at a default speed of 1 per time step. When the opponents are detected within a specific range (D_i) ($i = w$ or s), speeds change by S_i, resulting in new speeds of $1 + S_i$. D_w and D_s are shown as red and blue circles, respectively. In this illustration ($S_w > 0$ and $S_s > 0$), the predator and prey increase their speeds, corresponding to chase and escape, respectively.

B shows the NetLogo world, where grass grows on brown soil. Wolves (in black) move, lose energy, gain energy by eating prey, reproduce, and die if their energy reaches zero. Sheep (in white) move, eat grass to gain energy, reproduce, and die either from predation or when their energy reaches zero.

C represents landscape diagrams showing encounter frequency (E) between predators and prey with the values of the behavioral cost coefficients (c_b =0.02) and reproductive coefficients of the predators ($r = 3.6$). Sixteen landscapes depict different combinations of agents' detection ranges, D_i (= 0, 1, 2, 3), with the x-axis for the prey's speed change (S_s) and the y-axis for the predator's speed change (S_w). Darker brown represents higher frequencies and dark blue indicates lower frequencies.

D and **E** show landscape diagrams for prey (P_s) and predator (P_w) payoffs, following the same D_i and S_i combinations, and color codes as in **C**.

F depicts Nash equilibrium behaviors. Circle positions on the x and y axes represent the Nash equilibrium speed change of prey ($\overline{S}_s$) and predators ($\overline{S}_w$), respectively. Circle diameters reflect the occurrence probabilities ($\overline{o_i}$) of these behaviors. Blue indicates uniquely determined behaviors (diameter 1). Orange represents probabilistic behaviors ($0 < \overline{o_i} < 1$) which result in four behavioral pairs located at the corners of the landscape: chase and escape (1), ambush and escape (2), ambush and freeze (3), and chase and freeze (4) in **Fg**.

Fig. 3. Effects of Nash equilibrium attack/defense behaviors with the sensory-motor algorithm on encounters, agent numbers, and payoffs.

A Scatter plots illustrate the relationships between the number of agents (N_i), encounter frequency (E), and payoff ($\overline{P}_i$) at Nash equilibrium. For all c_b and r, data points from the Nash equilibrium are plotted in gray. **Aa** Scatter plot with N_s on the x-axis and N_w on the y-axis, along the line $N_s = N_w$. **Ab**, **Ac**, **Ad**, and **Ae** plot E on the x-axis against N_s, $\overline{P}_s$, N_w, and $\overline{P}_w$ on the y-axis, respectively. **Af** Scatter plot with $\overline{P}_s$ on the x-axis and $\overline{P}_w$

on the y-axis, along the line $\bar{P}_s + \bar{P}_w = 1$.

B and **C** show the effects of D_i on E, N_i, and $\bar{P}_i$.

B Scatter plot of E versus $\bar{P}_s$, with light blue, blue, and dark blue points indicating D_s = 1, 2, and 3, respectively. Beige points represent $D_s = D_w = 0$, where neither predators nor prey attack or defend. **Ca** Scatter plot of E versus N_w. **Cb** Scatter plot of E versus $\bar{P}_w$. **Cc** Scatter plot of N_s versus N_w. **Cd** Scatter plot of $\bar{P}_s$ versus $\bar{P}_w$. In **Ca**–**d**, orange, brown, and dark brown points represent D_w = 1, 2, and 3, respectively, with $D_s = 0$.

D shows violin plots of the effects of behavioral switching on E.

Da Large E values (> 4) result from behavioral switching. **Db** Large E values depend on D_s, with $E > 4$ when $D_s = 1$. **Dc** Large E values are independent of D_w. **Dd** Scatter plot of $\bar{o}_s$ versus $\bar{o}_w$. **De** Scatter plot of N_s versus N_w. **Df** Scatter plot of $\bar{P}_s$ versus $\bar{P}_w$. Dark orange, orange, and blue points indicate behavioral switching with $E > 4$, behavioral switching with $E \leq 4$, and no behavioral switching, respectively. **Dg** Bar plots show the effect of c_b on switching behavior, with percentages of switching for different c_b and r values.

Fig. 4. Effects of Nash equilibrium speed changes with the single-element motor algorithm on encounters, agent numbers, and payoffs.

A Stochastic speed change is defined by the probability of speed change (K_i) and S_i. The agent's life history in this model includes movement, feeding, energy gain/loss, reproduction, and death, similar to the sensory-motor algorithm model.

B depicts landscape diagrams of E in the single-element motor algorithm. The landscapes represent combinations of K_i (= 0, 0.1, 0.2, and 0.3), and color-coded. For example, **a** shows $K(0, 0.3)$, indicating $K_s = 0$ and $K_w = 0.3$, while **b** to **p** represents different combinations of K_i values.

C and **D** show the landscape diagrams of P_s and P_w, respectively.

E displays $\bar{S}_i$, with circles on the x and y axes indicating $\bar{S}_s$ and $\bar{S}_w$ at the same coordinates as the payoff landscape.

F represents scatter plots of N_i, E, and $\bar{P}_i$ of the Nash equilibrium speed changes. **Fa** shows N_s on the x-axis and N_w on the y-axis along the line $N_s = N_w$. **Fb**, **Fc**, **Fd**, and **Fe** plot E on the x-axis against N_s, $\bar{P}_s$, N_w, and $\bar{P}_w$ on the y-axis, respectively. **Ff** plots $\bar{P}_s$ on

the x-axis and $\bar{P}_w$ on the y-axis along the line $\bar{P}_s + \bar{P}_w = 1$.

G and **H** represent the effects of K_i on E, N_i, and $\bar{P}_i$.

G shows a scatter plot with E on the x-axis and $\bar{P}_s$ on the y-axis, with light blue, blue, and dark blue points representing $K_s = 0.1$, 0.2, and 0.3, respectively. Beige points indicate $K_s = K_w = 0$. **Ha** represents a scatter plot with E on the x-axis and N_w on the y-axis, with orange, brown, and dark brown points representing $K_w = 0.1$, 0.2, and 0.3, respectively, when $K_s = 0$. **Hb**, **Hc**, and **Hd** represent scatter plots of E versus $\bar{P}_w$, N_s versus N_w, and $\bar{P}_s$ versus $\bar{P}_w$.

Fig. 5. Comparison between the single-element motor and sensory-motor algorithms.

E, N_i, and $\bar{P}_i$ values of the single-element motor and sensory-motor algorithms are compared using histograms. **A**, **B**, **C**, **D**, and **E** display the proportion of instances in histograms of E, N_w, N_s, $\bar{P}_w$, and $\bar{P}_s$, respectively. The values for the single-element motor algorithm are depicted in gray, whereas those for the sensory-motor algorithm are shown in green. Compared to the single-element motor algorithm, the sensory-motor algorithm decreases E (**A**) and N_w (**B**) while increasing N_s (**C**). The sensory-motor algorithm leads to a larger proportion of $\bar{P}_w = 0$, indicating predator extinctions (**D**), and an increase of $\bar{P}_s$ (**E**).

Supplementary Tables and Figures

Supplementary table 1

Supplementary table 2

Supplementary table 3

Supplementary table 4

Supplementary Fig. 1

Supplementary Fig. 2

Supplementary Fig. 3

Supplementary Fig. 4

Supplementary Fig. 5

Supplementary Fig. 6

Supplementary Fig. 7

Supplementary Fig. 8

Supplementary Fig. 9

Supplementary Fig. 10

Supplementary Fig. 11

Supplementary Fig. 12

Supplementary Fig. 13

Supplementary Fig. 14

Supplementary Fig. 15

Supplementary Fig. legends

Supplementary Programs

EncounterProbabilities_gridworld.txt (R script)

EncounterProbabilities_sensory_motor.nlogo (NetLogo program)

NashEq_ActualMesurement_sensory_motor.nlogo (NetLogo program)

EncounterProbabilities_single_element_motor.nlogo (NetLogo program)

NashEq_ActualMesurement_single_element_motor.nlogo (NetLogo program)

Supplementary Source Data

PayoffMatrices_grid_world.xlsx

NashEq_Si_grid_world.xlsx

PayoffMatrices_r3.2-4.0_c0_run1-6.xlsx
PayoffMatrices_r3.2-4.0_c1_run1-6.xlsx
PayoffMatrices_r3.2-4.0_c20_run1-6.xlsx
PayoffMatrices_r3.2-4.0_c40_run1-6.xlsx
NashEq_Si_c0_r3.2-4.0.xlsx
NashEq_Si_c1_r3.2-4.0.xlsx
NashEq_Si_c20_r3.2-4.0.xlsx
NashEq_Si_c40_r3.2-4.0.xlsx
cb_r_Di_oi1_Si1_Si2_sensory_motor.xlsx
NashEq_ActualMesurement_sensory_motor.csv

PayoffMatrices_single_element_motor_r3.2-4.0_c0_run1-6.xlsx
PayoffMatrices_single_element_motor_r3.2-4.0_c1_run1-6.xlsx
PayoffMatrices_single_element_motor_r3.2-4.0_c20_run1-6.xlsx
PayoffMatrices_single_element_motor_r3.2-4.0_c40_run1-6.xlsx
single_element_motor_NashEq_Si_c0_wr3.2-4.0.xlsx
single_element_motor_NashEq_Si_c1_wr3.2-4.0.xlsx
single_element_motor_NashEq_Si_c20_wr3.2-4.0.xlsx
single_element_motor_NashEq_Si_c40_wr3.2-4.0.xlsx
cb_r_Ki_oi1_Si1_Si2_single_element_motor.xlsx
NashEq_ActualMesurement_single_element_motor.csv

source_data.pdf

Supplementary Table 1.

Constant values used in simulations involving multiple agents with the sensory-motor algorithm

**Constants**	
Initial number of predators (wolf)	60
Initial number of prey (sheep)	60
Energy gained by a predator eating a prey (wolf-gain-from-food)	8
Energy gained by a prey from eating grass (sheep gain from food)	1
Reproduction coefficient of prey (sheep-reproduce)	9
Time for grass to grow (grass-regrowth-time)	30
Movement speed	1
Coefficient of sensory trait (factor sensitivity, f)	10
Cost associated with basal metabolism (c_e)	0.1
Movement cost coefficient (c_m)	0.15

Supplementary Table 2.

Variable values used in simulations involving multiple agents with the sensory-motor algorithm

Variables	values
Sensory trait (d)	
d of wolves (d_w)	0, 0.1, 0.2, 0.3
d of sheep (d_s)	0, 0.1, 0.2, 0.3
Distance to detect the opponent (D) *	
D of wolves (D_w)	0, 1, 2, 3
D of sheep (D_s)	0, 1, 2, 3
Motor trait (S) for amount of speed change (S) †	
S of wolves: S_w	-1.0, -0.8, -0.6, -0.4, -0.2, 0.0, 0.2, 0.4, 0.6, 0.8, 1.0
S of sheep: S_s	-1.0, -0.8, -0.6, -0.4, -0.2, 0.0, 0.2, 0.4, 0.6, 0.8, 1.0
Behavioral cost coefficient (c_b) ‡	0, 0.001, 0.02, 0.04
Reproductive coefficient of predator (r)	3.2, 3.6, 4.0

*Distance to detect an opponent (D_i) ($i = w, s$) is d_i multiplied by factor-sensitivity, f ($D_i = d_i \times f$, where $f = 10$).

† When agents detect the opponent within D_i, their movement changes to $1 + S_i$.

‡ Cost dependent on the sensory and motor traits is the sum of the sensory and motor costs. The sensory cost is d_i multiplied by behavioral cost coefficient (c_b). Th motor cost is absolute value of S_i (abs(S_i)) multiplied by c_b. Thus, it is (d_i + (abs(S_i))*c_b.

Supplementary Table 3.

Constant values used in simulations involving multiple agents with the single-element motor algorithm

**Constants**	
Initial number of predators (wolf)	60
Initial number of prey (sheep)	60
Energy gained by a predator eating a prey (wolf-gain-from-food)	8
Energy gained by a prey from eating grass (sheep gain from food)	1
Reproduction coefficient of prey (sheep-reproduce)	9
Time for grass to grow (grass-regrowth-time)	30
Movement speed	1
Cost associated with basal metabolism (c_e)	0.1
Movement cost coefficient (c_m)	0.15

Supplementary Table 4.

Variable values used in simulations involving multiple agents with the single-element motor algorithm

Variables	values
Probability of speed change (K)	
K of wolves (K_w)	0, 0.1, 0.2, 0.3
K of sheep (K_s)	0, 0.1, 0.2, 0.3
Amount of speed change (S) †	
S of wolves: S_w	-1.0, -0.8, -0.6, -0.4, -0.2, 0.0, 0.2, 0.4, 0.6, 0.8, 1.0
S of sheep: S_s	-1.0, -0.8, -0.6, -0.4, -0.2, 0.0, 0.2, 0.4, 0.6, 0.8, 1.0
Behavioral cost coefficient (c_b) ‡	0, 0.001, 0.02, 0.04
Reproductive coefficient of predator (r)	3.2, 3.6, 4.0

† Agents change their speed to 1 + S_i with a probability of K_i.

‡ The costs dependent on the probability and amount of speed change are defined as the sum of K_i and absolute value of the motor trait (abs(S_i)) multiplied by c_b. Thus, it is (K_i + (abs(S_i))*c_b.

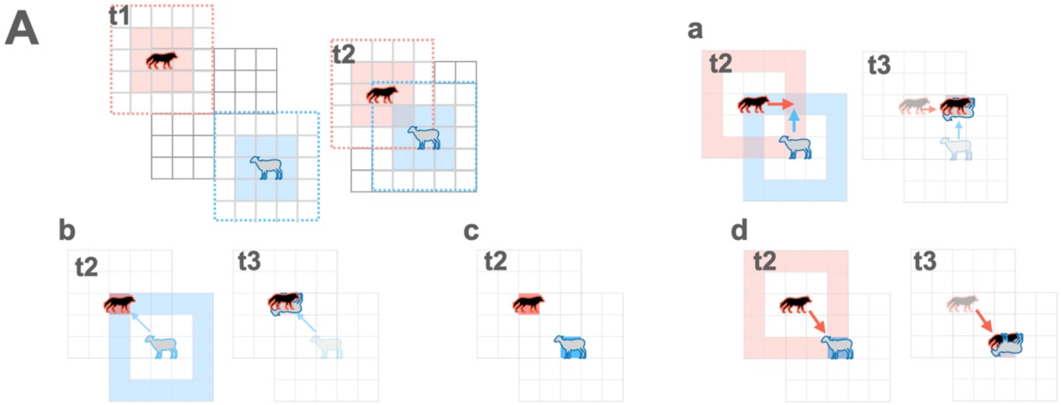

Supplementary Fig. 1

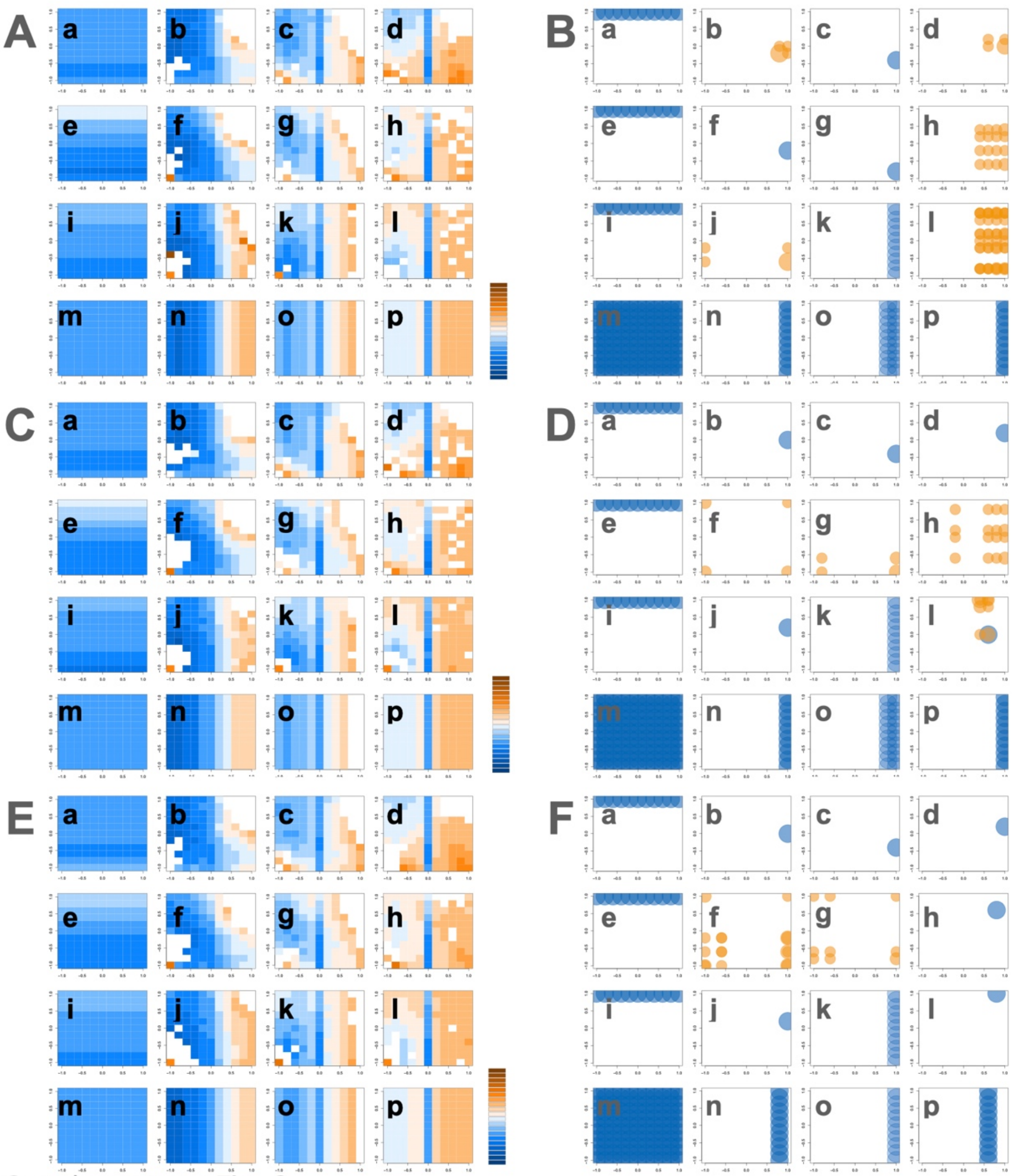

Supplementary Fig. 2

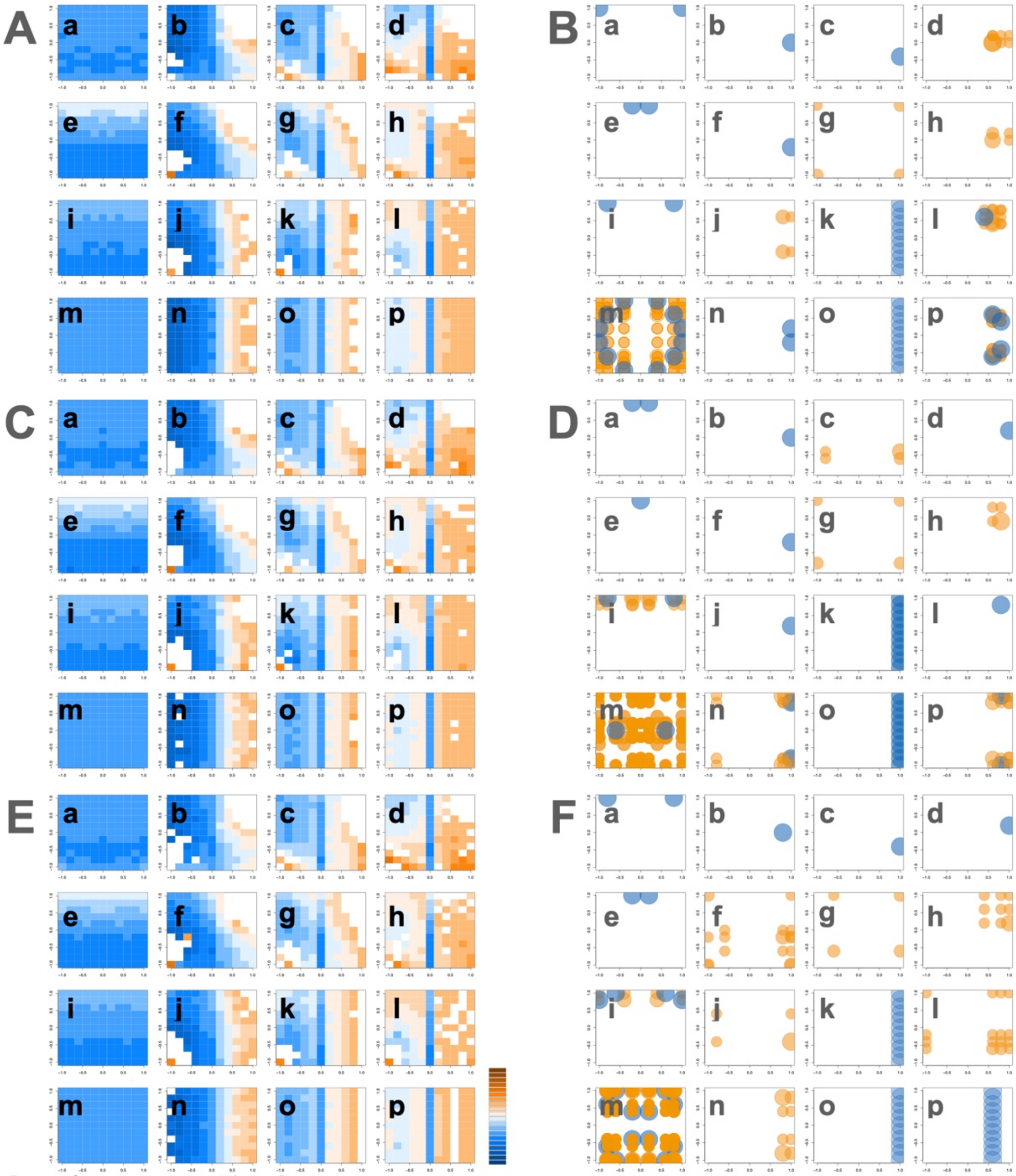

Supplementary Fig. 3

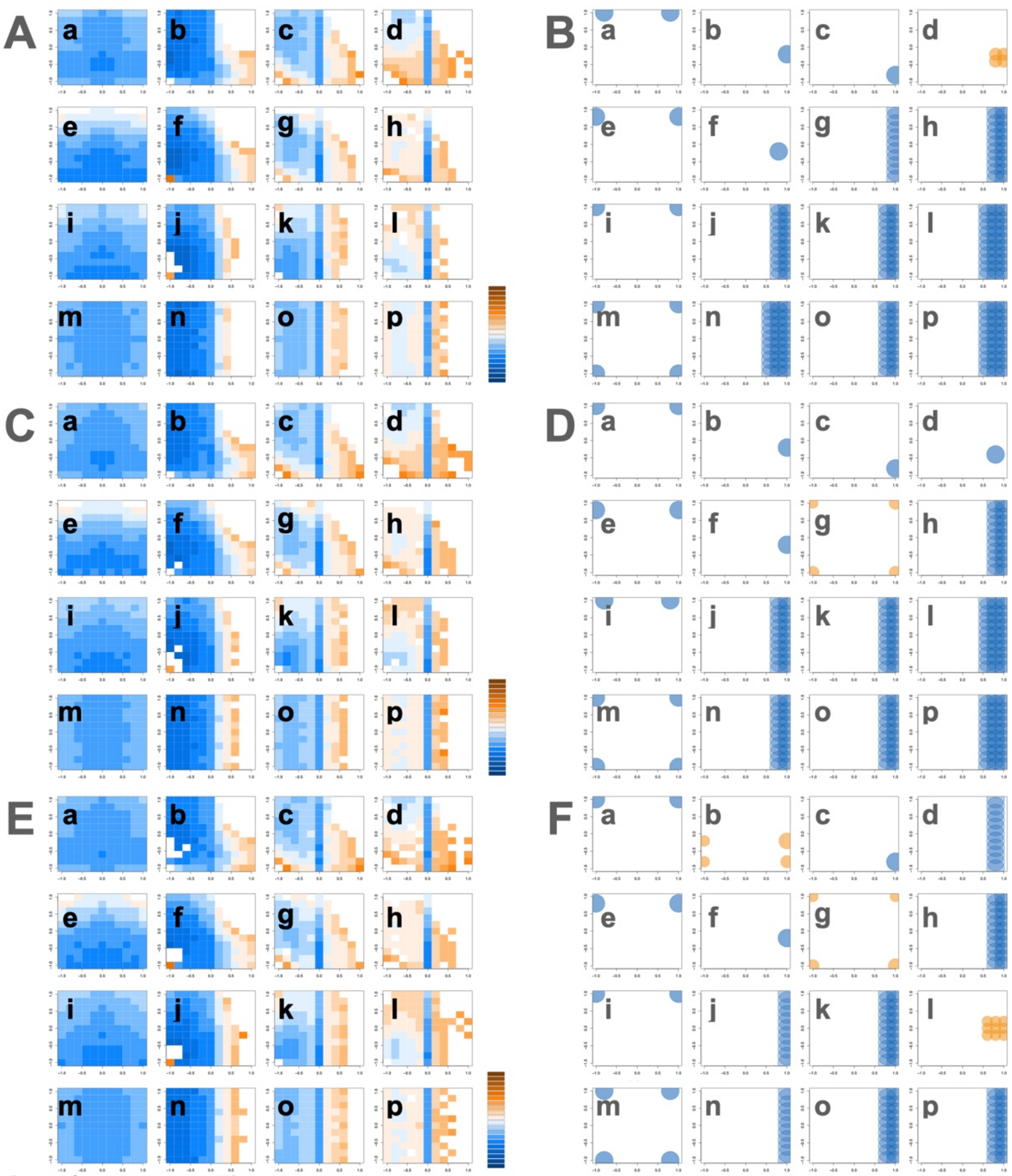

Supplementary Fig. 4

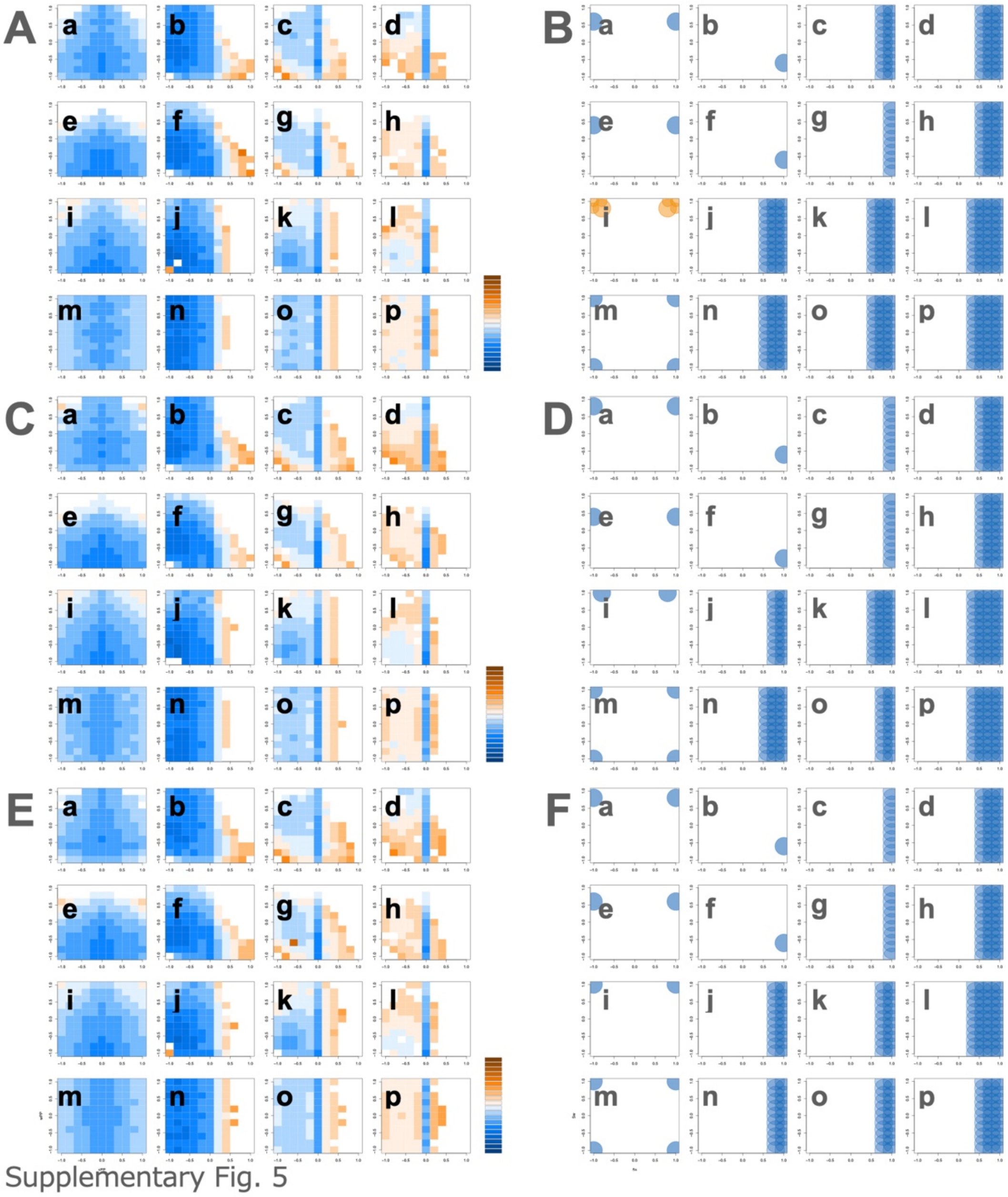

Supplementary Fig. 5

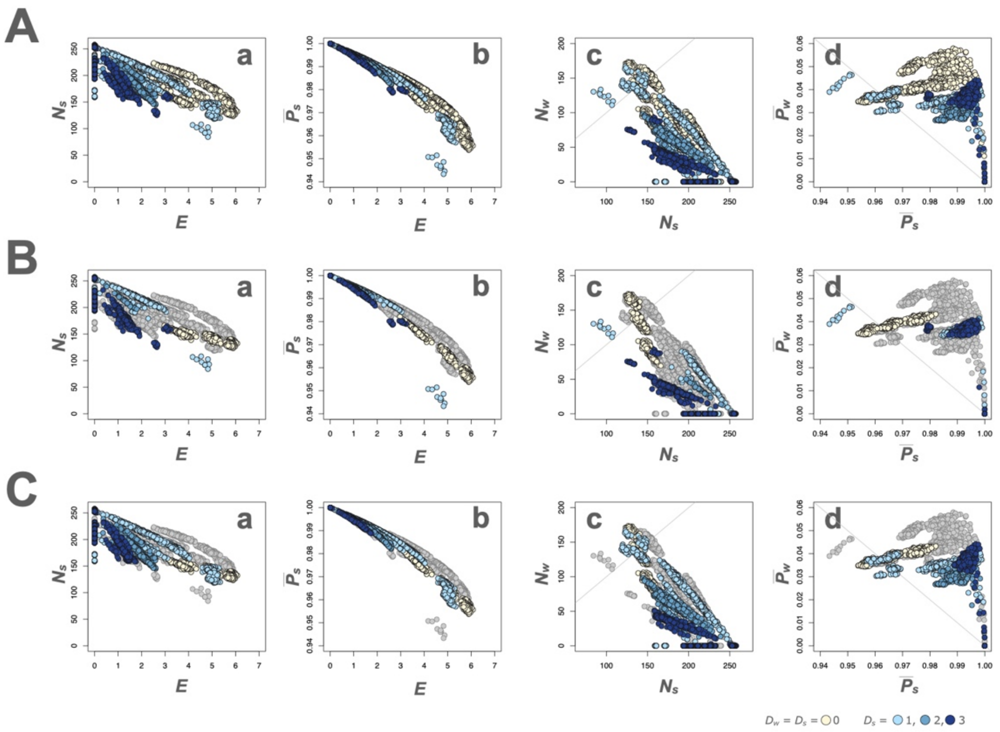

Supplementary Fig. 6

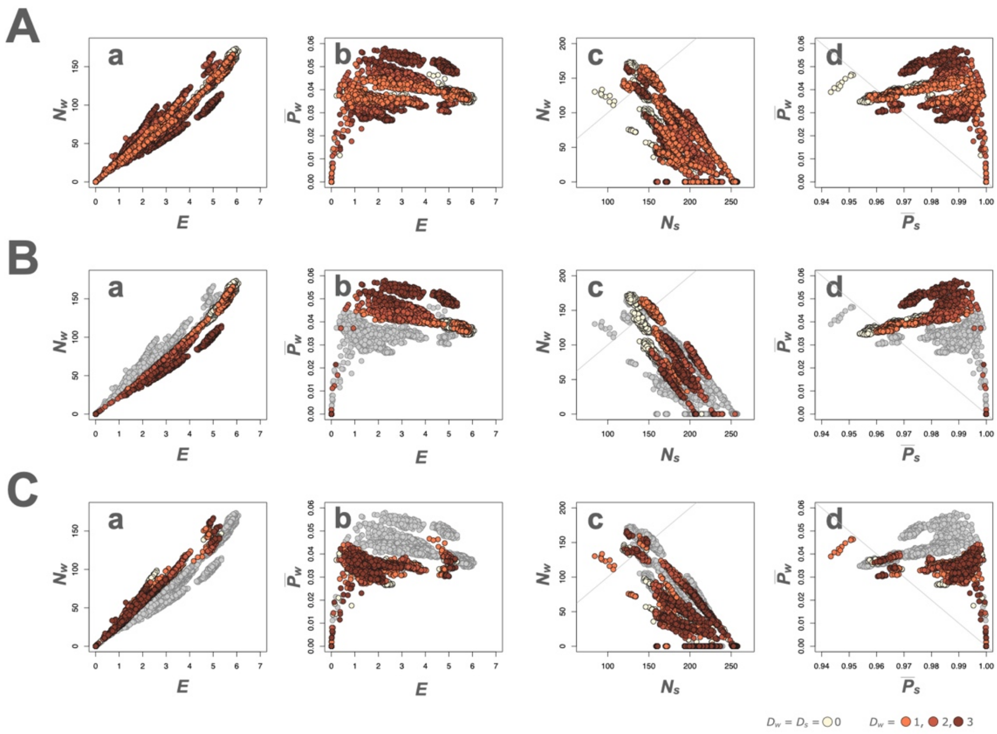

Supplementary Fig. 7

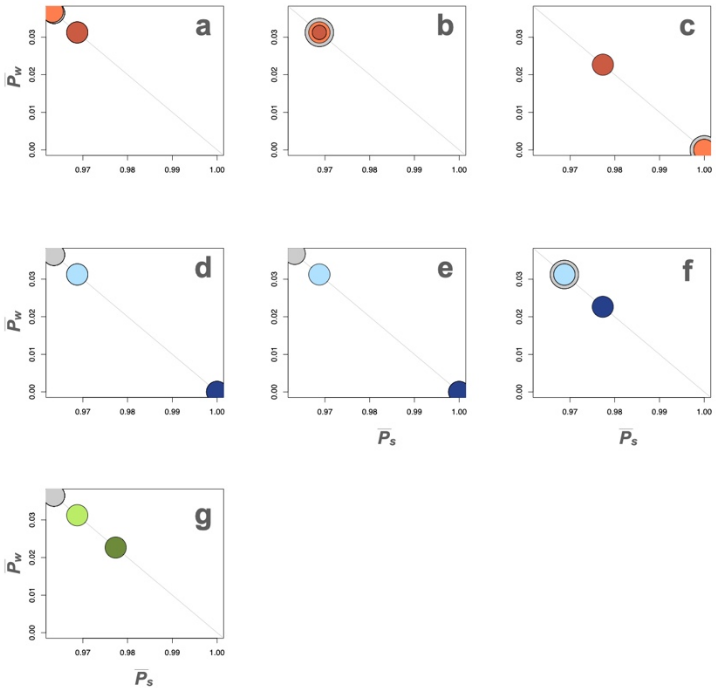

Supplementary Fig. 8

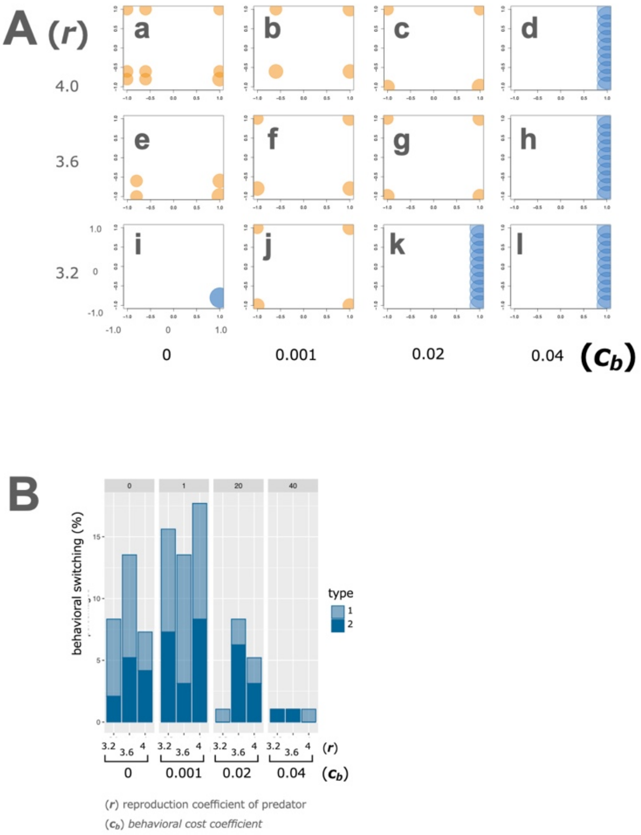

Supplementary Fig.9

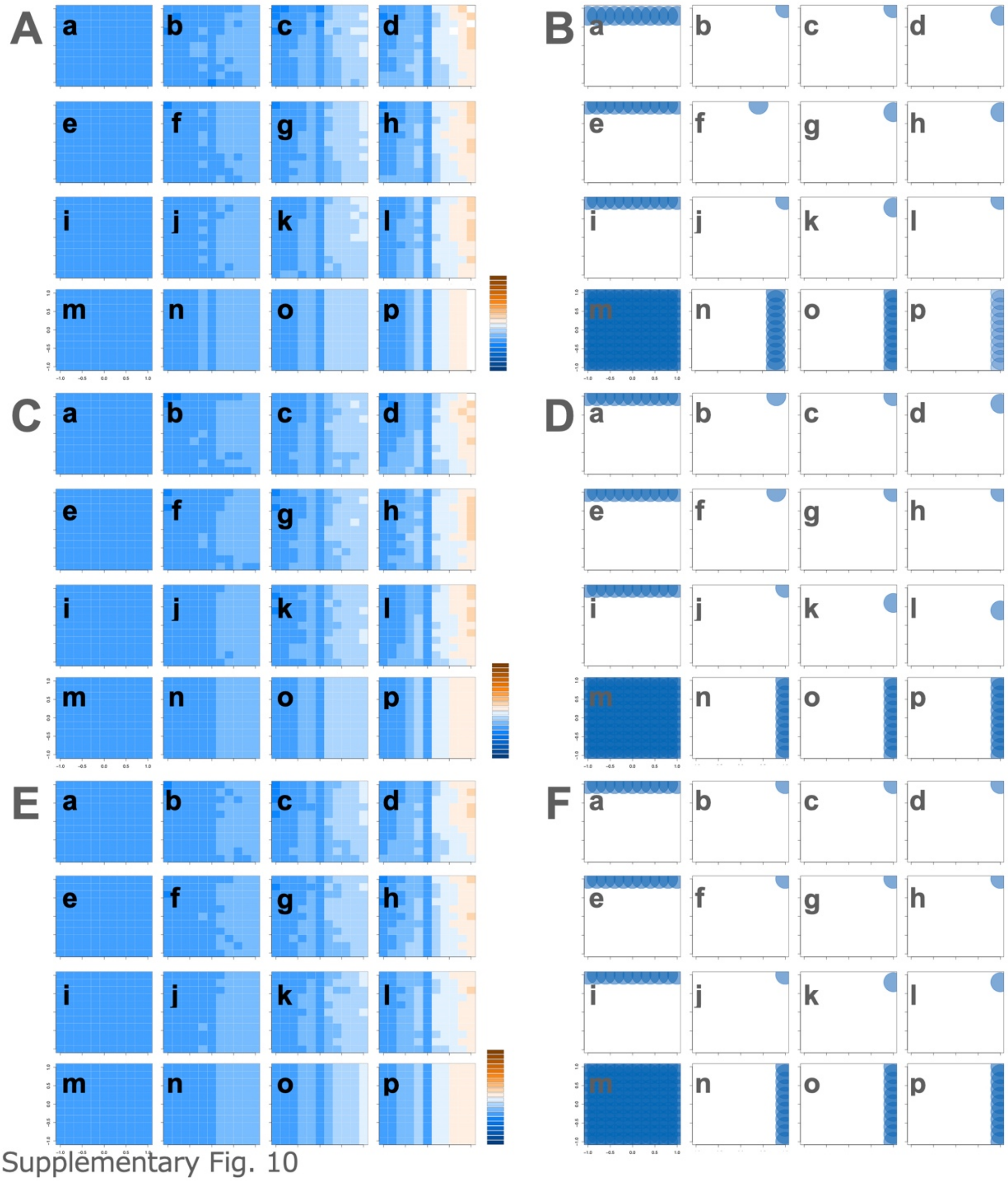

Supplementary Fig. 10

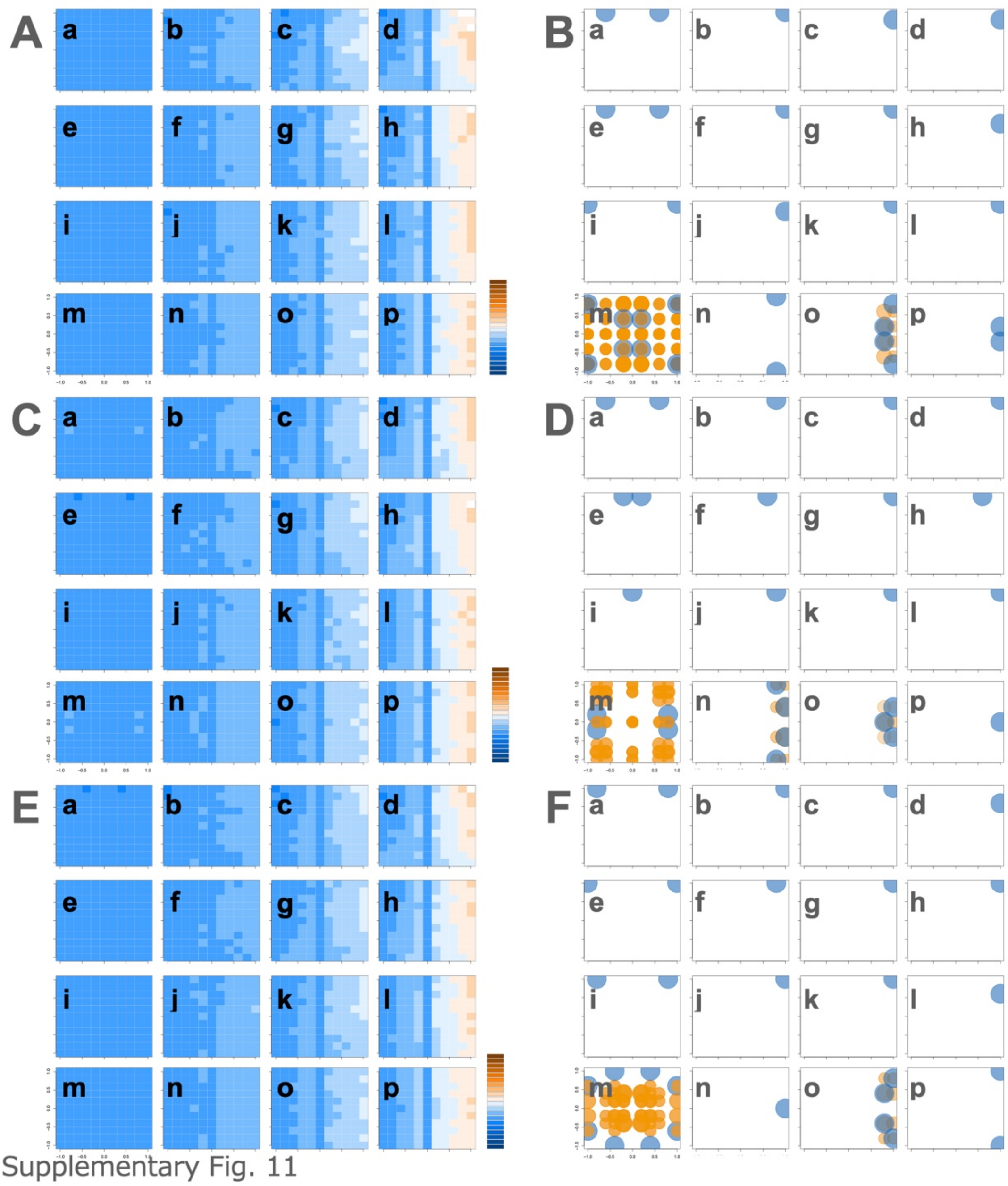

Supplementary Fig. 11

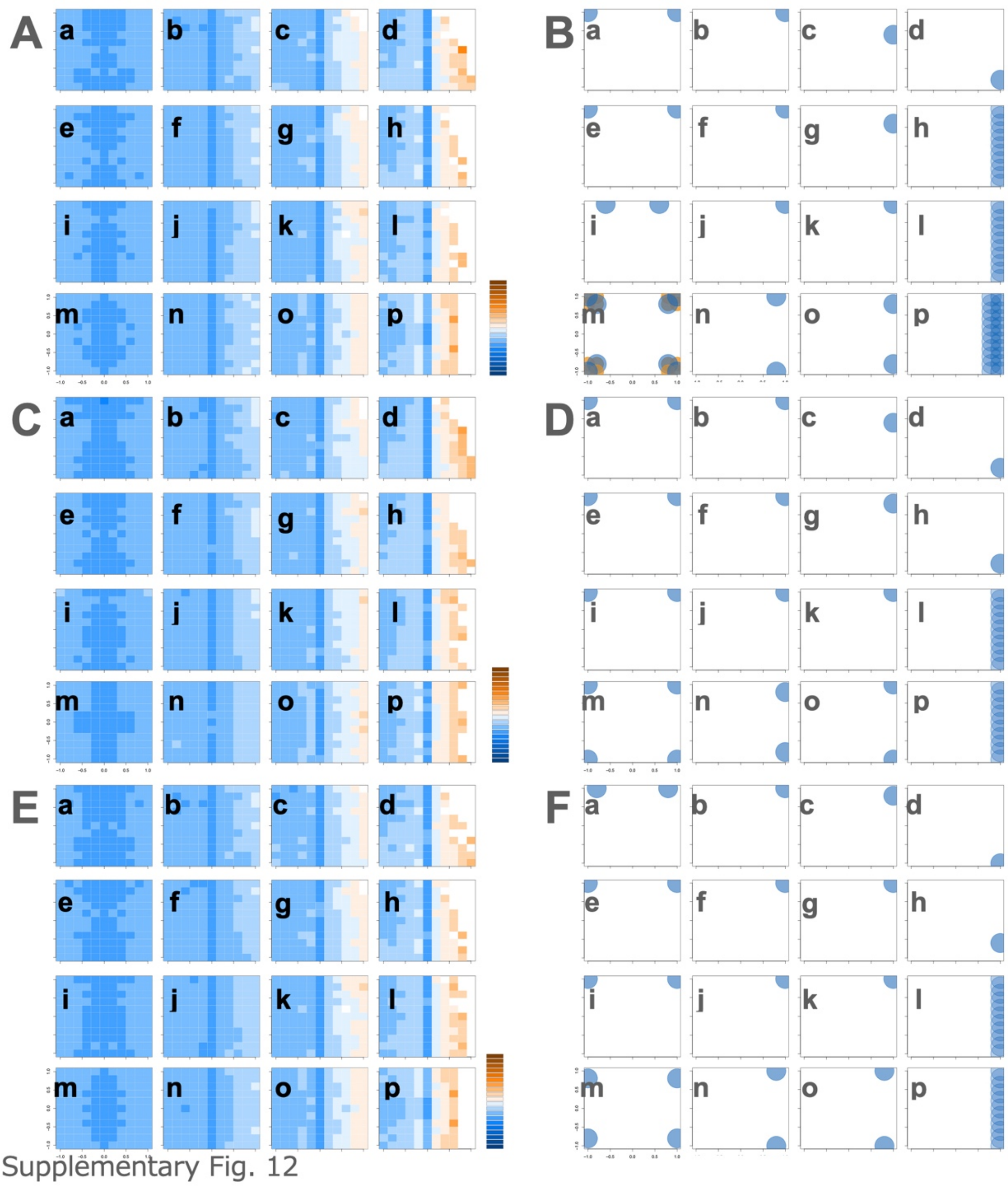

Supplementary Fig. 12

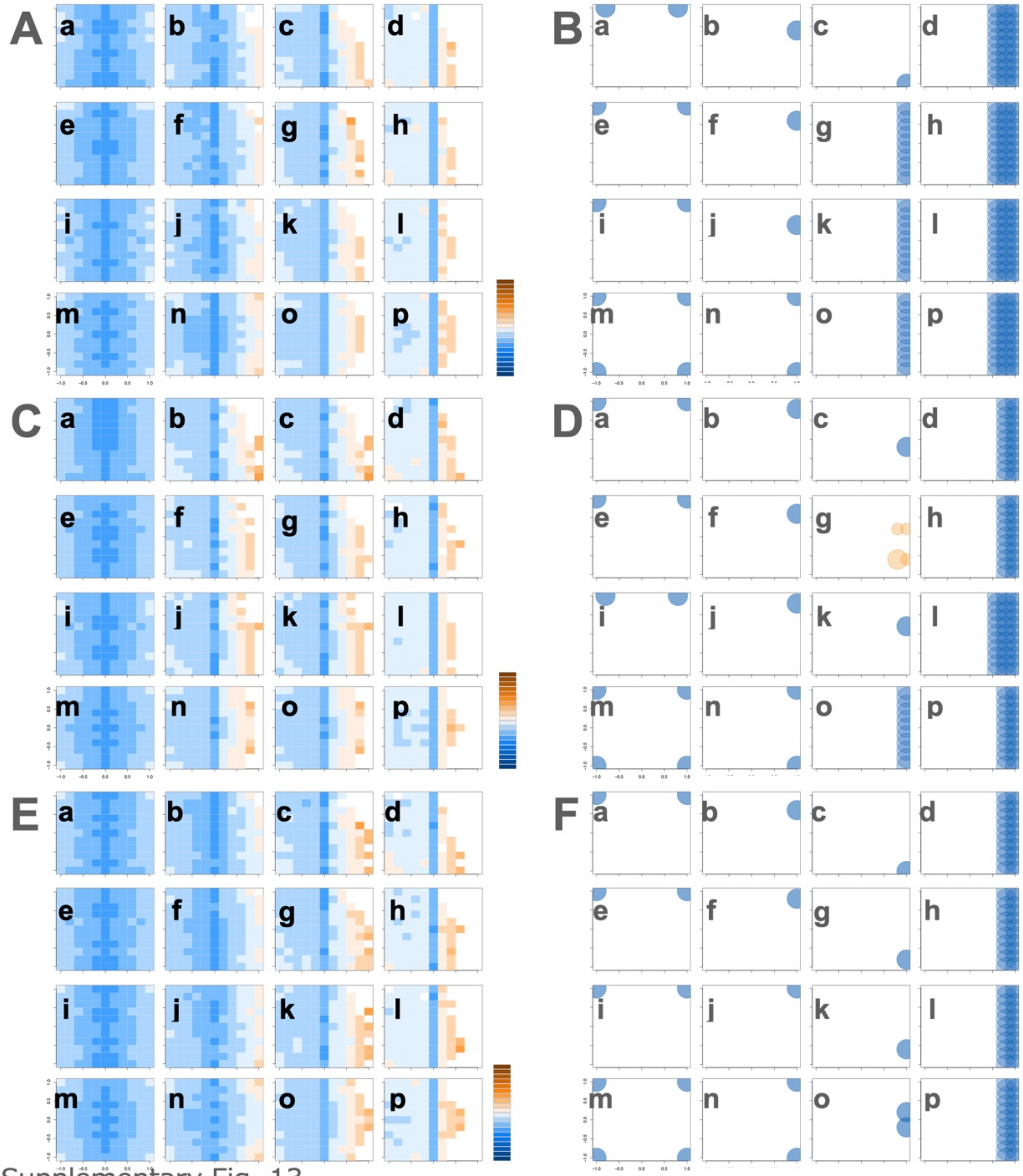

Supplementary Fig. 13

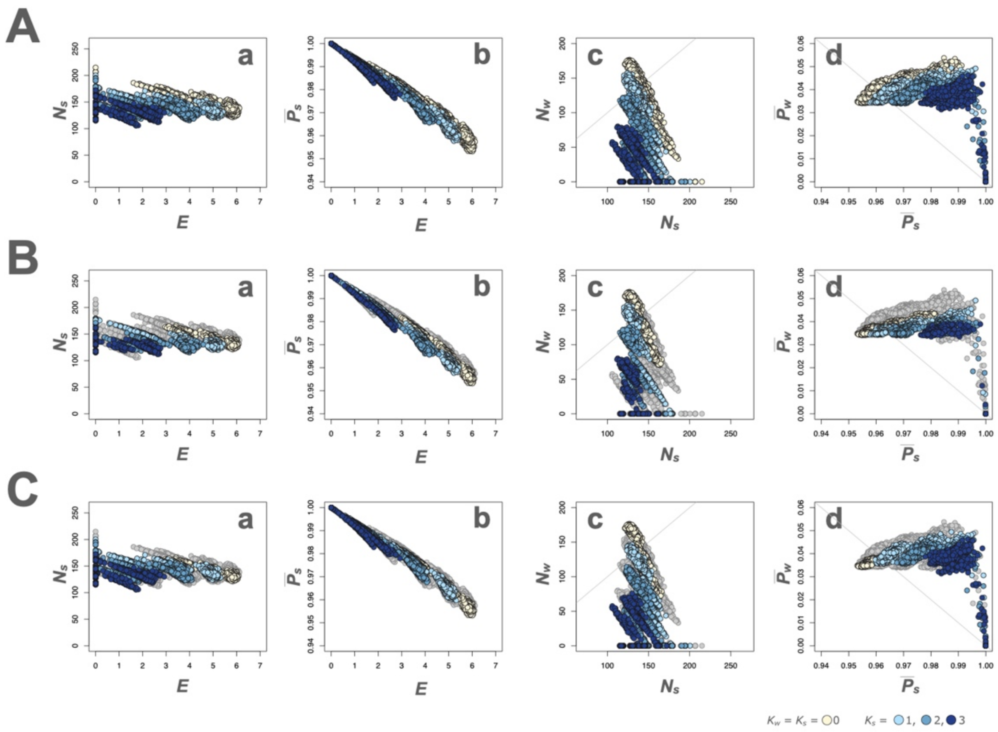

Supplementary Fig. 14

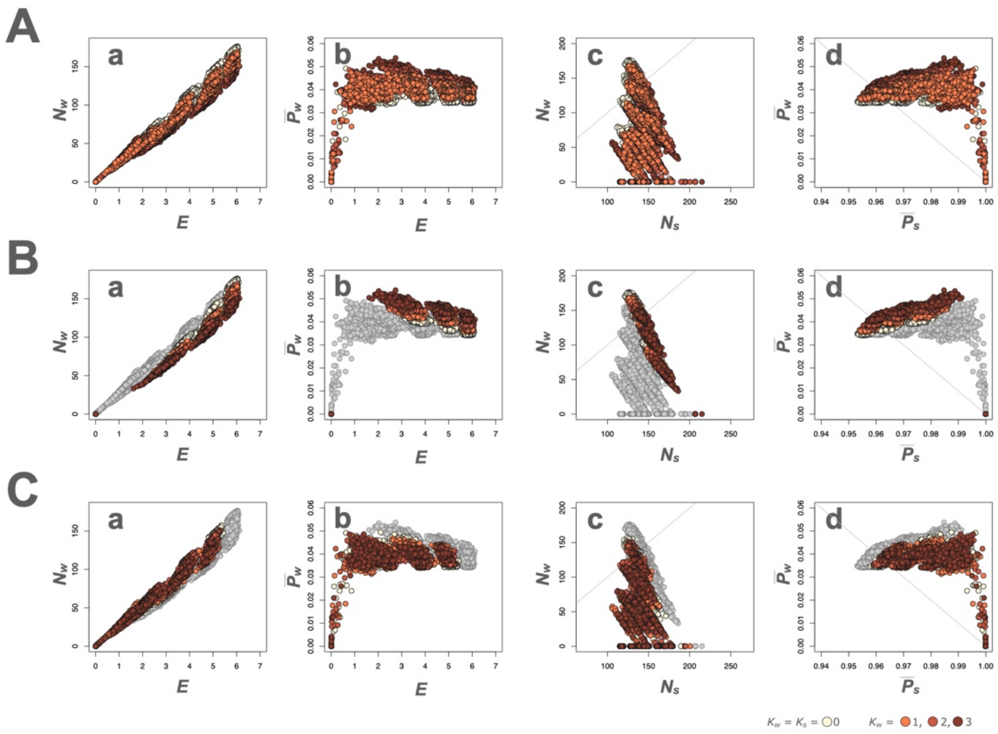

Supplementary Fig. 15

Supplementary Figure legends

Supplementary Fig. 1: Attack and defense behaviors between one predator and one prey with the sensory-motor algorithm.

A Attack and defense behaviors between one predator and prey (agents) in the grid world. **t1** (time step 1), **t2** (time step 2), and **t3** (time step 3) indicate the time sequences. **t1**, As an example of the agent positions, a predator (wolf) and prey (sheep) are shown in the grid world. Dotted lines indicate the ranges in which each agent detects the opponent (red dotted line for wolf: $D_w = 2$; blue dotted line for sheep: $D_s = 2$). When no opponent is within this range, the agent moves one grid at a time step (default speed = 1/time step). The shaded grids indicate where the agents can take their positions at the next time step (red shading for a wolf and blue shading for sheep). **t2**: The wolf and sheep detect the others within this range. When they detect their opponent, they change their speed (amount of speed change, S_i) (i= w or s). **Aa**, A case when the wolf chases and the sheep escapes ($S_w = 1$, $S_s = 1$). At **t2**, the speed of the wolf and sheep change to $1 + S_w = 2$ and $1 + S_s = 2$, respectively. The shaded grids indicate possible positions where they move. They encounter if they take the same position (arrows: **Aa-t2**). **Aa-t3**, The wolf eats the sheep. **Ab**, A case when the wolf ambushes and the sheep escapes ($S_w = -1$, $S_s = 1$). At **t2**, the speed of the wolf and sheep change to 0 and 2, respectively. The shaded grids indicate possible positions where they are located. They encounter if they take the same position (arrow: **Ab-t2**). **Ab-t3**, The wolf eats the sheep. **Ac**, A case when the wolf ambushes and the sheep freezes ($S_w = -1$, $S_s = -1$). At **t2**, the speeds of the wolf and sheep change to 0 and 0, respectively. Both remain in place and do not encounter each other. **Ad**, A case when the wolf chases and the sheep freezes ($S_w = 1$, $S_s = -1$). At **t2**, the speed of the wolf and sheep change to 2 and 0, respectively. The shaded grids indicate possible positions where they are located. They encounter if they take the same position (arrow: **Ad-t2**). **Ad-t3**, the wolf eats sheep.

Supplementary Fig. 2: The landscapes of payoff differences (P_{diff}) and the Nash equilibrium speed changes ($\bar{S}_i$) with the sensory-motor algorithm in the absence of the behavioral cost coefficient ($c_b = 0$).

A Landscapes of P_{diff}. Sixteen landscapes represent combinations of D_i (**a** to **p**). P_{diff} are color-coded. **B** $\bar{S}_i$ are shown under the small reproduction coefficient of predators (r = 3.2). The circle positions on the x- and y-axes indicate $\bar{S}_s$ and $\bar{S}_w$, respectively, at the same coordinates as the encounter probability landscape (**a** to **i**). The circle diameter represents the occurrence probability of behavior ($\bar{o}_i$). The blue circles indicate cases in which the Nash equilibrium behavior is uniquely determined with a diameter of 1. Orange

circles indicate the case where the agents probabilistically perform the behaviors whose diameters are proportional to $\bar{o}_i$ ($0 < \bar{o}_i < 1$).
C, **D** and **E**, **F**, Those are shown under the middle and large reproduction coefficient of predators ($r = 3.6$ and 4.0), respectively.

Supplementary Fig. 3: The landscapes of P_{diff} and $\bar{S}_i$ with the sensory-motor algorithm under the small behavioral cost coefficient ($c_b = 0.001$).
A Landscapes of P_{diff}. **B** $\bar{S}_i$ are shown under the small reproduction coefficient of predators ($r = 3.2$). **C**, **D** and **E**, **F**, Those are shown under the middle and large reproduction coefficient of predators ($r = 3.6$ and 4.0), respectively.

Supplementary Fig. 4: The landscapes of P_{diff} and $\bar{S}_i$ with the sensory-motor algorithm under the middle behavioral cost coefficient ($c_b = 0.02$).
A Landscapes of P_{diff}. **B** $\bar{S}_i$ are shown under the small reproduction coefficient of predators ($r = 3.2$). **C**, **D** and **E**, **F**, Those are shown under the middle and large reproduction coefficient of predators ($r = 3.6$ and 4.0), respectively.

Supplementary Fig. 5: The landscapes of P_{diff} and $\bar{S}_i$ with the sensory-motor algorithm under the large behavioral cost coefficient ($c_b = 0.04$).
A Landscapes of P_{diff}. **B** $\bar{S}_i$ are shown under the small reproduction coefficient of predators ($r = 3.2$). **C**, **D** and **E**, **F**, Those are shown under the middle and large reproduction coefficient of predators ($r = 3.6$ and 4.0), respectively.

Supplementary Fig. 6: Effects of D_s on encounter frequency (E), agent numbers (N_i), and payoffs ($\bar{P}_i$) when the agents engaged in Nash equilibrium with the sensory-motor algorithm under all conditions ($c_b = 0, 0.001, 0.02, 0.04$, and $r = 3.2, 3.6, 4.0$).
A All data are scatter-plotted as D_s increases; beige points represent $D_s = 0$, light blue points represent $D_s = 1$, blue points represent $D_s = 2$, and dark blue points represent $D_s = 3$ across all D_w values ($D_w = 0, 1, 2$, or 3).
B Points where $D_w = 0$ are colored according to increasing D_s. All other points ($D_w \neq 0$) are colored in gray.
C Points where $D_w \neq 0$ are colored according to increasing D_s. All other points ($D_w = 0$) are colored in gray.
Aa, **Ba**, and **Ca** Scatter plots with E on the x-axis and N_s on the y-axis show that E is smaller when D_s is larger, regardless of D_w (dark blue points shift to the left side), indicating that E decreases as D_s increases.

Ab, **Bb**, and **Cb** Scatter plots with E on the x-axis and $\bar{P}_s$ on the y-axis show that E is smaller and $\bar{P}_s$ is larger when D_s is larger, regardless of D_w (dark blue points shift to the upper-left side), indicating that E decreases and $\bar{P}_s$ increases as D_s increases.
Ac, **Bc**, and **Cc** Scatter plots with N_s on the x-axis and N_w on the y-axis show that N_w is smaller when D_s is larger, regardless of D_w (dark blue points shift to the lower side), indicating that N_w decreases as D_s increases.
Ad, **Bd**, and **Cd** Scatter plots with $\bar{P}_s$ on the x-axis and $\bar{P}_w$ on the y-axis show that $\bar{P}_s$ is larger when D_s is larger, regardless of D_w (dark blue points shift to the right side), indicating that $\bar{P}_s$ increases as D_s increases.

Supplementary Fig. 7: Effects of D_w on E, N_i, and $\bar{P}_i$ when the agents engaged in Nash equilibrium with the sensory-motor algorithm under all conditions (c_b = 0, 0.001, 0.02, 0.04, and r = 3.2, 3.6, 4.0).
A All data are scatter-plotted as D_w increases; beige points represent $D_w = 0$, orange points represent $D_w = 1$, brown points represent $D_w = 2$, and dark brown points represent $D_w = 3$ across all D_s values (D_s = 0, 1, 2, or 3).
B Points where $D_s = 0$ are colored according to increasing D_w. All other points ($D_s \neq 0$) are colored in gray.
C Points where $D_s \neq 0$ are colored according to increasing D_w. All other points ($D_s = 0$) are colored in gray.
Aa, **Ba**, and **Ca** Scatter plots with E on the x-axis and N_w on the y-axis. N_w is smaller when D_w is larger, where $D_s = 0$ (dark brown points shift to the lower side in **Ba**), indicating that N_w decreases as D_w increases.
Ab, **Bb**, and **Cb** Scatter plots with E on the x-axis and $\bar{P}_w$ on the y-axis. $\bar{P}_w$ is larger when D_w is larger, where $D_s = 0$ (dark brown points shift to the upper side in **Bb**), indicating that $\bar{P}_w$ increases as D_w increases.
Ac, **Bc**, and **Cc** Scatter plots with N_s on the x-axis and N_w on the y-axis. N_s is larger and N_w is smaller when D_w is larger, where $D_s = 0$ (dark brown points shift to the lower-right side in **Bc**), indicating that N_s increases and N_w decreases as D_w increases.
Ad, **Bd**, and **Cd** Scatter plots with $\bar{P}_s$ on the x-axis and $\bar{P}_w$ on the y-axis. $\bar{P}_s$ and $\bar{P}_w$ are larger when D_w is larger, where $D_s = 0$ (dark brown points shift to the upper-right side in **Bd**), indicating that $\bar{P}_s$ and $\bar{P}_w$ increase as D_w increases.

Supplementary Fig. 8: $\bar{P}_i$ with the sensory-motor algorithm between one predator and one prey.
$\bar{P}_s$ and $\bar{P}_w$ are scatter-plotted on the x-axis and the y-axis, respectively.

a, **b**, **c** Effects of D_w on $\bar{P}_i$ are shown; gray points represent D_w = 0, orange points represent D_w = 1, and brown points represent D_w = 2 when D_s is held constant (D_s = 0, 1, and 2, in **a**, **b**, and **c**, respectively).
d, **e**, **f** Effects of D_s on $\bar{P}_i$ are shown; gray points represent D_s = 0, light blue points represent D_s = 1, and blue points represent D_s = 2 when D_w is held constant (D_w = 0, 1, and 2, in **d**, **e**, and **f**, respectively).
g Effects of D_w and D_s on $\bar{P}_i$ are shown; gray points represent $D_w = D_s = 0$, light green points represent $D_w = D_s = 1$, and dark green points represent $D_w = D_s = 2$.
Because a game between one predator and one prey is a constant-sum game, all plots are on the diagonal line ($\bar{P}_\mathrm{s} + \bar{P}_\mathrm{w} = 1$). In **b**, **c**, and **f**, the points' sizes are modified to make their overlaps visible.

Supplementary Fig. 9: The partial behavioral switching.
We observed two types of probabilistic increasing and decreasing speeds in the Nash equilibrium behaviors. One is that both the predators and prey probabilistically switched, i.e., behavioral switching. The other is that one of them probabilistically switched their behaviors, i.e., partial behavioral switching.
A The behavioral switching and partial behavioral switching under the influences of the behavioral cost coefficient (c_b) and the reproduction coefficient of predators (r). $\bar{S}_i$ are indicated when both D_w and D_s are 2. The upper (**Aa**, **b**, **c**, and **d**), middle (**Ae**, **f**, **g**, and **h**), and lower rows (**Ai**, **j**, **k**, and **l**) indicate $\bar{S}_i$ under the large (r = 4.0), the middle (r = 3.6), and the small reproduction coefficient of predators (r = 3.2), respectively. The left (**Aa**, **e**, and **i**), the middle left (**Ab**, **f**, and **j**), the middle right (**Ac**, **g**, and **k**), and the right columns (**Ad**, **h**, and **l**) indicate $\bar{S}_i$ under the absence (c_b = 0), the small (c_b = 0.001), the middle (c_b = 0.02), and the large behavioral cost coefficient (c_b = 0.04), respectively. Circle positions on the x- and y-axes indicate $\bar{S}_s$ and $\bar{S}_w$, respectively, at the same coordinates as the payoff landscape. Orange circles indicate the cases where the predator and prey probabilistically perform the behaviors with increasing and decreasing speeds. The diameters of the orange circles are proportional to $\bar{o}_i$ ($0 < \bar{o}_i < 1$). Both the agents probabilistically increase or decrease their speeds in **Aa**, **b**, **c**, **f**, **g**, **j**. Only the prey increase or decrease their speeds; escape and freeze, but the predators do not; i.e., the partial behavioral switching is observed in **Ae**. Blue circles indicate the cases where $\bar{S}_i$ is uniquely determined ($\bar{o}_i = 1$) in **Ad**, **h**, **i**, **k**, **l**.
B Percentages of the behavioral switching in one of the agents (type 1, the partial behavioral switching) and both the agents (type 2, the behavioral switching) are presented in all c_b and r.

Supplementary Fig. 10: The landscapes of $\overline{P}_{diff}$ and $\overline{S}_i$ with the single-element motor algorithm under the small behavioral cost coefficient (c_b = 0).

A Landscapes of P_{diff}. Sixteen landscapes represent combinations of K_i (**a** to **p**). P_{diff} are color-coded. **B** $\bar{S}_i$ are shown under the small reproduction coefficient of predators (r = 3.2). Positions of the circles indicate $\bar{S}_s$ and $\bar{S}_w$ on the x- and y-axes, respectively, at the same coordinates as the payoff landscape (**a** to **p**). **C**, **D** and **E**, **F**, Those are shown in the same manner as in **A**, **B** under the middle and large reproduction coefficient of predators (r = 3.6 and 4.0), respectively.

Supplementary Fig. 11: The landscapes of $\overline{P}_{diff}$ and $\overline{S}_i$ with the single-element motor algorithm under the small behavioral cost coefficient (c_b = 0.001).

A Landscapes of P_{diff}. **B** $\bar{S}_i$ are shown under the small reproduction coefficient of predators (r = 3.2) in the same manner as in Supplementary Fig. 9. **C**, **D** and **E**, **F**, Those are shown under the middle and large reproduction coefficient of predators (r = 3.6 and 4.0), respectively.

Supplementary Fig. 12: The landscapes of $\overline{P}_{diff}$ and $\overline{S}_i$ with the single-element motor algorithm under the middle behavioral cost coefficient (c_b = 0.02).

A Landscapes of P_{diff}. **B** $\bar{S}_i$ are shown under the small reproduction coefficient of predators (r = 3.2) in the same manner as in Supplementary Fig. 9. **C**, **D** and **E**, **F**, Those are shown under the middle and large reproduction coefficient of predators (r = 3.6 and 4.0), respectively.

Supplementary Fig. 13: The landscapes of $\overline{P}_{diff}$ and $\overline{S}_i$ with the single-element motor algorithm under the large behavioral cost coefficient (c_b = 0.04).

A Landscapes of P_{diff}. **B** $\bar{S}_i$ are shown under the small reproduction coefficient of predators (r = 3.2) in the same manner as in Supplementary Fig. 9. **C**, **D** and **E**, **F**, Those are shown under the middle and large reproduction coefficient of predators (r = 3.6 and 4.0), respectively.

Supplementary Fig. 14: Effects of K_s on E, N_i, and $\overline{P}_i$ when the agents engaged in Nash equilibrium with the single-element motor algorithm under all conditions (c_b = 0, 0.001, 0.02, 0.04, and r = 3.2, 3.6, 4.0).

A All data are scatter-plotted as K_s increases; beige points represent K_s = 0, light blue points represent K_s = 0.1, blue points represent K_s = 0.2, and dark blue points represent

K_s = 0.3 across all K_w values (K_w = 0, 0.1, 0.2, or 0.3).

B Points where $K_w = 0$ are colored according to increasing K_s. All other points ($K_w \neq 0$) are colored in gray.

C Points where $K_w \neq 0$ are colored according to increasing K_s. All other points ($K_w = 0$) are colored in gray.

Aa, **Ba**, and **Ca** Scatter plots with E on the x-axis and N_s on the y-axis show that E is smaller when K_s is larger, regardless of K_w (dark blue points shift to the left side), indicating that E decreases as K_s increases.

Ab, **Bb**, and **Cb** Scatter plots with E on the x-axis and $\bar{P}_s$ on the y-axis show that E is smaller and $\bar{P}_s$ is larger when K_s is larger, regardless of K_w (dark blue points shift to the upper-left side), indicating that E decreases and $\bar{P}_s$ increases as K_s increases.

Ac, **Bc**, and **Cc** Scatter plots with N_s on the x-axis and N_w on the y-axis show that N_w is smaller when K_s is larger, regardless of K_w (dark blue points shift to the lower side), indicating that N_w decreases as K_s increases.

Ad, **Bd**, and **Cd** Scatter plots with $\bar{P}_s$ on the x-axis and $\bar{P}_w$ on the y-axis show that $\bar{P}_s$ is larger when K_s is larger, regardless of K_w (dark blue points shift to the right side), indicating that $\bar{P}_s$ increases as K_s increases.

Supplementary Fig. 15: Effects of K_w on E, N_i, and $\bar{P}_i$ when the agents engaged in Nash equilibrium with the single-element motor algorithm under all conditions (c_b = 0, 0.001, 0.02, 0.04, and r = 3.2, 3.6, 4.0).

A All data are scatter-plotted as K_w increases; beige points represent $K_w = 0$, orange points represent $K_w = 0.1$, brown points represent $K_w = 0.2$, and dark brown points represent K_w = 0.3 across all K_s values (K_s = 0, 0.1, 0.2, or 0.3).

B Points where $K_s = 0$ are colored according to increasing K_w. All other points ($K_s \neq 0$) are colored in gray.

C Points where $K_s \neq 0$ are colored according to increasing K_w. All other points ($K_s = 0$) are colored in gray.

Aa, **Ba**, and **Ca** Scatter plots with E on the x-axis and N_w on the y-axis. N_w is smaller when K_w is larger, where $K_s = 0$ (dark brown points shift to the lower side in **Ba**), indicating that N_w decreases as K_w increases.

Ab, **Bb**, and **Cb** Scatter plots with E on the x-axis and $\bar{P}_w$ on the y-axis. $\bar{P}_w$ is larger when K_w is larger, where $K_s = 0$ (dark brown points shift to the upper side in **Bb**), indicating that $\bar{P}_w$ increases as K_w increases.

Ac, **Bc**, and **Cc** Scatter plots with N_s on the x-axis and N_w on the y-axis. N_s is larger and N_w is smaller when K_w is larger, where $K_s = 0$ (dark brown points shift to the lower-right

side in **Bc**), indicating that N_s increases and N_w decreases as K_w increases.

Ad, **Bd**, and **Cd** Scatter plots with $\bar{P}_s$ on the x-axis and $\bar{P}_w$ on the y-axis. $\bar{P}_s$ and $\bar{P}_w$ are larger when K_w is larger, where $K_s = 0$ (dark brown points shift to the upper-right side in **Bd**), indicating that $\bar{P}_s$ and $\bar{P}_w$ increase as K_w increases.
