# Supplementary material for: Nash equilibrium of attack and defense behaviors between predators and prey
Source: PLoS Comput Biol. 2025 Nov 21;21(11):e1013730. doi: 10.1371/journal.pcbi.1013730 (PMC12671891; doi:10.1371/journal.pcbi.1013730)
Supplement: Attachment — Submitted filename: report-PLoSCompBiol-D-24-01911-R1.pdf [file pcbi.1013730.s044.pdf]

Report – Revised version - PCOMBIOL-D-24-01911

The authors performed significant revisions of the manuscript addressing the comments from both reviewers. With these changes, some important issues have been clarified, and I consider the manuscript potentially suitable for *PLOS Computational Biology*. While the manuscript has improved substantially, there are still some issues that should be addressed.

Major comment:

First, the authors did not fully address my concerns—nor those raised by the other reviewer—regarding the game-theoretic treatment of the multi-player game and the potential confusion between classical game theory, evolutionary game theory, and the relationship between Nash equilibria and Evolutionarily Stable Strategies (ESS). The additional explanations partly respond to our comments, but I believe further clarification specific to this model would be beneficial.

For example, the authors treat E as an intermediate variable that is influenced by predator and prey behaviors. While this is true, E is still a **population-level** variable, and it would help to explain more clearly under what conditions or assumptions it is reasonable to treat E as uniform across all agents. In other words, what assumptions underlie this approach? It appears the model implicitly assumes a homogeneous population of agents interacting in a well-mixed scenario. I would suggest the authors make this assumption explicit.

Additionally, I remain unconvinced by the current discussion contrasting short-term outcomes (which the authors associate with Nash equilibria) and the "long-term fitness landscape." The current explanation still seems incomplete.

For a Nash equilibrium, no player can improve their payoff by unilaterally changing their strategy, assuming all other strategies are fixed. In contrast, for an ESS, one must show that a strategy adopted by the population cannot be invaded by any mutant strategy. While reproduction plays a key role, what is ultimately crucial is the **relative fitness** of two competing strategy phenotypes, assuming fitness-proportionate reproduction. If convincing arguments could be made about the relative fitness of prey strategies, one could potentially argue that the Nash equilibria also correspond to ESS for the prey—or not.

A particularly useful direction would be to consider the **adaptive dynamics limit** of rare mutants in a stationary population of prey agents playing the Nash equilibrium strategy. In this scenario, the encounter rate for the rare mutant (e.g., a single mutant in a resident NE population) should depend only on the number of predators and both the predator (Nash) strategies and the prey mutant strategy. If it can be shown that the payoffs for mutant strategies are always lower than those of the resident strategy, one could conclude that the NE corresponds to an ESS. If not, this would suggest the ESS differs from the NE.

My suspicion is that, if the **sensory range is fixed**, then the "motor" Nash strategies likely correspond to ESS. However, if **detection range becomes an evolvable trait** (i.e., mutants with different sensory ranges are possible), and if the costs of larger sensory range are not too high, then mutants with larger D may out-compete the resident NE with smaller D.

Given that both reviewers have raised concerns about the conceptual distinction between classical and evolutionary game theory, I suggest that the authors look more deeply into this issue and consider addressing it further—perhaps along the lines outlined above.

Further minor comments:

- **Abstract, line 68**:
  "... and establish their behaviors at Nash equilibria."
  → Not clear. How can agents establish a behavior at Nash equilibria? For me, a behavior can correspond to a Nash equilibrium.

- **Abstract, line 71**:
  "...were the non-constant sum and positive sum in most cases."
  → Needs revision. Suggest: *"...were, in most cases, non-constant-sum and positive-sum games."*

- **Abstract, line 79**:
  "...allows agents to compute their payoffs."
  → Unclear. What is meant by this? It's not evident how this would work in a real-world setting, especially as it seems to require perfect knowledge of the opponent's behavior.

- **Line 137**:
  → Change to: *"...increase payoffs by changing their behaviors."*

- **Line 145**:
  The sentence is potentially misleading:
  "This study addresses this challenge and reveals a game-theoretic solution for the Nash equilibrium in the interaction between attack and defense."
  → This wording implies that an analytical solution was derived, which is not the case. I recommend: *"This study addresses this challenge and identifies Nash equilibria for the interaction between attack and defense using numerical simulations."*

- **Line 224**:
  Some strategies seem biologically implausible, likely due to simplifying assumptions—e.g., that neither predator nor prey can detect relative positions or motion. For instance, for a prey with a greater detection distance than the predator, the "freeze" strategy appears unrealistic. If the prey detects a predator at a distance and has an information advantage, the optimal response would typically be to flee (move directly away from the predator). The freeze strategy may only be advantageous when the predator is already close, has a similar or larger detection range, and can outrun the prey. By freezing the prey could reduce the risk of detection. While I do not see this as a fundamental flaw, I would expect a discussion on how access to directional information could qualitatively alter the resulting Nash equilibria.

- **Lines 246–249**:
  On rereading, I remain unclear about the difference between D_i and d_i, and the role of the factor f. Is d_i used only in the cost-benefit calculation? Given D_i=d_i*f and f=10, with D_i ranging up to 3, d_i must be at most 0.3. This appears a bit arbitrary. Why not equate D_i directly to the trait?

- **Figure 2E**:
  The choice of color scale is problematic—predator payoff values occupy only a narrow lower range, making it hard to see any structure in the heatmap. I assume this is to enable direct comparison across panels, but since payoffs are in arbitrary units and predator/prey values likely differ in scale, strict comparability seems unnecessary. Including numerical

labels on the colorbar would help clarify the magnitude of variation. As it stands, the heatmaps might span only a tiny range (e.g., 1 to 1.001), which is hard to assess.

- **Figures (general)**:
  The figures appear to be of low resolution, which makes reading small font sizes difficult.
