# Supplementary material for: Nash equilibrium of attack and defense behaviors between predators and prey
Source: PLoS Comput Biol. 2025 Nov 21;21(11):e1013730. doi: 10.1371/journal.pcbi.1013730 (PMC12671891; doi:10.1371/journal.pcbi.1013730)
Supplement: Attachment — Submitted filename: the response from the editor (PBIOLOGY-D-24-03015).docx [file pcbi.1013730.s039.docx]

Dear Editor,

Below is the response from the editor regarding my submission (PBIOLOGY-D-24-03015). As stated, the manuscript did not proceed to the reviewer stage as a result of the editorial assessment. The content of the submitted manuscript remains unchanged from PBIOLOGY-D-24-03015.

Thank you for your consideration.

Sincerely,

Hiroyuki Ichijo

##########

件名: Your PLOS Biology Submission (PBIOLOGY-D-24-03015) - [EMID:728894b91dca863c]

送信者: "Roland G Roberts" <em@editorialmanager.com>

宛先: "Hiroyuki Ichijo" <ichijo@med.u-toyama.ac.jp>

送信日時: 2024年10月24日(木) 20:04:38

Dear Dr Ichijo,

Thank you for submitting your manuscript entitled "Nash equilibrium of attack and defense behaviors between predators and prey" to PLOS Biology for consideration. I regret to inform you that after careful discussion within the editorial team, we have decided that we cannot consider it for publication.

We were interested to read about your use of simulation to explore the algorithmic requirements to recover a Nash equilibrium between predators and prey, and we appreciate the potential implications of your findings for our ability to understand the underlying neuronal processes underlying predation and evasion. Regretfully, however, we do not feel that your study represents the strength of advance that we require for publication in PLOS Biology.

As you may know, we decline a substantial proportion of manuscripts without sending them to reviewers, so that they may be sent elsewhere without delay. In such cases, even if reviewers were to certify the manuscript as technically correct, we consider that the work does not represent the type of advance that PLOS Biology seeks to publish. This editorial assessment is based on considerations such as the degree of advance provided and the breadth of potential interest of the research question to our broad readership.

While we cannot consider your manuscript for publication in PLOS Biology, we suggest that you consider transferring it to PLOS Computational Biology. The PLOS journals are editorially independent, so we cannot guarantee it will be reviewed there.

If you would like to transfer your manuscript, as suggested, please click the following link:

https://www.editorialmanager.com/pbiology/l.asp?i=1104061&l=0IJFTAM1

If you do NOT wish to transfer your manuscript, please click this link to decline:

https://www.editorialmanager.com/pbiology/l.asp?i=1104062&l=02D20BHR

Please note, you can log into the submission sites with the same login that you used to submit to this journal.

Should you choose to transfer your submission you will receive a confirmation email within 24-48 hours after accepting the transfer. If you have any questions, please feel free to contact the journal at plosbiology@plos.org.

Thank you again for your interest in PLOS Biology. I am sorry that we cannot be more positive on this occasion. Please be assured that this editorial decision does not represent a criticism of the quality of your work, nor are we questioning its value to others working in this area. I hope you will consider PLOS Biology for other submissions in the future. Thank you for your support of PLOS and of Open Access publishing.

Sincerely,

Roli Roberts

Roland Roberts, PhD

Senior Editor

PLOS Biology

rroberts@plos.org
