# Supplementary material for: Nash equilibrium of attack and defense behaviors between predators and prey
Source: PLoS Comput Biol. 2025 Nov 21;21(11):e1013730. doi: 10.1371/journal.pcbi.1013730 (PMC12671891; doi:10.1371/journal.pcbi.1013730)
Supplement: Attachment — Submitted filename: PCOMPBIOL-D-24-01911_review.docx [file pcbi.1013730.s040.docx]

**Review of “Nash equilibrium of attack and defense behaviors between predators and prey”**

This paper develops models to analyse the outcome of predator-prey interactions under different strategy-use by the predators and prey. In particular, the strategies are given by (i) how far away the predator and prey can sense one another, and (ii) how they change their movement speed once a detection has been made. The authors use lattice simulations to compute, for each strategy, encounter probabilities between the predator and prey, and use these probabilities as payoffs in a matrix game. The Nash equilibria of the resulting game are then calculated and compared across different parameter combinations.

I found the premise of the paper quite interesting. The notion of computing game payoffs from an explicit simulation of the population dynamics is quite neat, and it is an interesting way to approach this question. I also appreciated the addition of the files accompanying the supplementary data that show how the data is structured. However, the paper is in general quite hard to read and, in my opinion, the results require more discussion and explanation. I therefore have some questions and suggestions for the authors. I also have a few conceptual questions that I had trouble thinking through. I should say first that I do not have expertise in the neuroethology of predator-prey interactions, so I cannot comment on the novelty of these results in that field. But I do have a background in theoretical ecology and evolution, so my comments will mostly be stated from that perspective.

**Major comments:**

- I found the abstract and introduction quite vague in stating the precise research question with this model: in hindsight of course it can be inferred, but it would be good to state this in the beginning more explicitly. I am thinking of something like “how should animals respond to predators/prey when they can sense far away vs. not at all?”. But this is only a suggestion of course. It is also unclear sometimes what the state of the literature is, in particular what is known from a modelling perspective and how this paper is adding to this body of work (is this the first paper to make this kind of model?). The references 28-30, 32, 34, are cited by the authors as relevant, so a short summary of at least these papers’ results would help the reader put this work in context. The ending of the abstract is also extremely vague – what does it mean for evolutionary mechanisms to be intrinsic to the model environments?

- a more conceptual question: in the multiple predators-multiple prey results, why are the payoffs P_s and P_w defined the way they are? This is never justified, and is counterintuitive since I would expect that the functional forms in the definition are symmetric i.e. they look the same for predator and prey. In particular, one can also define the payoffs as

P_w = E/(E+N_w)	and

P_s = 1- E/E+N_s

And it is not justified why this is not the choice that is made. In my opinion, the quantity E is not, as the authors state, “number of encounters at each time point (encounter frequency, *E*)” (L456). If it was, the total number of predators on the lattice at time t would be (hungry wolves + E) and the total number of prey would be (uneaten sheep + E) because each encounter requires a wolf and a sheep. Their quantity E is instead, if I am correct, something like the number of predators that encounter a prey at that timestep. Perhaps my confusion will be solved if the authors define what they mean by an “encounter”. But in any case, a justification for the above choice would be nice, as well as a demonstration that the results don’t change qualitatively if one defines the encounter probability as above.

- I am not sure how to interpret the game that is being considered here, in three ways:

(i) Games are usually models of the *outcome* of interactions between different individuals. However, here, the payoff is not representing an outcome but a likelihood of the interaction taking place at all. This is relevant because I (and I think many people) usually envision agents changing their strategies based on the outcome, but here it is hard to imagine what is making agents change strategies.

(ii) Related to this, the game considered describes neither a learning process (the prey cannot learn from a failure, it just dies) nor completely an evolutionary process (the predator can learn). The authors clearly realise this (during the discussion, L379), but don’t really offer the reader a way to reconcile these difficulties. This is for sure a conceptual problem that I’d like the authors to address, in that there are different timescales at play here: the predator behaviour can change really quickly but the prey behaviour changes slower.

(iii) But that is a bit philosophical. More proximately: A Nash equilibrium can be reached only if the players have full knowledge of the game payoffs, and I cannot see how the predators and prey can estimate the encounter probability for any pair of strategies, including for that matter the one that they currently possess. And roughly stated, if players cannot estimate any of the payoff entries, we are in the realm of evolutionary game theory. I would suggest therefore that the authors do one of two things: (i) compute the evolutionarily stable strategies instead of Nash equilibria, and derive all biological inferences from there, OR (ii) give a reason for why computing the ESS is not required, including an explicit description of how the Nash equilibrium is supposed to be reached over time by the predator and prey. If the overarching question is biological, i.e. “what should one expect the predator and prey strategies to be after a long time?”, the paper would benefit from an exact statement of how the results from this model answer that question. (P.S. It is known that all Nash equilibria need not be evolutionarily stable strategies (see e.g. the book of Broom and Rychtar).)

**Minor major comments:**

- the amount of information in the figures is really high. I appreciate that the authors have run extensive simulations, but it was very easy to get lost trying to go through all the subpanels. Speaking for myself, I still do not feel like I have a strong intuitive grasp on the results even after spending a good amount of time on the figures. Perhaps it is better to not make any changes to the figures themselves, but I would at least suggest that some changes be made to the narration so that it’s easier to get the intended message from the figures. The authors could structure their Results section in the following way: pick an example illustrative of the results and take the readers through it while building complexity and explaining the intuition along the way. Then state the bigger message that you want readers to take away from this. After this one can describe all the results more systematically like presently done. But even here, readers will appreciate if, for every sentence of the form “\bar{S}_i changes as D_i increases” (not quoting from the MS here, just meant to be an example sentence), there is a sentence immediately after this that states the same result in a biologically straightforward way. This will not only help the results to be accessible to a wider audience, but also guide the intuition of those who may be familiar with modelling.

- It is quite interesting that the Nash equilibrium is a pure strategy when D_i is low, but becomes a mixed strategy as D_i are both = 2. Why does this happen? It happens in both the single predator-prey case and multiple predator-prey case so it seems robust, but is it a general pattern that if the predator and prey can sense each other from quite far away, mixed strategies are the expected outcome? And why doesn’t it happen when D_i are equal but lower, e.g. both =1?

- It is clever to do the stochastic speed change strategy as a “control” of sorts. But

(i) I do not understand the authors’ claim here and elsewhere that the single element algorithm is “sufficient to achieve a Nash equilibrium”. The existence of a Nash equilibrium is always guaranteed when the set of strategies is finite, so it should not at all be surprising that there exists a Nash equilibrium for any of these games. And what does it mean to “achieve” a Nash equilibrium when the actual learning/evolutionary dynamics is not explicitly modelled? The comparison that is possible here is between the Nash equilibria under sensory-motor strategies and the stochastic speed change strategies. For example it would be cool to have more information on whether the stochastic speed change strategies can ever lead to the *same* Nash equilibrium (in terms of payoffs and population sizes) as the sensory-motor algorithm.

(ii) In L312+ the results show that these Nash equilibria are in general different. I would like a bit more intuition here too, e.g. why is the effect of the single element algorithm different on the predator and prey? It decreases N_w but increases N_s. And also predators went extinct under sensory-motor but never prey. These asymmetries are fundamental to the results so would be nice if they are given clearer explanations.

- Encounter probabilities with one predator and one prey: since there are a finite number of grid cells, is it not guaranteed that the predator will eventually be on the same grid cell as the prey, irrespective of all aspects of the strategy? Why is there variation then in the encounter probability? It would help a lot here if the authors detail how this probability is calculated and not just point the reader to the script they have written (L455).

**Minor comments:**

**L201 “constant sum game only when….both agents were the same”.** I see how P_s + P_w = 1 when E is zero. But the other claim is that P_s + P_w = 1 when N_s=N_w and I don’t think this is always true because if we set N_s=N_w=some number k, then P_s + P_w = 1 - E/(E+k) + E/k = k/(E+k) + E/k which is not necessarily 1. It adds up to [k^2 + E(E+k)]/[k(E+k)] unless I’m making an embarrassing mistake :)

-When individuals change their speed post detecting something, which direction do they go in? The answer to this is likely that they do not change direction, but then I would ask how the direction of movement decided at all, both before and after something is detected within the sensory radius. This is, as far as I can tell, not described in the methods section.

- It would also become much easier to get a grasp of the model if the authors provide movies visualising the simulations.

**L34 “Recent studies…defense behaviours.”** This sentence requires references to the recent studies.

**L37 “numerically evaluating the modifications...”** — modifications in what? This is extremely cryptic and actually took me quite a while to figure out, and my best guess now is that the authors mean numerically evaluating the modifications in encounter probabilities. This sentence should for sure be completed, and even then the notion of encounter probabilities has not been introduced yet at that point.

**L67 “the single-element motor and sensory-motor…respectively”.** This sentence is really quite vague — I am not sure what the difference is between a “minimal” component and a “fundamental” component, and how do I distinguish between a function of attack/defense and something that is only comparable to attack/defense?

- I don’t think the figures are all vector images (e.g. pdf) but might be wrong on this. Since the figures demand a lot of zooming, it would be good if they did not lose resolution when zoomed into.
