# Supplementary material for: Nash equilibrium of attack and defense behaviors between predators and prey
Source: PLoS Comput Biol. 2025 Nov 21;21(11):e1013730. doi: 10.1371/journal.pcbi.1013730 (PMC12671891; doi:10.1371/journal.pcbi.1013730)
Supplement: Attachment — Submitted filename: Response to Reviewers.docx [file pcbi.1013730.s042.docx]

30 April 2025

Dear Editor and Reviewers,

We would like to express our heartfelt thanks to the reviewers for their thoughtful and constructive feedback on our manuscript, “Nash equilibrium of attack and defense behaviors between predators and prey.” We are sincerely grateful for the time and effort they devoted to evaluating our work. Their comments greatly helped us improve the clarity, depth, and overall quality of the manuscript.

In this revised version, we have addressed each point raised, made clarifications where needed, incorporated the reviewers’ suggestions and examples, and enhanced the writing throughout. A detailed, point-by-point response is provided in responses to reviewers below. Reviewer comments are shown in blue, with our responses following in black, and revised sentences in red. In the revised manuscript, newly added texts have been highlighted.

We truly appreciate the opportunity to revise our work and thank the editors and the reviewers once again for their valuable input and kind guidance.

Hiroyuki Ichijo

##########

Reviewer 1

Major comments:

- I found the abstract and introduction quite vague in stating the precise research question with this model: in hindsight of course it can be inferred, but it would be good to state this in the beginning more explicitly. I am thinking of something like “how should animals respond to predators/prey when they can sense far away vs. not at all?”. But this is only a suggestion of course. It is also unclear sometimes what the state of the literature is, in particular what is known from a modelling perspective and how this paper is adding to this body of work (is this the first paper to make this kind of model?). The references 28-30, 32, 34, are cited by the authors as relevant, so a short summary of at least these papers’ results would help the reader put this work in context. The ending of the abstract is also extremely vague – what does it mean for evolutionary mechanisms to be intrinsic to the model environments?

> We thank Reviewer 1 for his advice to improve the manuscript. Many sentences in the abstract, introduction, and discussion have been rewritten, taking care not to obscure. The changed wording has been highlighted. In response to the points made by Reviewer 1, we summarized the previous works as follows and stated what this study has added in the Introduction section.

>> line 126

“As proposed by Lima[22], this requires modeling the strategic behaviors of both predator and prey, allowing the mutual influence of attack and defense behaviors on their payoffs, based on the encounter probabilities, to be analyzed using game theory[22–28]. In line with this approach, previous studies have described predator-prey interaction as a two-player, noncooperative zero-sum game and analytically elucidated the outcomes[29–31]. Chen et al. (2005) modeled the predator’s attack (pursuit, ambush) and prey’s defense behaviors (escape, hide)[29]. Zoroa et al. (2011) formulated sit-and-wait and active predation behaviors for predators to search for food[30]. Alpern et al. (2019) focused on the search-pursuit interaction as a stochastic iterative game in which both agents move[31]. The solution to the game is defined by the Nash equilibrium[32]. The Nash equilibrium behaviors illustrate how agents engage in specific behavioral patterns and achieve stable payoffs, where neither the predators nor the prey increase their payoffs by replacing alternative behaviors[22,31,33,34]; however, previous studies have not determined the Nash equilibrium.”

- a more conceptual question: in the multiple predators-multiple prey results, why are the payoffs *P_s_* and *P_w_* defined the way they are? This is never justified, and is counterintuitive since I would expect that the functional forms in the definition are symmetric i.e. they look the same for predator and prey. In particular, one can also define the payoffs as

*P_w_* = *E*/(*E* + *N_w_*) and *P_s_* = 1- *E*/(*E* + *N_s_*).

And it is not justified why this is not the choice that is made. In my opinion, the quantity *E* is not, as the authors state, “number of encounters at each time point (encounter frequency, *E*)” (L456). If it was, the total number of predators on the lattice at time t would be (hungry wolves + *E*) and the total number of prey would be (uneaten sheep + *E*) because each encounter requires a wolf and a sheep. Their quantity *E* is instead, if I am correct, something like the number of predators that encounter a prey at that timestep. Perhaps my confusion will be solved if the authors define what they mean by an “encounter”. But in any case, a justification for the above choice would be nice, as well as a demonstration that the results don’t change qualitatively if one defines the encounter probability as above.

>We thank Reviewer 1 very much for the question about the definition of encounter. We apologize for the lack of an explicit definition at the beginning. Accordingly, we have added a definition of encounter at the beginning of the Results section as shown below.

>> line 193

“If both agents are located at the same grid at the final time step (a-t2), they encounter and predation occurs (t3). To simplify the analysis, we defined “encounter” as synonymous with predation.”

> We thank Reviewer 1 very much for the question about the formula for calculating encounter probabilities. We apologize that we did not explain the NetLogo program well enough. The confusion regarding encounter probabilities likely stems from the structure of the NetLogo program. The numbers of individuals and encounters are recorded at the end of the procedure, so the recorded prey population numbers do not include predated individuals. Therefore, *p_s_* = *E*/*N_s_* is not accurate; instead, it should be defined as *p_s_* = *E*/(*E* + *N_s_*), *E* must be divided by the number of individuals before predation occurs. On the other hand, the predator population includes predators that have preyed. Therefore *p_w_* = *E*/*N_w_* is the appropriate definition. This explanation has been added to both the Results and Methods sections.

>>line 263 (Results)

“In the NetLogo program, *E* and *N_i_* were recorded after agent interactions. The number of predators (*N_w_*) included both encountered and unencountered predators, whereas the number of prey (*N_s_*) excluded prey that were removed due to predation. Thus, predator and prey encounter probabilities (*p_w_* and *p_s_*) were calculated as *E*/*N_w_* and *E*/(*E* + *N_s_*), respectively. In general, predation consists of both pre- and post-encounter processes[35], but in the IBM, these processes were simplified by treating the encounter itself as predation.”

>> line 713 (Methods)

“As *E*, *N_w_*, and *N_s_* were reported at the end of the “to go” procedure after the agents interacted in the NetLogo program, *N_w_* included the number of wolves both with an encounter and no encounter; on the other hand, *N_s_* did not include the number of sheep died of predation with an encounter but included only with no encounter. Therefore, encounter probabilities of wolves (*p_w_*) and sheep (*p_s_*) were defined as *E*/*N_w_* and *E*/(*E* + *N_s_*), respectively. The formula appeared asymmetrical, but the encounter itself is symmetrical, with contributions from a sheep and a wolf.”

> Concerning “a demonstration that the results do not change qualitatively if one defines the encounter probability as above”, we did calculate the payoffs as *P_w_* = *E*/*N_w_* and *P_s_* = 1- *E*/*N_s_* and computed the Nash equilibria. We show this results below (Additional Fig. 1).

**Additional Fig. 1** The figure shows qualitatively similar results across conditions.

Panels **A**, **B**, and **C** represent the payoff landscapes of prey, predators, and the distribution of Nash equilibria, respectively, with the values of the behavioral cost coefficients (*c_b_* =0.02) and reproductive coefficients of the predators (*r* = 3.6). These indicate that the prey and predator landscapes, as well as the distribution of Nash equilibria, are similar to those shown in Fig. 2D–F of the manuscript.

Panel **D** represents scatter plots of expected payoffs of prey (x-axis) versus predator (y-axis) under different combinations of the agents’ detection distances with *c_b_* =0.02 and *r* = 3.6.

**Da** shows effects of predators’ detection distances when prey’s detection distance *D_s_* = 0. Orange, brown, and dark brown points correspond to predators’ detection distances *D_w_* = 1, 2, and 3, respectively. The result indicates that predator attacks increase both predator and prey payoffs when prey have no detection ability, similar to the results in Fig. 3Cd of the manuscript.

**Db** shows effects of prey’s detection distances when *D_w_* = 2. Gray, light blue, blue, and dark blue points correspond to *D_s_* = 0, 1, 2, and 3, respectively. The result indicates that prey defenses increase their own payoffs while reducing those of predators, similar to the results in Supplementary Fig. 6Cd of the manuscript. Beige points indicate the condition where *D_w_* = *D_s_* = 0.

Panel **E** presents bar plots showing the effect of behavioral cost coefficients (*c_b_*) on behavioral switching. The bars indicate the percentage of the behavioral switching in one of the agents (type 1, the partial behavioral switching) and both the agents (type 2, the behavioral switching) in all *c_b_* and reproductive coefficients of predators (*r*). Behavioral switching is more likely to emerge at low *c_b_* values, similar to the results in Supplementary Fig. 9B of the manuscript.

- I am not sure how to interpret the game that is being considered here, in three ways:

- (i) Games are usually models of the *outcome* of interactions between different individuals. However, here, the payoff is not representing an outcome but a likelihood of the interaction taking place at all. This is relevant because I (and I think many people) usually envision agents changing their strategies based on the outcome, but here it is hard to imagine what is making agents change strategies.

> We thank Reviewer 1 for raising the question regarding the definition of agents’ payoffs, and we apologize for the insufficient explanation in the original manuscript. In this study, we idealized certain aspects of predator–prey interactions. One such simplification is that we focus solely on the pre-encounter phase of interactions, omitting the post-encounter processes. As Reviewer 1 rightly pointed out, the encounter probability represents the likelihood of interaction. However, in our NetLogo simulation, predators gain energy as a behavioral outcome in proportion to their encounter probability, calculated as the product of the encounter probability and a constant parameter (wolf-gain-from-food). In contrast, prey lose all their energy (i.e., die) depending on the encounter probability, but gain energy from consuming grass (sheep-gain-from-food) when they successfully evade predation. Therefore, we thought that it is reasonable to define the agents’ payoffs based on encounter probability or its complement. We have added this explanation to the Methods section to clarify the rationale.

>> line 269 (Results)

“In this framework, predators gain energy in proportion to their encounter probability, and prey gain energy by eating grass after evading the encounter in the IBM (Supplementary Table 1). Accordingly, we defined agents’ payoffs (*P_i_*) based on their encounter probabilities: predator payoffs (*P_w_*) = *p_w_*, and prey payoffs (*P_s_*) = 1 − *p_s_*. Because *P_i_* fluctuated over time, we used their average values to represent behavioral outcomes. These calculations mapped behavioral combinations (*D_i_* and *S_i_*) to the outcomes (*P_i_*).”

>> line 720 (Methods)

“Although predation typically involves both pre-encounter and post-encounter processes, our model intentionally idealized behavioral effects by focusing solely on the pre-encounter phase, and thus excluded the latter. In this framework, predators gain energy as a behavioral outcome in proportion to their encounter probability, calculated as the product of the encounter probability and a constant parameter (wolf-gain-from-food = 8), whereas prey gain energy as a behavioral outcome, by eating grass (sheep-gain-from-food = 1) when they successfully evade predators (Supplementary Table 1). Accordingly, the payoff of wolves (*P_w_*) was defined as the probability of encountering prey (*P_w_* = *p_w_*), and the payoff of sheep (*P_s_*) as the probability of evading predators (*P_s_* = 1 – *p_s_*). *P_s_*, *P_w_*, and differences in payoffs (*P_diff_* = *P_s_* – *P_w_*) were calculated at each time step.”

- (ii) Related to this, the game considered describes neither a learning process (the prey cannot learn from a failure, it just dies) nor completely an evolutionary process (the predator can learn). The authors clearly realise this (during the discussion, L379), but don’t really offer the reader a way to reconcile these difficulties. This is for sure a conceptual problem that I’d like the authors to address, in that there are different timescales at play here: the predator behaviour can change really quickly but the prey behaviour changes slower.

- (iii) But that is a bit philosophical. More proximately: A Nash equilibrium can be reached only if the players have full knowledge of the game payoffs, and I cannot see how the predators and prey can estimate the encounter probability for any pair of strategies, including for that matter the one that they currently possess. And roughly stated, if players cannot estimate any of the payoff entries, we are in the realm of evolutionary game theory. I would suggest therefore that the authors do one of two things: (i) compute the evolutionarily stable strategies instead of Nash equilibria, and derive all biological inferences from there, OR (ii) give a reason for why computing the ESS is not required, including an explicit description of how the Nash equilibrium is supposed to be reached over time by the predator and prey. If the overarching question is biological, i.e. “what should one expect the predator and prey strategies to be after a long time?”, the paper would benefit from an exact statement of how the results from this model answer that question. (P.S. It is known that all Nash equilibria need not be evolutionarily stable strategies (see e.g. the book of Broom and Rychtar).)

> We thank Reviewer 1 for pointing out the asymmetry in the interaction between predators and prey—that is, predators are capable of learning, whereas prey are not. We apologize for the insufficient explanation in the original manuscript. To address this, we added two paragraphs to the Discussion section: one discusses how predators, within the framework of standard game theory, can search the payoff landscape and reach Nash equilibrium through learning; the other discusses how prey, instead of operating within standard game theory, are likely to reach evolutionarily stable strategies (ESS) through evolutionary processes, as described in evolutionary game theory. These additions aim to clarify the differences in strategy search mechanisms and the evolutionary bases of attack and defense behaviors.

>> line 510

“Considering that attack and defense behaviors are directly subject to selection through predation, we discuss possible evolutionary mechanisms underlying these behaviors[49]. In standard game theory, players are assumed to know the full strategy sets and payoffs of all others. In contrast, such assumptions rarely apply to real predator and prey interactions. However, predators have the opportunity to learn from both successful and failed attacks. Although neuronal mechanisms for learning do not directly determine specific attack strategies, their presence enables predators to empirically explore the payoff landscape, using neuronal circuits that implement sensory-motor algorithms to compute their payoffs. Therefore, we considered that predators with neuronal mechanisms for learning, rather than directly executing innate attack behaviors, are likely to be favored by natural selection[50–55]. As a result, the Nash equilibrium attack behaviors acquired through learning, functioning as short-term strategies, will be flexible on a short time scale[33,34].

In contrast, prey do not have the opportunity to learn from fatal defense failures. As a result, they cannot explore Nash equilibrium defense behaviors in standard game theory on the payoff landscape through trial and error[56]. For this reason, the evolution of defense behaviors should be discussed within the framework of evolutionary game theory. Prey performing appropriate defense behaviors likely have a greater chance of survival. In the present study, we identified Nash equilibrium defense behaviors; however, it remains uncertain whether these behaviors would necessarily evolve into long-term evolutionarily stable strategies (ESS)[40,57]. This uncertainty arises because the payoff landscape reflects only short-term immediate outcomes and does not account for survival and reproduction across generations, which are essential components of fitness. Therefore, the short-term payoff landscape is not equivalent to the long-term fitness landscape, and not all Nash equilibria are ESS[45]. Building on this reasoning, prey are expected to navigate the fitness landscape in natural populations through survival and reproduction following successful defense[58]. Consequently, prey exhibiting defense behaviors with higher fitness will be favored by natural selection. As a result, neuronal circuits responsible for innate defense behaviors evolve over long evolutionary timescales, and these defense behaviors become long-term ESS. This view is consistent with experimental findings in mice, demonstrating that defense behaviors such as escape and freezing are innate and implemented in genetically hard-wired circuits[4,6–9,11,12,14,15]. These findings suggest the possible differences in strategy search mechanisms and the evolutionary bases of attack and defense behaviors under the asymmetry in predator-prey interactions[56].”

Minor major comments:

- the amount of information in the figures is really high. I appreciate that the authors have run extensive simulations, but it was very easy to get lost trying to go through all the subpanels. Speaking for myself, I still do not feel like I have a strong intuitive grasp on the results even after spending a good amount of time on the figures. Perhaps it is better to not make any changes to the figures themselves, but I would at least suggest that some changes be made to the narration so that it’s easier to get the intended message from the figures. The authors could structure their Results section in the following way: pick an example illustrative of the results and take the readers through it while building complexity and explaining the intuition along the way. Then state the bigger message that you want readers to take away from this. After this one can describe all the results more systematically like presently done. But even here, readers will appreciate if, for every sentence of the form “$\bar{S}$*_i_* changes as *D_i_* increases” (not quoting from the MS here, just meant to be an example sentence), there is a sentence immediately after this that states the same result in a biologically straightforward way. This will not only help the results to be accessible to a wider audience, but also guide the intuition of those who may be familiar with modelling.

> We sincerely apologize for not presenting the results in a sufficiently clear manner. We deeply appreciate Reviewer 1’s patience in reading our manuscript and your helpful suggestions. Instead of describing the results mainly through symbolic expressions, we revised the Results section by first providing a verbal explanation and then presenting the key findings about the relevant figures. After that, we included descriptions using symbols and concluded each section with a summary. We applied this format consistently to the descriptions of Figures 1 through 5. We sincerely hope that these revisions have made the results easier to understand. Once again, we apologize for the earlier lack of clarity.

For example, for the Grid world results, an example is first given in words for Fig. 1,

>> line 187

“Figure 1A illustrates a representative example in which the predator chases and the prey escapes (*D_s_* = *D_w_* = 2 and *S_s_* = *S_w_* = 1). Initially (t1), neither agent detects the opponent, as they are outside each other’s detection range (indicated by the dotted lines), and they move to adjacent shaded grids, which represent their possible next positions. At the next time step (t2), both agents detect each other, increase their speed to 2, and move to new shaded grids. If both agents are located at the same grid at the final time step (a-t2), they encounter and predation occurs (t3).”

>The main points of the results were then explained in words.

>> line 218

“Our analysis demonstrated that a single different Nash equilibrium behavior emerged depending on the combination of the agents’ sensory abilities as a pure strategy. In particular, the prey’s sensory ability (*D_s_*) had a substantial effect on the Nash equilibrium behaviors of both agents.”

>Following on from this, we have used symbols to describe them. Not all of the descriptions are reproduced here, but a similar style was kept in mind.

In addition, we included in the Results section brief explanations of key aspects described in the Methods that we believe are important for improving clarity. These include, for example, the definition of payoffs, the formation of pairwise contests in multi-agent environments, and the actual measurement of Nash equilibrium payoffs.

- It is quite interesting that the Nash equilibrium is a pure strategy when *D_i_* is low, but becomes a mixed strategy as *D_i_* are both = 2. Why does this happen? It happens in both the single predator-prey case and multiple predator-prey case so it seems robust, but is it a general pattern that if the predator and prey can sense each other from quite far away, mixed strategies are the expected outcome? And why doesn’t it happen when *D_i_* are equal but lower, e.g. both =1?

> We thank Reviewer 1 for the insightful question about the generation of mixed strategies. We also think this is an important issue. We apologize that we could not include all the data. Each condition was simulated six times. Mixed strategies were not observed only when *D_i_* = 2; however, switching behavior that indicates mixed strategies was also seen under the following combinations of *D_i_*, as shown in the manuscript.

>> line 376

“The Nash equilibrium behaviors exhibited either pure or mixed strategies, depending on the combinations of detection distances (*D_s_*, *D_w_*). Mixed strategies were observed for the combinations (1, 1), (1, 2), (1, 3), (2, 2), (2, 3), (3, 1), (3, 2), and (3, 3) for all *c_b_* and *r* conditions.”

> As Reviewer 1 pointed out, the mixed strategy tends to appear when the agents detect each other from a distance. We understand the question as asking whether there is a biological explanation behind the generation of the mixed strategy. We analyzed additionally as below.

Behavioral switching enhanced behavioral diversity by reducing payoffs (Fig. 3Df). This may occur because the range of possible payoffs (strategy space) varies between conditions with and without behavioral switching. We hypothesized that a broader strategy space could permit more diverse behaviors, facilitating the emergence of behavioral switching. To test this hypothesis, we compared the strategy spaces of the two conditions. However, the difference in their ranges was not substantial (Additional Fig. 2). Therefore, it is unlikely that a broader strategy space alone accounts for the emergence of behavioral switching. The reason for the emergence of behavioral switching when the agents detect each other from a distance remains unclear and requires further investigation.

**Additional Fig. 2** The pooled *P_s_* and *P_w_* data calculated across all *S_i_* combinations under *D_i_* combinations where behavioral switching occurred were designated as “bs” (behavioral switching) [*D_s_* and *D_w_* were (1, 1), (1, 2), (1, 3), (2, 2), (2, 3), (3, 1), (3, 2), and (3, 3) for all *c_b_* and *r*], and those under all other *D_i_* combinations as “nbs” (non-behavioral switching). The full range of payoffs from all behaviors was plotted on a scatterplot with *P_s_* on the x-axis and *P_w_* on the y-axis. The data corresponding to bs was shown in dark gray plots, and those for nbs in light gray plots. Ranges of each region represent the strategy spaces of bs and nbs. The left diagram is a scatter plot with nbs on top of bs. The right diagram shows bs on top of nbs. No marked difference was observed between the two strategy spaces.

- It is clever to do the stochastic speed change strategy as a “control” of sorts. But

(i) I do not understand the authors’ claim here and elsewhere that the single element algorithm is “sufficient to achieve a Nash equilibrium”. The existence of a Nash equilibrium is always guaranteed when the set of strategies is finite, so it should not at all be surprising that there exists a Nash equilibrium for any of these games. And what does it mean to “achieve” a Nash equilibrium when the actual learning/evolutionary dynamics is not explicitly modelled?

> We agree with Reviewer 1’s comment that terms such as “achieve” are not appropriate, as a Nash equilibrium is mathematically derived rather than attained. Accordingly, we have replaced them with terms such as “establish,” “exist,” “emerge”, and so on in the revised manuscript. We appreciate the reviewer’s valuable suggestion.

The comparison that is possible here is between the Nash equilibria under sensory-motor strategies and the stochastic speed change strategies. For example it would be cool to have more information on whether the stochastic speed change strategies can ever lead to the *same* Nash equilibrium (in terms of payoffs and population sizes) as the sensory-motor algorithm.

(ii) In L312+ the results show that these Nash equilibria are in general different. I would like a bit more intuition here too, e.g. why is the effect of the single element algorithm different on the predator and prey? It decreases *N_w_* but increases *N_s_*. And also predators went extinct under sensory-motor but never prey. These asymmetries are fundamental to the results so would be nice if they are given clearer explanations.

> We thank Reviewer 1 for pointing out the difference in outcomes between the sensory-motor algorithm and the single-element motor algorithm (referred to as the non-sensory motor algorithm in the revised manuscript). We had noted this difference, but the description in the Results and the corresponding discussion were insufficient. We apologize for this. In response, we have revised the Result section followingly.

>> line 460

“Despite the similarities, we found substantial differences between the non-sensory motor and sensory-motor algorithms. Compared with the non-sensory motor algorithm, the sensory-motor algorithm decreased encounter frequency (*E*) and predator number (*N_w_*) (Fig. 5A, B), while increasing prey number (*N_s_*) and prey payoffs ($\bar{P}$*_s_*) (Fig. 5C, E). Additionally, the sensory-motor algorithm resulted in numerous instances of $\bar{P}$*_w_* = 0, indicating predator extinctions (Fig. 5D). These results indicate that the sensory-motor algorithm resulted in asymmetrical effects between agents, leading to an advantage for the prey. This suggests that the sensory-motor algorithm may represent an adaptive advantage for the prey.”

> We revised the manuscript by adding a paragraph below in the Discussion.

>> line 555

“Given that movement variability alone can generate equilibria, we further examined how the presence of sensory input influences outcomes. Compared to the non-sensory motor algorithm, the sensory-motor algorithm was more adaptive for prey. The prey’s Nash equilibrium payoffs were greater under the sensory-motor algorithm than under the non-sensory motor algorithm (Fig. 5C, E), whereas predators’ payoffs showed no such advantage (Fig. 5B, D), indicating an asymmetrical effect between the agents. This asymmetry likely stems from the IBM design: in the sensory-motor algorithm, agents, after detecting an opponent, did not account for (1) the opponent’s spatial direction (sensory directionality) or (2) their own movement direction (motor directionality). Consequently, prey could increase their payoffs by moving in any direction as long as predators were absent, whereas predators needed to move toward prey for successful predation, structurally favoring prey. Furthermore, among the behaviors generated by the sensory-motor algorithm, behavioral switching particularly benefited predators (Fig. 3D), potentially allowing them to counter prey strategies. These functional and adaptive differences raise questions regarding their phylogenetic relationships.”

- Encounter probabilities with one predator and one prey: since there are a finite number of grid cells, is it not guaranteed that the predator will eventually be on the same grid cell as the prey, irrespective of all aspects of the strategy? Why is there variation then in the encounter probability? It would help a lot here if the authors detail how this probability is calculated and not just point the reader to the script they have written (L455).

> We appreciate Reviewer 1’s question regarding the R script used to calculate the encounter probability for one-on-one interactions in the Grid world. We sincerely apologize for the lack of explanation. In this script, instead of simulating agent movements over multiple time steps in a finite grid, we calculated the encounter probability (*p*) based on a one-step movement from initial positions where agents could potentially interact, under different combinations of *S_i_* and *D_i_* (*i* = *w* or *s*). We have now clarified this point in both the Methods and Results sections.

>> line 198 (Result)

“Rather than simulating agent movements over multiple time steps in a finite grid, we calculated encounter probabilities (*p*) based on a single-step forward transition from initial positions where interactions could occur, under different combinations of *D_i_* and *S_i_* (Supplementary Program, EncounterProbabilities_gridworld.txt).”

>> line 631 (Methods)

“We examined the encounters between agents in a single time step. Because the maximum value of *D_i_* was 2, we prepared 5 ✕ 5 grids in the x−y plane with coordinates (x, y) = (0 ~ 4, 0 ~ 4). To set the relative positions of the agents, a predator was placed at coordinate (2, 2), and the prey was placed at arbitrary coordinates. When *D_i_* = 2, the agents recognized the opponents at the outer edge, and the combinations in which they were adjacent to each other were excluded. For all combinations of agent coordinates, the next positions were determined based on *D_i_* and *S_i_*. We wrote a script to calculate the encounter probability (*p*) from the number of encounters and the total number of combinations using R[65] (Supplementary program, EncounterProbabilities_gridworld.txt). In this R script, rather than simulating agent movements over multiple time steps in a finite grid, we calculated *p* based on a single-step forward transition from initial positions where agents could potentially interact, under different combinations of *S_i_* and *D_i_*.”

Minor comments:

L201 “constant sum game only when….both agents were the same”. I see how *P_s_* + *P_w_* = 1 when *E* is zero. But the other claim is that *P_s_* + *P_w_* = 1 when *N_s_* = *N_w_* and I don’t think this is always true because if we set *N_s_* = *N_w_* =some number k, then *P_s_* + *P_w_* = 1 - *E*/(*E*+k) + *E*/k = k/(*E*+k) + *E*/k which is not necessarily 1. It adds up to [k^2 + *E*(*E*+k)]/[k(*E*+k)] unless I’m making an embarrassing mistake :)

> We sincerely thank Reviewer 1 for pointing out our mistake. We apologize for the careless error. As noted, “*N_w_* = *N_s_*” was incorrect and should be “*N_w_* = *E* + *N_s_*”. We have corrected this in the revised manuscript as follows.

>> line 328

“In this framework, the game based on the multiple predators and prey interaction is constant-sum only under specific conditions: when the agents did not encounter each other (*E* = 0) or when the numbers of the agents before interaction are the same (*N_w_* = *E* + *N_s_*). Under these conditions, the sum of payoffs is exactly one (*P_s_* + *P_w_* = 1 − *E*/(*E* + *N_s_*) + *E*/*N_w_* = 1). However, in general, when encounters occur (*E* ≠ 0) and *N_w_* ≠ *E* + *N_s_*, the sum deviates from one (*P_s_* + *P_w_* ≠ 1). Moreover, when the total number of prey and encounters (*E* +*N_s_*) is greater than the number of predators (*N_w_*), the sum of payoffs becomes greater than one (*P_s_* + *P_w_* > 1). This positive-sum condition arises because *E*/*N_w_* − *E*/(*E* + *N_s_*) > 0.”

-When individuals change their speed post detecting something, which direction do they go in? The answer to this is likely that they do not change direction, but then I would ask how the direction of movement decided at all, both before and after something is detected within the sensory radius. This is, as far as I can tell, not described in the methods section.

> We sincerely apologize for the lack of explanation. The agent’s initial movement direction is randomly determined at the beginning of the simulation. At each subsequent time step, the agent randomly changes its direction within a 90-degree forward range, regardless of whether it detects the opponent or not. We have added this description to the Methods section.

>> line 657

“The agent’s movement direction changes at each time step, randomly within a forward-facing 90-degree range (Fig. 2B, and Supplementary_movie.mov).”

- It would also become much easier to get a grasp of the model if the authors provide movies visualising the simulations.

> Thank you for pointing this out. We apologize for the oversight—we have added the NetLogo model video to the Supplementary Information.

>> line 242

“Using a programming language and an integrated development environment called NetLogo[43], we created a simulator to empirically solve the game (Fig. 2A, B; Supplementary_movie.mov; Supplementary Program, EncounterProbabilities_sensory_motor.nlogo).”

L34 “Recent studies…defense behaviours.” This sentence requires references to the recent studies.

> We apologize for the unclear sentence. The phrase “Recent studies…” referred to the previous sentence: “The fundamental mechanisms of defense behaviors in mice are innate and implemented by genetically hard-wired neuronal circuits [3,17–20].” The correct references were [3,17–20]. Because our wording was not appropriate, we revised the sentence as below to clearly link “Recent studies” with the corresponding references.

>> line 119

“Recent studies have demonstrated that the fundamental mechanisms of defense behaviors in mice are innate and executed by genetically hard-wired neuronal circuits[3,18–21], elucidating the level of implementation of these defense behaviors.”

- L37 “numerically evaluating the modifications...” — modifications in what? This is extremely cryptic and actually took me quite a while to figure out, and my best guess now is that the authors mean numerically evaluating the modifications in encounter probabilities. This sentence should for sure be completed, and even then the notion of encounter probabilities has not been introduced yet at that point.

> We sincerely thank Reviewers for their comments. We have added an explanation of encounter probability to the first paragraph and clarified this sentence by adding the phrase ‘the modifications in encounter probabilities’.

>>line 110

“It has been argued that predators’ and prey’s behaviors influence the spatial arrangement between them, resulting in encounter probability and directly impacting predation[16].”

>> line 124

“Therefore, numerically evaluating the modifications in the encounter probabilities caused by the agents’ behaviors aids in understanding the algorithms and computations of the behaviors.”

- L67 “the single-element motor and sensory-motor…respectively”. This sentence is really quite vague — I am not sure what the difference is between a “minimal” component and a “fundamental” component, and how do I distinguish between a function of attack/defense and something that is only comparable to attack/defense?

> We apologize for the unclear explanation of the common and distinct features of the single-element motor algorithm (referred to as the non-sensory motor algorithm in the revised manuscript) and the sensory-motor algorithm. We revised the sentences as below to clarify that both algorithms lead to Nash equilibria, although they result in different outcomes.

>> line 163

“Moreover, a simple algorithm without sensory input (a non-sensory motor algorithm) leads to Nash equilibria. Furthermore, the non-sensory motor and sensory-motor algorithms differed in adaptive significance, resulting in distinct outcomes for the agents.”

- I don’t think the figures are all vector images (e.g. pdf) but might be wrong on this. Since the figures demand a lot of zooming, it would be good if they did not lose resolution when zoomed into.

> Thank you very much for pointing out the illustrations. We apologize for this and have attached the illustrations with sufficient resolution to the revised manuscript.

##########

Reviewer 2

- The manuscript investigates predator-prey dynamics in an agent-based model through a game-theoretic approach. Hereby it focuses on an idealized behavioral model. It starts by analyzing the interaction of a single prey and a single predator in a lattice environment and identifying Nash equilibria depending on sensory and motor capabilities of both predator on prey agents. Then it moves on to a multi-agent scenario, which is analyzed in detail. Finally, in addition to the full sensory-motor algorithm controlling the behavior, the authors also explored the dynamics of the system with agents using a simple motor algorithm independent of any sensory input.

The topic of the manuscript is certainly interesting, and of potential interest to theoretical/computational biologists. I also do acknowledge that there is a gap of systematic investigation of spatially explicit agent-based models for predator-prey dynamics and how physical and sensory constraints may affect evolutionary dynamics and optimal behavioral strategies. Some of the results are quite nice, like the identification of different prey and predator strategies depending on the sensory capabilities of both agent types, or investigation of equilibrium strategies for a pure motor algorithm, independent on the sensory input.

However the present manuscript has a number of important issues, and highly problematic parts. This issues need to be thoroughly addressed, before the paper can be at all considered for publication withPLoS Comp. Biol. In its current form it is not acceptable. Below, I address the different points in detail. In addition, I provide an annotated PDF with some additional comments (partly overlapping).

> We sincerely thank Reviewer 2 for identifying these important issues, and we deeply apologize for the numerous shortcomings in our original submission. We are truly grateful for the reviewer’s patience in explaining these points clearly, which helped us to better understand and improve the manuscript. We sincerely appreciate the reviewer’s numerous suggestions for improving the English throughout the manuscript.

Major points:

- 1) The authors follow a game-theoretic approach, however the multi-agent scenario appears to resemble more models studied in spatially-explicit evolutionary games. Here, the authors seem to completely ignore existing and relevant literature on evolutionary games and evolutionary stable strategies:

Mitchell, W. A. (2009). Multi‐behavioral strategies in a predator–prey

game: an evolutionary algorithm analysis. Oikos, 118(7), 1073-1083.

or a review-type perspective article:

Lima, S. L. (2002). Putting predators back into behavioral predator–prey

interactions. Trends in Ecology & Evolution, 17(2), 70-75.

> We sincerely apologize for our lack of expertise and for the insufficient citation of relevant literature. We sincerely thank Reviewer 2 for his appropriate remarks. In the intro, we cited a previous study (Mitchell 2009), emphasizing different aspects of behaviors in spatially explicit evolutionary games.

>> line 151

“An IBM has been previously used to study how the behaviors of predators (learning, attack timing, and movement) and prey (random movement, vigilance, and preferred area) affect each other’s habitat spatial distribution, thereby altering encounter probabilities[35].”

> The review article by Lima (2002) was cited in the Introduction section of the original manuscript, and we have now added a clarifying statement to emphasize its relevance.

>> line 126

“As proposed by Lima[22], this requires modeling the strategic behaviors of both predator and prey, allowing the mutual influence of attack and defense behaviors on their payoffs, based on the encounter probabilities, to be analyzed using game theory[22–28].”

- 2) Linked to the first point: The authors make claims about evolutionary selectionof the Nash equilibrium (NE) strategies. However, when talking about evolutionary selection, the more appropriate concepts are those of biological fitness and of evolutionary stable strategy (ESS). Here it has to be highlighted that every ESS is a NE, but not every NE is an ESS. This needs to be clearly distinguished, and corresponding statements on the relevance to natural evolution should be reviewed and potentially toned down.

> We sincerely apologize for the inadequacy of our original discussion on the evolution of behavior. We are deeply grateful that the reviewer not only pointed out this core weakness clearly and concisely, but also guided us in the right direction. Following the reviewer’s suggestions, we differentiated the payoff landscape and the fitness landscape, and highlighted the asymmetry between predators and prey—namely, that predators can learn from failed attacks, whereas prey have no such opportunity. This perspective allowed us to better discuss potential differences in the evolutionary mechanisms of attack and defense behaviors. We revised the manuscript accordingly and added two paragraphs to the Discussion section, as shown below.

>> line 510

“Considering that attack and defense behaviors are directly subject to selection through predation, we discuss possible evolutionary mechanisms underlying these behaviors[49]. In standard game theory, players are assumed to know the full strategy sets and payoffs of all others. In contrast, such assumptions rarely apply to real predator and prey interactions. However, predators have the opportunity to learn from both successful and failed attacks. Although neuronal mechanisms for learning do not directly determine specific attack strategies, their presence enables predators to empirically explore the payoff landscape, using neuronal circuits that implement sensory-motor algorithms to compute their payoffs. Therefore, we considered that predators with neuronal mechanisms for learning, rather than directly executing innate attack behaviors, are likely to be favored by natural selection[50–55]. As a result, the Nash equilibrium attack behaviors acquired through learning, functioning as short-term strategies, will be flexible on a short time scale[33,34].

In contrast, prey do not have the opportunity to learn from fatal defense failures. As a result, they cannot explore Nash equilibrium defense behaviors in standard game theory on the payoff landscape through trial and error[56]. For this reason, the evolution of defense behaviors should be discussed within the framework of evolutionary game theory. Prey performing appropriate defense behaviors likely have a greater chance of survival. In the present study, we identified Nash equilibrium defense behaviors; however, it remains uncertain whether these behaviors would necessarily evolve into long-term evolutionarily stable strategies (ESS)[40,57]. This uncertainty arises because the payoff landscape reflects only short-term immediate outcomes and does not account for survival and reproduction across generations, which are essential components of fitness. Therefore, the short-term payoff landscape is not equivalent to the long-term fitness landscape, and not all Nash equilibria are ESS[45]. Building on this reasoning, prey are expected to navigate the fitness landscape in natural populations through survival and reproduction following successful defense[58]. Consequently, prey exhibiting defense behaviors with higher fitness will be favored by natural selection. As a result, neuronal circuits responsible for innate defense behaviors evolve over long evolutionary timescales, and these defense behaviors become long-term ESS. This view is consistent with experimental findings in mice, demonstrating that defense behaviors such as escape and freezing are innate and implemented in genetically hard-wired circuits[4,6–9,11,12,14,15]. These findings suggest the possible differences in strategy search mechanisms and the evolutionary bases of attack and defense behaviors under the asymmetry in predator-prey interactions[56].”

- 3) There is another conceptual issue: The multi-agent scenario, does not really align with a pure game-theoretic approach. Given that the agents reproduce and interact through predation, the system approaches stationary numbers (densities) of prey *N_s_* and predators *N_w_*, respectively. So *N_s_*, *N_w_* are emergent variables the entire system converges to, analogous to fixed-points in Lottka-Volterra dynamical models, and are not related to individual-level strategy choice, which is what game theory and Nash equilibrium (NE) are concerned with. Thusdiscussing *N_s_*, *N_w_* as part of NE appears ill defined, and may potentially lead to biologically problematic conclusions (see bvelow).

- (comment in pdf)

“What I find problematic here, is that in this multi-agent game that *N_w_* and *N_s_* are emergent properties, not only *E* (as in the two-player game), and thus they affect the average payoffs. In a game theoretic framework, typically number of agents is a constant parameter. It is highly problematic if we interpret Nash equilibria as strategies: predator and prey do not control individually their numbers - it cannot be an aspect of a strategy of individuals. It is an emergent property, a macro-level dynamic equilbrium similar to fixed points in Lotka-Volterra models.”

> We thank you for pointing out the potential issue with applying game theory to a multi-agent system. We apologize for not providing a sufficient explanation of our experimental design. Although our IBM includes many agents (population size *N_i_*, for *i* = *w* or *s*), we treated the encounter frequency *E* and population size *N_i_* as intermediate values. As the reviewer pointed out, agents cannot regulate their population size *N_i_*. However, both *E* and *N_i_* are under the influence of agents' behaviors. Therefore, we defined agent payoffs (*P_i_*) based on the encounter probabilities derived from *E* an *N_i_*. We consider that *P_i_* can be regarded as the outcomes obtained by each agent. This explanation has been added to the Results sections, as shown below.

>> line 259 (Results)

“Combinations of *D_i_* and *S_i_* determined the agents’ behaviors, and these behavioral combinations influenced the encounter frequencies between agents (*E*), which in turn reflected the number of agents (*N_i_*). Both *E* and *N_i_* are under the influence of the behaviors. Using *E* and *N_i_* as intermediate values, we calculated agents’ encounter probabilities at each time step.”

> In game theory, games are typically defined between two players. However, in biological systems, interactions often occur among more than two individuals. Solving such multi-player games, where all individuals in a population are treated as players, is complicated and computationally difficult. To make the analysis more tractable, it is common to simplify the model by assuming that all individuals belong to a potential pool of players and that the game takes place as a series of pairwise contests between two players (Broom, Mark; Rychtář, Jan. Game-Theoretical Models in Biology, Chapman & Hall/CRC, 2022, p.15).

We adopted this approach in our experiments. We then assumed that pairwise contests occur within the multi-agent environment, allowing us to analyze the system as a two-player game. This explanation has been added to the Results, Discussion, and Methods sections, as shown below.

>> line 284 (Results)

“Although the IBM included many agents, we treated the population as a pool of potential players engaging in pairwise contests in a two-player game, rather than assuming that every individual is a player[45].”

>> line 479 (Discussion)

“We treated the agent population as a pool of potential players engaging in pairwise contests in a two-player game, rather than assuming that every individual is a player[45], and estimated individual-level payoffs.”

>> line 703 (Methods)

“An individual agent’s behavior is determined by a combination of detection distance *D_i_* and speed change *S_i_*. The agents’ behaviors influence both the encounter frequency (*E*) and the number of agents (*N_i_*) in the model. These values dynamically change over time as emergent properties. We considered behaviors as fixed parameters, treated *E* and *N_i_* as intermediate values, and calculated agents’ payoffs (*P_i_*) as outcomes using *E* and *N_i_* at each time step.”

>> line 748 (Methods)

“The NetLogo IBM model includes many agents; however, it is formally interpreted as a two-player game because the individual behaviors of agents seek payoffs through pairwise encounters. The resulting bimatrix of *P_i_*, derived from these behaviors, serves as the payoff matrix of the game, wherein each agent selects one of the eleven motor strategies (*S_i_*) based on its specific sensory ability (*D_i_*). This framework allows for a systematic analysis of the strategic interactions between the predator and prey within the model[68,69].”

> We apologize for the errors in the previous version of the manuscript. The formulas for encounter probabilities and payoff definitions were incorrectly written. We have corrected them as follows: *P_w_* = *E*/*N_w_*, *P_s_* = *E*/(*E* + *N_s_*). However, the data were calculated using the correct formulas, so no figures or results were changed.

>> line 263 (Results)

“In the NetLogo program, *E* and *N_i_* were recorded after agent interactions. The number of predators (*N_w_*) included both encountered and unencountered predators, whereas the number of prey (*N_s_*) excluded prey that were removed due to predation. Thus, predator and prey encounter probabilities (*p_w_* and *p_s_*) were calculated as *E*/*N_w_* and *E*/(*E* + *N_s_*), respectively.”

>> line 713 (Methods)

“As *E*, *N_w_*, and *N_s_* were reported at the end of the “to go” procedure after the agents interacted in the NetLogo program, *N_w_* included the number of wolves both with an encounter and no encounter; on the other hand, *N_s_* did not include the number of sheep died of predation with an encounter but included only with no encounter. Therefore, encounter probabilities of wolves (*p_w_*) and sheep (*p_s_*) were defined as *E*/*N_w_* and *E*/(*E* + *N_s_*), respectively. The formula appeared asymmetrical, but the encounter itself is symmetrical, with contributions from a sheep and a wolf.”

- 4) One core interpretation of the results does make biologically no sense. In the discussion section (line 334) it reads: “...the prey have an incentive to receive an attack...”. However, individual prey have never an incentive to receive attacks. What is observed here are likely “spourious” relative benefits as a consequence of *N_s_* and *N_w_* being emergent variables (not fixed parameters). Furthermore, this may be also linked to the NE not being an ESS. Thus the corresponding discussion parts need a thorough revision.

- (comment in pdf)

“I think this is misleading, as it suggest that certain attack “strategy” of the predator increases a payoff of prey. However, prey never benefit from predators attacking them. Optimal scenario for prey maximizing payoffs: no attacks / no predators. It is rather about increase of relative payoffs in the presence of predators when predators follow the NE-strategy.”

> As pointed out by Reviewer 2, we agree that the statement “the prey have an incentive to receive an attack” was inappropriate. We have removed this sentence and apologize for the confusion. To clarify the results in the context of a two-player game based on pairwise contests under a multi-agent environment, we added an explanation based on the observed patterns of encounter frequency (*E*) and numbers of agents (*N_i_*). Under relatively constant *E*, a decrease in *N_w_* leads to an increase in *N_s_*, which results in a higher prey payoff [*P_s_* = 1 - *E* / (*E* + *N_s_*)]. We interpret this as a possible consequence of intensified competition among predators. This explanation has been added to the Results and Discussion sections, as shown below.

>> line 365 (Results)

“These results suggest that when predators detect prey from a greater distance and attack according to the Nash equilibrium under conditions without prey defense, both predators’ and prey’s payoffs can increase. This outcome is due to a decrease in the number of surviving predators and an increase in prey numbers. These results suggest that prey may indirectly gain higher payoffs in the presence of predators, due to increased competition among the predators.”

>> line 487 (Discussion)

“When predators detected prey from a greater distance and attacked according to the Nash equilibrium, both predator and prey payoffs increased (Fig. 3Cd). This outcome occurred under conditions without prey defense and was attributed to increased competition among predators (Fig. 3B, C; Supplementary Figs. 6, 7). These results suggest that prey may obtain higher payoffs even in the presence of predators.”

> The sentences “In behavioral combinations where both agents’ payoffs increase together, predators have an incentive to attack” and “When the prey defended against a predator’s attack, the prey’s payoff increased; thus, the prey has an incentive to defend itself.” are kept in the revised manuscript.

>> line 492

“In behavioral combinations where both agents’ payoffs increase together, predators have an incentive to attack. When the prey defended against a predator’s attack, the prey’s payoff increased; thus, the prey has an incentive to defend itself. These findings suggest that each of the agents initiates attack and defense behaviors.”

- 5) The multiplayer game is insufficiently explained. For example, it is stated in the methods section that the model is in continuous space. However, in the screenshots it is apparent that at least the “grass” resource appears discretion, also an encounter (i.e. predation event)happens if prey and predator are in the same patch. Here, I assume “patch” refers to the lattice site.

Furthermore, it is not clear what type of movement the agents perform in the 2D multi-agent simulation. If this is continuous in space and time, then it could be either some sort of random walk or some sort of active Brownian motion.

- (comment in pdf)

“How is *S_i_* now continuous, and can assume values fractional values (e.g. 0.5)? One possibility this is due to a stochastic movement algorithm in NetLogo. However, later in Methods its claimed its a continous space simulation. But in the screenshot ressources are apparently defined on the grid, and its also stated that captures happen if predator and prey are on the same “patch” (lattice site?). How does this work together? It seems to be then some hybrid continuous-discrete model. Defintiely needs clarification.”

> We apologize for the insufficient explanation of the multi-agent IBM setup. Below, we describe the agents’ speed change parameter (*S_i_*) and the environmental setting for agent interactions (Patch).

The value of *S_i_* was set to one of eleven discrete values from –1 to 1 in 0.2 increments. Although the values changed gradually, they were not continuous (for example, 0.5 was not included). For each combination of detection distances (*D_i_*), there were 121 (= 11 × 11) combinations of *S_i_* values between predators and prey. For each condition, we ran simulations, recorded *E* and *N_i_*, and calculated *P_i_*.

Regarding the environment, agents could occupy any real-valued (x, y) coordinates and move smoothly across the space. Patches represented a discrete spatial structure used to control grass regrowth, but they did not constrain agents’ movement. The agents’ movement direction changed randomly within 90 degrees ahead at each time step. These specifications are the same as those in the original NetLogo model (Wolf Sheep Predation, https://ccl.northwestern.edu/netlogo/models/WolfSheepPredation). To supplement Fig. 2B, we included a video of the IBM in the supplementary materials. This explanation has been shown to the Methods section, as shown below.

>> line 651 (Methods)

“The model was composed of agents (predators and prey) and patches that represent the environment. The agents moved and interacted in a continuous two-dimensional space. They could occupy any real-valued (x, y) coordinates, ensuring smooth movement. The patches served as a discrete spatial structure for managing environmental states, such as grass regrowth, but did not constrain the agents’ movement. The agents were not restricted to moving from patch to patch. The agent’s movement direction changes at each time step, randomly within a forward-facing 90-degree range (Fig. 2B, and Supplementary_movie.mov). These specifications are consistent with those used in the original Wolf-Sheep Predation model provided in the NetLogo model library.”

>> line 693 (Methods)

“The motor ability was equal to speed change *S_i_*, which considered 11 different values ranging from −1 to 1 at intervals of 0.2. The combinations of *D_i_* and *S_i_* defined 44 different behaviors as their strategies of the agents. The interactions between these behavioral strategies resulted in 44^2^ (= 1,936) unique strategy combinations.”

>> line 733 (Methods)

“For each combination of *D_i_*, 121 simulations were run, in which each engaged in 11 different behaviors (11 *S_w_* and 11 *S_s_*).”

Minor points:

- The language needs some revision. There are some suggestions/examples highlighted and annotated in the attached PDF.

>We sincerely apologize for the shortcomings in our original manuscript. We deeply appreciate Reviewer 2’s patience and detailed comments on the PDF. As suggested, we highlighted all revisions in the revised manuscript. We replaced all instances of “single-element motor algorithm” with “non-sensory motor algorithm.” We also replaced expressions like “generate” or “achieve” a Nash equilibrium with more appropriate terms such as “exist” or “establish”.

- The article is also rather hard to read as the results are quite dense with a lot of variables, which make it hard to recall them. I suggest adding some redundancy by spelling out the names of different parameters at multiple locations, e.g. writing “encounter frequency *E*” instead of only writing “*E*” or similar for other variables.

> We apologize for the lack of clarity in our manuscript. We sincerely appreciate Reviewer 2’s patience and constructive suggestions. As recommended, we added the full terms before each abbreviation (e.g., “encounter frequency *E*”) when they first appear in each section.

Others or comments on PDF

- “Sounds very much like an evolutionary predator prey game. However any references to relevant literature are missing.”

- “It appears the authors completely ignore any literature on evolutionary games / artificial life simulations.”

> We apologize for the lack of appropriate references to the literature on evolutionary game theory. We added the articles below and cited them in the revised manuscript.

Smith, J. Maynard. “The theory of games and the evolution of animal conflicts.” Journal of theoretical biology 47.1 (1974): 209-221.

Maynard Smith J. Game theory and the evolution of behaviour. Proceedings of the Royal Society of London - Biological Sciences. 1979;205: 475–488. doi:10.1098/rspb.1979.0080

DeAngelis DL, Mooij WM. Individual-based modeling of ecological and evolutionary processes. Annu Rev Ecol Evol Syst. 2005;36: 147–168. doi:10.1146/annurev.ecolsys.36.102003.152644

Adami C, Schossau J, Hintze A. Evolutionary game theory using agent-based methods. Phys Life Rev. 2016;19: 1–26. doi:10.1016/j.plrev.2016.08.015

- “Where does this expression for *P_s_* come from?”

> We thank Reviewer 2 for this insightful question regarding the definition of agent payoffs based on encounter probabilities. We apologize for the lack of detailed explanation. Below, we describe how the payoffs are defined in the IBM implemented in NetLogo. In the NetLogo program, the number of individuals and encounters are recorded at the end of the procedure, so the recorded PREY population numbers do not include predated individuals. Therefore, *p_s_* = *E*/ *N_s_* is not accurate and should be divided by the number of individuals before predation occurs, *p_s_* = *E*/(*E* + *N_s_*). On the other hand, the predator population includes predators that have preyed. Therefore *p_w_* = *E*/*N_w_*. This explanation has been added to both the Results and Methods sections.

>>line 263 (Results)

“In the NetLogo program, *E* and *N_i_* were recorded after agent interactions. The number of predators (*N_w_*) included both encountered and unencountered predators, whereas the number of prey (*N_s_*) excluded prey that were removed due to predation. Thus, predator and prey encounter probabilities (*p_w_* and *p_s_*) were calculated as *E*/*N_w_* and *E*/(*E* + *N_s_*), respectively. In general, predation consists of both pre- and post-encounter processes[35], but in the IBM, these processes were simplified by treating the encounter itself as predation. In this framework, predators gain energy in proportion to their encounter probability, and prey gain energy by eating grass after evading the encounter in the IBM (Supplementary Table 1). Accordingly, we defined agents’ payoffs (*P_i_*) based on their encounter probabilities: predator payoffs (*P_w_*) = *p_w_*, and prey payoffs (*P_s_*) = 1 − *p_s_*.”

>> line 713 (Methods)

“As *E*, *N_w_*, and *N_s_* were reported at the end of the “to go” procedure after the agents interacted in the NetLogo program, *N_w_* included the number of wolves both with an encounter and no encounter; on the other hand, *N_s_* did not include the number of sheep died of predation with an encounter but included only with no encounter. Therefore, encounter probabilities of wolves (*p_w_*) and sheep (*p_s_*) were defined as *E*/*N_w_* and *E*/(*E* + *N_s_*), respectively. The formula appeared asymmetrical, but the encounter itself is symmetrical, with contributions from a sheep and a wolf.”
