# Supplementary material for: Nash equilibrium of attack and defense behaviors between predators and prey
Source: PLoS Comput Biol. 2025 Nov 21;21(11):e1013730. doi: 10.1371/journal.pcbi.1013730 (PMC12671891; doi:10.1371/journal.pcbi.1013730)
Supplement: Attachment — Submitted filename: PCOMPBIOL-D-24-01911_review2.docx [file pcbi.1013730.s043.docx]

**Review 2 of “Nash equilibrium of attack and defense behaviors between predators and prey”**

First, thank you to the authors for their responses to my comments. I think the paper is clearer in many places, and I understand the work much better. It is also easier to read. However, I still think some conceptual clarifications are necessary. On reading a second time, I also have suggested some minor improvements to the text and figure captions.

I think the authors have still not provided a complete argument on some fronts:

(i) The authors do not provide a resolution for the point that not all Nash equilibria (NE) are evolutionarily stable strategies. The paragraphs added to the discussion are welcome in that they add context to the results, but they do not resolve the underlying issue: To reach a Nash equilibrium either via learning or evolution, the predator and prey require knowledge of all payoffs (encounter probabilities) which they cannot have. And to make statements about evolutionary outcomes, we still do not know if the NE described are indeed evolutionarily stable.
    The authors say this on lines 528-530 but, in my opinion, it is not clear enough to the reader that this is quite a major impediment to the biological utility of this study. Ideally, I’d like the authors to find the evolutionarily stable strategies for the considered games. I am not saying that the nash equilibrium is completely uninformative, but without knowledge of what the ESS is, it is hard to say if these results truly represent what happens over the course of evolution.
(ii) The authors, in response to a previous comment of mine, have added a sentence “Consequently… structurally favoring prey” on line 564-566. It is a (really) good observation, but I’m not sure it explains everything. I get now that the asymmetry arises from the fact that it is easier for the prey to not-die than for the predator to eat. But this effect also exists under the sensory-motor algorithm, doesn’t it? So there is still some reason that the non-sensory-motor algorithm exacerbates this already present asymmetry.
(iii) I would say that the other main biological outcome of this work is that (i) the Nash equilibrium can be pure or mixed, and increased D_i seems to systematically promote mixed strategies. Given that it is mentioned in both the abstract and author summary, the authors also seem to see this as a main result. Yet, the authors do not offer an explanation for why this happens. I appreciate that the authors made Additional Fig. 2 to alleviate my concern, but the only main text sentence that tries to offer insight into this is in Line 505-508, but this is also quite vague – what are these “multiple agent effects”, and how exactly are they attributable to “alterations in encounter frequencies and agent numbers”?
(iv) The payoffs (P_w, P_s) calculated from the NetLogo simulations are a function of what happens when the whole prey population is interacting with the whole predator population, and not what happens in an interaction between two agents on their own. The latter is what one usually puts into a payoff matrix. The authors say (line 284) that they treat the population as a pool of potential players engaging in pairwise contests. This is good, and I think it is defensible to define payoff here as “equilibrium probability of finding a prey per timestep”, but it is certainly out of the ordinary. So I think this should be said to give readers the correct context, and also emphasize the novelty of this approach.

To reiterate, I do think the approach of empirical game theoretic analysis can be useful, and is a novel way (at least in theoretical ecology and evolution) of dealing with complex scenarios. My objections only come from the fact that the biological utility of these results depends strongly on how one chooses to analyse the empirical game.

**The abstract**. Some really minor typos in the abstract:

- Line 58. change to “is a central question in …”
- L71 remove the first “the”, sentence should read “…were non-constant sum and…”
- L75 “a simple non-sensory motor algorithm was sufficient to lead to their Nash equilibria.” this is misleading because a Nash equilibrium always exists (also see below). I would suggest rephrasing to something like “Nash equilibrium behaviors were also computed under a simpler non-sensory motor algorithm.”

**Other minor comments**

**Line 722 “predators gain energy as a behavioral outcome in proportion to their encounter probability”**: this choice in the model is confusing. why aren’t predators just given energy immediately when they meet and eat a prey?

**Line 162 “Moreover,…equilibria”:** This kind of sentence is misleading as I have already pointed out. In any game with a finite number of strategies, (mixed-strategy) Nash equilibria always exist, so it is not surprising – if a payoff matrix is constructed, a (mixed) Nash eq. can be found. The authors also present such statements on e.g. Lines 158, 409, 545. I would suggest changing these and any others I might not have found. On L409 specifically: “To investigate whether sensory input is necessary for agents to establish Nash equilibria,…” is especially misleading. Sensory input is unnecessary because an NE exists of course. But it is not unnecessary because the factor being considered is sensory input, it is unnecessary because an NE always exists.

**Single step encounter probabilities**: the outcome depends strongly on the starting position of the sheep (which determines whether or not it detects the wolf at 2,2), and in which direction they both turn after the first step (whether they detected something or not). Was the encounter probability averaged over different possible pairs of initial positions and turning possibilities?

**Figures.** there are no values on any colourbars, e.g. in Figure 1B, 2C,D,E what are the minimum and maximum encounter probabilities & payoffs?

**Line 110:** typo, did you mean to say “differences in interaction probability”?
