# Supplementary material for: Nash equilibrium of attack and defense behaviors between predators and prey
Source: PLoS Comput Biol. 2025 Nov 21;21(11):e1013730. doi: 10.1371/journal.pcbi.1013730 (PMC12671891; doi:10.1371/journal.pcbi.1013730)
Supplement: Attachment — Submitted filename: R2_Response to Reviewers.docx [file pcbi.1013730.s045.docx]

2025-09-16

Dear Editor and Reviewers,

We sincerely thank the reviewers for their thoughtful and constructive feedback on the revised manuscript (R1), “Nash equilibrium of attack and defense behaviors between predators and prey.” We are grateful for the time and care they devoted to our work. The reviewers’ comments substantially improved the clarity, depth, and overall quality of the manuscript.

In this “Response to Reviewers”, we have addressed each point raised, made clarifications where needed, incorporated the reviewers’ suggestions, and enhanced the writing throughout. A detailed, point-by-point response is provided in responses to reviewers below. Reviewer comments are shown in blue, with our responses following in black, and revised sentences in red.

In the revised manuscript (R2), newly added or changed texts are written in red. In addition, we reordered the paragraphs in the Discussion section to improve clarity; the change is highlighted in yellow.

We sincerely appreciate the opportunity to revise and resubmit our work, and we thank the editors and reviewers for their continued, thoughtful guidance in this second round.

Hiroyuki Ichijo

##########

Reviewer 1

Major comments:

(1)First, the authors did not fully address my concerns—nor those raised by the other reviewer— regarding the game-theoretic treatment of the multi-player game and the potential confusion between classical game theory, evolutionary game theory, and the relationship between Nash equilibria and Evolutionarily Stable Strategies (ESS). The additional explanations partly respond to our comments, but I believe further clarification specific to this model would be beneficial.

For example, the authors treat E as an intermediate variable that is influenced by predator and prey behaviors. While this is true, E is still a population-level variable, and it would help to explain more clearly under what conditions or assumptions it is reasonable to treat E as uniform across all agents. In other words, what assumptions underlie this approach? It appears the model implicitly assumes a homogeneous population of agents interacting in a well-mixed scenario. I would suggest the authors make this assumption explicit.

Response >> We appreciate the reviewer’s point. In our framework, *E* is defined at the population level; we treat it as uniform across agents under the following assumptions: (i) a homogeneous population in which agents share identical parameters (e.g., *D_i_*, *S_i_*), (ii) well-mixed interactions with random pairing and no spatial clustering, and (iii) a time-scale separation over the analysis window such that individual fluctuations average out. We have made these assumptions explicit and revised both the Results and Methods accordingly.

(In the Results, Lines 276–284 in the revised manuscript)

“Combinations of *D_i_* and *S_i_* determined the agents’ behaviors. By design, predator and prey agents were homogeneous and shared identical sensory and motor traits. These behavioral combinations influenced the population-level number of predator–prey encounter events per time step (encounter frequency, *E*), which in turn affected the numbers of agents (*N_i_*). We assumed interactions occurred in a homogeneous, well-mixed population. Under this assumption, we approximated per-agent encounter number by the population-level *E* as an intermediate variable and applied *E* uniformly when evaluating payoffs. Using *E* and *N_i_*, we then computed the encounter probability at each time step.”

(In the Results, Lines 293–298 in the revised manuscript)

“Under the assumption of uniform interactions in a well-mixed environment, we approximated per-agent payoffs by the population-level payoffs based on the encounter probabilities. Accordingly, we defined agents’ payoffs (*P_i_*) as follows: predator payoffs (*P_w_* = *p_w_*), and prey payoffs (*P_s_* = 1 − *p_s_*). Because *P_i_* fluctuated over time, we averaged *P_i_* to obtain statistically stationary values after the transient in the simulation had settled.”

(In the Methods, Lines 795–801 in the revised manuscript)

“An individual agent’s behavior was defined by its detection distance (*D_i_*) and speed change (*S_i_*). For each parameter combination, predator and prey agents shared identical sensory and motor traits (homogeneous population) and were assumed to interact in a well-mixed environment (random pairing; no spatial structure). Under these assumptions, we treated the number of predator–prey encounter events (encounter frequency, *E*) and agents’ number (*N_i_*) at each time step in the population-level as intermediate variables. Both *E* and *N_i_* varied over time as emergent properties.”

(2)Additionally, I remain unconvinced by the current discussion contrasting short-term outcomes (which the authors associate with Nash equilibria) and the “long-term fitness landscape.” The current explanation still seems incomplete.

For a Nash equilibrium, no player can improve their payoff by unilaterally changing their strategy, assuming all other strategies are fixed. In contrast, for an ESS, one must show that a strategy adopted by the population cannot be invaded by any mutant strategy. While reproduction plays a key role, what is ultimately crucial is the relative fitness of two competing strategy phenotypes, assuming fitness-proportionate reproduction. If convincing arguments could be made about the relative fitness of prey strategies, one could potentially argue that the Nash equilibria also correspond to ESS for the prey—or not.

A particularly useful direction would be to consider the adaptive dynamics limit of rare mutants in a stationary population of prey agents playing the Nash equilibrium strategy. In this scenario, the encounter rate for the rare mutant (e.g., a single mutant in a resident NE population) should depend only on the number of predators and both the predator (Nash) strategies and the prey mutant strategy. If it can be shown that the payoffs for mutant strategies are always lower than those of the resident strategy, one could conclude that the NE corresponds to an ESS. If not, this would suggest the ESS differs from the NE.

Response >> We agree that the current Nash equilibrium (NE) versus fitness discussion needed clarification. We now explicitly separate short-term NE from long-term evolutionary stability and outline an Adaptive Dynamics test by introducing a rare mutant into a homogeneous, well-mixed resident population playing the NE strategy, computing the mutant’s invasion fitness, and assessing whether no nearby mutant can increase in frequency when rare. We have revised the Discussion accordingly to make these assumptions and criteria explicit, and to note that an NE may or may not be an ESS depending on this test.

(In the Discussion, Lines 607–628 in the revised manuscript)

“In contrast, individual prey cannot reliably learn from fatal defensive failures; hence, defense is best analyzed within an evolutionary framework. The evolutionary status of these behaviors depends on whether the resident strategy resists invasion by rare mutants. In evolutionary game theory, an evolutionarily stable strategy (ESS) is a resident strategy that cannot be invaded by any sufficiently similar rare mutant under fitness-proportionate reproduction[40,60]. Because our Nash equilibrium results were derived from single interactions, this analysis did not test rare-mutant invasion; therefore, a Nash equilibrium may or may not coincide with an ESS. A formal assessment would require an Adaptive Dynamics analysis[61] by assuming a homogeneous, well-mixed, statistically stationary resident prey population playing the Nash equilibrium strategy, introducing a rare mutant, and evaluating whether it increases or declines when introduced at very low frequency. If every sufficiently similar rare mutant fails to increase, the Nash equilibrium is an ESS; otherwise, it is not.

Although it remains unknown whether the Nash equilibrium defense strategies identified here are evolutionarily stable, the corresponding behaviors—such as escaping and freezing—are often innate or developmentally specified. In mice, for example, escaping and freezing can be elicited by genetically hard-wired circuits with minimal learning[4,6–9,11,12,14,15]. The asymmetry—predators can adjust their attack behavior through experience, whereas prey cannot learn from fatal defense failures—suggests that the mechanisms differ: predators rely on strategy learning, whereas prey defenses are largely innate and genetically specified, implying distinct evolutionary bases for attack and defense[62].”

(3)My suspicion is that, if the sensory range is fixed, then the "motor" Nash strategies likely correspond to ESS. However, if detection range becomes an evolvable trait (i.e., mutants with different sensory ranges are possible), and if the costs of larger sensory range are not too high, then mutants with larger D may out-compete the resident NE with smaller D.

Response >> We appreciate this thoughtful suggestion. Assessing whether the motor Nash strategies are evolutionarily stable under a fixed detection range—and how this changes when sensory range is allowed to evolve—also seems to require an Adaptive Dynamics analysis (e.g., pairwise invasibility plots) with explicit assumptions about mutation and fitness. We believe that a complete study using Adaptive Dynamics is beyond the scope of the present manuscript. We have added a note in the Discussion to make this limitation explicit and identify this analysis as a priority for future work.

(In the Discussion, Lines 643–647 in the revised manuscript)

“We acknowledge the absence of an ESS analysis as a significant limitation of this study. An Adaptive Dynamics analysis (e.g., via pairwise invasibility plots) would clarify whether the Nash equilibrium defense strategies identified here are evolutionarily stable. This will be a high priority for future work.”

(4)Given that both reviewers have raised concerns about the conceptual distinction between classical and evolutionary game theory, I suggest that the authors look more deeply into this issue and consider addressing it further—perhaps along the lines outlined above.

Response >> We thank the reviewer for highlighting the conceptual distinction between classical and evolutionary game theory. We have revised the Results, Discussion, and Methods to (i) define the scope of our Nash equilibrium analysis as a short-term, stage-game prediction, (ii) delineate its relation to long-term evolutionary stability (ESS), and (iii) state the assumptions under which an Adaptive Dynamics analysis would test whether NE coincides with ESS. We also added a “Limitations” note indicating that Adaptive Dynamics analysis is beyond the present scope and is a priority for future work. We believe these revisions address the concern.

Further minor comments:

(5)Abstract, line 68:

"... and establish their behaviors at Nash equilibria."

→ Not clear. How can agents establish a behavior at Nash equilibria? For me, a behavior can correspond to a Nash equilibrium.

AND

(7)Abstract, line 79:

"...allows agents to compute their payoffs."

→ Unclear. What is meant by this? It’s not evident how this would work in a real-world setting, especially as it seems to require perfect knowledge of the opponent's behavior..

Response >> We appreciate this insightful comment. While the Nash equilibrium is a mathematically defined solution concept, our wording that the agents actively computed it was incorrect; we apologize for these points. To prevent misunderstanding, we have revised the text as follows.

(5) -> (Lines 65–68 in the revised manuscript)

“Under a sensory-motor algorithm without directional information, encounter probabilities were used to construct payoff matrices that admit Nash equilibria; we identified the corresponding equilibrium behaviors for the agents (predators and prey).”

(7)-> (Lines 79–81 in the revised manuscript)

“By implementing these algorithms, agents interact in ways that give rise to payoff matrices from which various Nash equilibrium behaviors can be mathematically derived under different conditions.”

(6)Abstract, line 71:

"...were the non-constant sum and positive sum in most cases."

→ Needs revision. Suggest: "...were, in most cases, non-constant-sum and positive-sum games."

Response >> We thank the reviewers for the helpful suggestion. We have revised the sentence as follows (Lines 71–72 in the revised manuscript).

“The games based on interactions among multiple predators and prey were, in most cases, non-constant-sum and positive-sum games.”

(8)Line 137:

→ Change to: "...increase payoffs by changing their behaviors.".

Response >> We thank the reviewers for the helpful suggestion. We have revised the sentence as follows (Lines 137–140 in the revised manuscript).

“The Nash equilibrium behaviors illustrate how agents engage in specific behavioral patterns and achieve stable payoffs, where neither the predators nor the prey increase their payoffs by changing their behaviors[22,31,33,34]”

(9)Line 145:

The sentence is potentially misleading:

"This study addresses this challenge and reveals a game-theoretic solution for the Nash equilibrium in the interaction between attack and defense."

→ This wording implies that an analytical solution was derived, which is not the case. I recommend: "This study addresses this challenge and identifies Nash equilibria for the interaction between attack and defense using numerical simulations."

Response >> We thank the reviewers for the helpful suggestion. We have revised the sentence as follows (Lines 147–148 in the revised manuscript)

“This study addresses this challenge and identifies Nash equilibria for attack–defense interactions using numerical simulations.”

(10)Line 224:

Some strategies seem biologically implausible, likely due to simplifying assumptions—e.g., that neither predator nor prey can detect relative positions or motion. For instance, for a prey with a greater detection distance than the predator, the "freeze" strategy appears unrealistic. If the prey detects a predator at a distance and has an information advantage, the optimal response would typically be to flee (move directly away from the predator). The freeze strategy may only be advantageous when the predator is already close, has a similar or larger detection range, and can outrun the prey. By freezing the prey could reduce the risk of detection. While I do not see this as a fundamental flaw, I would expect a discussion on how access to directional information could qualitatively alter the resulting Nash equilibria.

Response >> We appreciate the reviewer’s comment regarding the realism of the freeze strategy when prey have a greater detection range than predators. In our single predator–prey model, the long-range freeze equilibrium arose from a single-step interaction assumption: for *D_i_* = 2 we computed encounter probabilities from one forward step and excluded adjacent configurations (Supplementary Program, EncounterProbabilities_gridworld.txt).

When we included adjacent configurations—equivalently, a two-step interaction (Supplementary Program, EncounterProbabilities_gridworld_2.txt)—the prey’s Nash equilibrium switched to escape when the prey’s detection range exceeded the predator’s (Supplementary Fig. 1C, panels f and i). This shows that the earlier freeze at long range was due to the single-step assumption, and that the more realistic escape equilibrium emerges once two-step interactions are allowed, consistent with results from the NetLogo model. We have clarified this in the Results and Methods.

In the Results, we first explained single-step interaction in detail as follows (Lines 202–207 in the revised manuscript).

“*p* was calculated as (number of encounters)/(total number of cases), enumerating all initial predator–prey positions and both agents’ post-first-step headings under detection-informed and uninformed behavior (Supplementary Program, EncounterProbabilities_gridworld.txt). When *D_i_* = 2, agents could detect each other at the outer edge of the detection range, and combinations in which they were adjacent were excluded.”

We then added the explanation as follows (Lines 231–239 in the revised manuscript).

“Because freezing at long range is counterintuitive when prey have a detection-range advantage, we recomputed *p* including adjacent configurations at *D_i_* = 2, which is equivalent to permitting a two-step interaction (Supplementary Program, EncounterProbabilities_gridworld_2.txt). Under this setting, the prey’s Nash equilibrium became escape when the prey’s detection range exceeded the predator’s (Supplementary Fig. 1Cf, i), indicating that the previous freeze was due to the single-step assumption, and that the more realistic escape equilibrium identified once two-step interactions were allowed, consistent with the NetLogo model described in the next section.”

In the Methods section, we explained as follows (Lines 702–727 in the revised manuscript).

“We examined encounters between agents within a single time step. Because the maximum value of *D_i_* was 2, we used a 5 × 5 grid in the x–y plane with integer coordinates, where x and y ranged from 0 to 4, inclusive. A predator was fixed at coordinate (2, 2), and the prey was placed at all possible other coordinates. When *D_i_* = 2, agents could detect each other at the outer edge, and combinations in which they were adjacent were excluded. Encounter probability (*p*) was calculated in R[66] (Supplementary Program, EncounterProbabilities_gridworld.txt) as (number of encounters)/(total number of cases), where “total cases” included all combinations of initial predator–prey positions and both agents’ possible headings after the first step, for both detection-informed and uninformed behaviors. This procedure evaluated a single-step forward transition from all initial configurations where interaction could occur, given (*D_i_*, *S_i_*), rather than multi-step simulations on a finite grid. For each *D_i_*, *p* was plotted against *S_s_* and *S_w_* (Fig. 1B), and the resulting Nash equilibrium behaviors are shown in Fig. 1C.

When *D_s_* = 2, the prey’s Nash equilibrium strategy became freeze ($\bar{S}$*_s_* = −1). It seemed unrealistic for the prey to freeze when the prey had a greater detection distance than the predator (Fig. 1Cf, i; *D_s_* = 2 and *D_w_* = 1 or 0). This arose from a simplifying assumption. We recalculated *p* including combinations in which the agents were adjacent when *D_i_* = 2 (Supplementary Program, EncounterProbabilities_gridworld_2.txt). This program corresponds to calculating encounter probabilities for a two-step interaction. Under these conditions, the prey’s Nash equilibrium strategy became “escape” when it had a greater detection distance than the predator (Supplementary Fig. 1Cf, i). These results indicated that the “freeze” strategy observed at long range arose because only a single-step interaction was considered. When a two-step interaction was considered, the more realistic “escape” strategy was the Nash equilibrium, consistent with results from the NetLogo model.”

(11)Lines 246–249:

On rereading, I remain unclear about the difference between *D_i_* and *d_i_*, and the role of the factor *f*. Is *d_i_* used only in the cost-benefit calculation? Given *D_i_* = *d_i_* * *f* and *f* = 10, with *D_i_* ranging up to 3, *d_i_* must be at most 0.3. This appears a bit arbitrary. Why not equate *D_i_* directly to the trait?

Response >> We appreciate the reviewer’s request to clarify the roles of *D_i_*, *d_i_*, and the factor *f*. In our experiments, *d_i_* denotes a dimensionless sensory-investment trait that enters the cost term, whereas *D_i_* is the implemented detection distance used to compute encounter probabilities. We link them by the scaling *D_i_* = *d_i_* × *f* and, in the experiments discussed here, we cap *D_i_* at 3 to keep the state enumeration tractable; with *f* = 10, this implies *d_i_* ≤ 0.3 for these runs.

We do not equate cost directly to *D_i_* for two main reasons and additional practical reason. First, we require a cost metric that is comparable between the sensory-motor and non-sensory motor algorithm. Charging cost to the internal, dimensionless investment traits *d_i_* (sensory-motor) and *K_i_* (non-sensory motor) allows us to use the same cost function and places both algorithms on a common cost basis, enabling fair comparison; by contrast, using the spatial range *D_i_* would penalize only the sensory-motor formulation. Second, by keeping cost on *d_i_* and relating spatial range via *D_i_* = *d_i_* × *f*, the scale factor *f* can absorb modality- and medium-specific transduction efficiencies (e.g., olfaction, vision, audition, mechanosensation; air vs. water). This design makes the framework readily extensible to future cross-modality comparisons without redefining the cost function or losing comparability.

In practice, using *D_i_* directly in the cost over-penalized large detection radii and made some runs unstable; the *d_i_*-based scaling with *f* avoids this while preserving the trait-to-range mapping. We revised the Methods to state these definitions explicitly and to note that the bound *D_i_* ≤ 3 is a computational convenience for enumeration, not a biological limit on the trait.

In the Results, we explained the first reason using *f* as follows (Lines 264–267 in the revised manuscript).

“We introduced the coefficient *f* to normalize trait values to a common range (0–0.3) between the sensory-motor (*d_i_*) and non-sensory motor algorithms (*K_i_*, where *K_i_* represents the probability of speed change), enabling direct comparison.”

In the Methods, we defined *d_i_*, *D_i_*, and *f*, and explained reasons why we do not equate cost directly to *D*_i_ as follows (Lines 668–677 in the revised manuscript).

“We distinguished between the latent trait *d_i_* (dimensionless sensory investment), which entered the cost function, and its spatial realization *D_i_* (detection distance), which was used to compute encounter probabilities. Information-processing costs were assessed using *d_i_* rather than *D_i_* to avoid conflating environmental units with internal investment and to maintain numerical stability across algorithms. The factor *f* ensured cost comparability across algorithms and helped maintain simulation stability. This scaling allowed us to apply the same cost function to the sensory-motor (*d_i_*) and non-sensory motor (*K_i_*, the probability of speed change) formulations, placing them on a common cost basis and enabling a fair comparison. The bound *D_i_* ≤ 3 was a computational convenience, not a biological limit on the trait.”

(Lines 777–785 in the revised manuscript)

“This design made the framework readily extensible to future cross-modality comparisons without redefining the cost function or losing comparability. By keeping cost on *d_i_* and relating spatial range via *D_i_* = *d_i_* × *f*, the scale factor *f* can absorb modality-specific (e.g., olfaction, vision, audition, mechanosensation) or medium-specific transduction efficiencies (e.g., transparency of air or water). In practice, using *D_i_* directly in the cost over-penalized large detection radii and made some runs unstable; the *d_i_*-based scaling with *f* avoided this while preserving the trait-to-range mapping. The value of *f* was determined from preliminary simulation experiments.”

(12)Figure 2E: The choice of color scale is problematic—predator payoff values occupy only a narrow lower range, making it hard to see any structure in the heatmap. I assume this is to enable direct comparison across panels, but since payoffs are in arbitrary units and predator/prey values likely differ in scale, strict comparability seems unnecessary. Including numerical labels on the color bar would help clarify the magnitude of variation. As it stands, the heatmaps might span only a tiny range (e.g., 1 to 1.001), which is hard to assess.

Response >> We appreciate this helpful comment. Because predator payoffs were concentrated at the lower end, the previous unified color bars made the heatmap structure hard to discern. Accordingly, we (i) rescaled the predator-panel color bars to the predators' data range (Figs. 2E, 4D) and (ii) added numeric labels (minimum and maximum) to the color bars and reported the scale ranges (Figs. 1B, 2C, 2D, 2E, 4B, 4C, 4D, and Supplementary Figs. 1B, 2–5, 10–13). These revisions make it easier to assess the magnitude of variation even when it is small.

(13)Figures (general): The figures appear to be of low resolution, which makes reading small font sizes difficult.

Response >> We apologize for the low resolution of the figures in the PDF. We have updated the PDF to use the same high-resolution figures used in the manuscript submission, improving legibility.

##########

Reviewer 2

Major comments:

(1)The authors do not provide a resolution for the point that not all Nash equilibria (NE) are evolutionarily stable strategies. The paragraphs added to the discussion are welcome in that they add context to the results, but they do not resolve the underlying issue: To reach a Nash equilibrium either via learning or evolution, the predator and prey require knowledge of all payoffs (encounter probabilities) which they cannot have. And to make statements about evolutionary outcomes, we still do not know if the NE described are indeed evolutionarily stable.

The authors say this on lines 528–530 but, in my opinion, it is not clear enough to the reader that this is quite a major impediment to the biological utility of this study. Ideally, I’d like the authors to find the evolutionarily stable strategies for the considered games. I am not saying that the nash equilibrium is completely uninformative, but without knowledge of what the ESS is, it is hard to say if these results truly represent what happens over the course of evolution.

Response >> We appreciate this important critique. We have now made two points explicit. First, in our analysis, agents were not assumed to know or compute the full payoff matrix; the payoff matrices and the Nash equilibria were constructed and identified by the analyst as a short-term, within-interaction benchmark. Any earlier wording suggesting that agents “establish” or “compute” an NE has been revised. Second, Nash equilibria need not be evolutionarily stable, and without an evolutionary analysis we cannot claim that the reported NEs are ESS. We clearly separated short-term NE predictions from long-term evolutionary stability, and we stated that this constitutes a substantive limitation of the present study’s biological generality. We outlined the required test via Adaptive Dynamics, by introducing a rare mutant into a homogeneous, well-mixed resident population playing the NE strategy, evaluating whether any sufficiently similar rare mutant would fail to increase in frequency (based on encounter rates, payoffs, information-processing costs, and reproduction), and concluding ESS only if all such mutants do not spread. We have revised the Discussion to include these assumptions and criteria and to state plainly that an NE may or may not coincide with an ESS, and that determining ESS for the considered games is beyond the scope of the present manuscript but is a priority for future work.

(In the Discussion, Lines 597–628 in the revised manuscript)

“We now turn to the evolutionary implications of attack and defense behaviors, which are directly subject to selection through predation[53]. We used Nash equilibria as short-term reference points for the game; agents were not presumed to know or compute the full payoff matrix. In real predator–prey settings, predators can learn from experience (successful and failed attacks) and thereby estimate local expected payoffs using sensory-motor algorithms implemented by neural circuits, without access to all payoffs. Such learning can adjust behavior toward the short-term equilibrium predictions under stationary conditions, yielding flexibility on short time scales. We suggest that predators with neuronal mechanisms for learning, rather than directly executing innate attack behaviors, may be favored by natural selection[54–59].

In contrast, individual prey cannot reliably learn from fatal defensive failures; hence, defense is best analyzed within an evolutionary framework. The evolutionary status of these behaviors depends on whether the resident strategy resists invasion by rare mutants. In evolutionary game theory, an evolutionarily stable strategy (ESS) is a resident strategy that cannot be invaded by any sufficiently similar rare mutant under fitness-proportionate reproduction[40,60]. Because our Nash equilibrium results were derived from single interactions, this analysis did not test rare-mutant invasion; therefore, a Nash equilibrium may or may not coincide with an ESS. A formal assessment would require an Adaptive Dynamics analysis[61] by assuming a homogeneous, well-mixed, statistically stationary resident prey population playing the Nash equilibrium strategy, introducing a rare mutant, and evaluating whether it increases or declines when introduced at very low frequency. If every sufficiently similar rare mutant fails to increase, the Nash equilibrium is an ESS; otherwise, it is not.

Although it remains unknown whether the Nash equilibrium defense strategies identified here are evolutionarily stable, the corresponding behaviors—such as escaping and freezing—are often innate or developmentally specified. In mice, for example, escaping and freezing can be elicited by genetically hard-wired circuits with minimal learning[4,6–9,11,12,14,15]. The asymmetry—predators can adjust their attack behavior through experience, whereas prey cannot learn from fatal defense failures—suggests that the mechanisms differ: predators rely on strategy learning, whereas prey defenses are largely innate and genetically specified, implying distinct evolutionary bases for attack and defense[62].”

(In the Discussion, Lines 643–647 in the revised manuscript)

“We acknowledge the absence of an ESS analysis as a significant limitation of this study. An Adaptive Dynamics analysis (e.g., via pairwise invasibility plots) would clarify whether the Nash equilibrium defense strategies identified here are evolutionarily stable. This will be a high priority for future work.”

(2)The authors, in response to a previous comment of mine, have added a sentence “Consequently… structurally favoring prey” on line 564–566. It is a (really) good observation, but I’m not sure it explains everything. I get now that the asymmetry arises from the fact that it is easier for the prey to not-die than for the predator to eat. But this effect also exists under the sensory-motor algorithm, doesn’t it? So there is still some reason that the non-sensory motor algorithm exacerbates this already present asymmetry.

Response >> We appreciate this point. The spatial asymmetry that favors prey is present under both algorithms, but it is expressed more strongly at the Nash equilibrium under the sensory-motor algorithm. With detection, both agents can choose when to start moving and which direction to take. In particular, once the prey detects the predator, it can choose among several headings that increase separation, whereas the predator’s options are largely limited to moving toward the target. Consequently, the equilibrium encounter probability p is lower than under the non-sensory motor condition, and the prey’s payoff 1− *p* is higher. By contrast, the predator’s gain from directed pursuit is offset by the prey’s separation-increasing moves, so predators show no payoff advantage (Fig. 5C,E vs. 5B,D). Under the non-sensory motor algorithm, both agents wander and meet at a random encounter rate; because the prey cannot detect the predator in advance and avert encounters, it cannot fully exploit directional choice. We have revised the text to make this mechanism explicit.

In the Discussion, we explained it as follows (Lines 581–592 in the revised manuscript).

“This pattern reflects a structural asymmetry inherent in the IBM. Under the sensory-motor algorithm, both predator and prey adjust their movement timing and heading based on sensory information. The prey gains a marked advantage because any heading other than toward the predator promotes escape, whereas the predator’s viable headings are mostly constrained to moving toward the target. Therefore, at the Nash equilibrium, the prey’s encounter probability, *p_s_*, is reduced, resulting in a higher prey payoff, 1 − *p_s_*, with no corresponding increase in the predator’s payoff (Fig. 5C,E vs. 5B,D). By contrast, under the non-sensory motor algorithm, the two agents move without cues, so encounters are governed by chance. The prey cannot detect the predator in advance and avert encounters, and thus cannot fully exploit the wider range of available headings. Therefore, the sensory-motor algorithm was more beneficial to prey than the non-sensory motor algorithm.”

(3)I would say that the other main biological outcome of this work is that (i) the Nash equilibrium can be pure or mixed, and increased *D_i_* seems to systematically promote mixed strategies. Given that it is mentioned in both the abstract and author summary, the authors also seem to see this as a main result. Yet, the authors do not offer an explanation for why this happens. I appreciate that the authors made Additional Fig. 2 to alleviate my concern, but the only main text sentence that tries to offer insight into this is in Lines 505–508, but this is also quite vague – what are these “multiple agent effects”, and how exactly are they attributable to “alterations in encounter frequencies and agent numbers”?

Response >> We now clarify why larger *D_i_* promotes mixed Nash equilibria under the sensory-motor algorithm. Increasing *D_i_* gives both agents earlier detection and more directional turning before encounter, thereby increasing the influence of behavior whether attack or defense succeeds.

As a result, the payoff landscape becomes structurally more complex—for example, with greater curvature and additional local extrema—and a pure-strategy balance becomes harder to sustain. The indifference condition means that a player’s expected payoffs against the opponent’s two behaviors are equal; as *D_i_* increases, the set of (*S_s_*, *S_w_*) pairs that satisfy this condition expands, and the best-response curves intersect at probabilities strictly between 0 and 1 (i.e., in the interior of the probability space). In this situation, no pure behavior is a mutual best response, so each player must randomize between the two behaviors, yielding a mixed Nash equilibrium. This is the standard characterization of a mixed Nash equilibrium via the indifference principle: each player chooses mixing probabilities so that every behavior included in the mix yields the same expected payoff against the opponent’s mixture (Osborne & Rubinstein, A Course in Game Theory, 1994, ch. 3, Fig. 35.1). For these reasons, we interpret the more frequent behavioral switching (mixing) as *D_i_* increases.

Consistent with this mechanism, the overall payoff range was not substantially wider at larger *D_i_* (Supplementary Fig. 16), yet the landscape exhibited greater structural complexity (Fig. 1Bc, 2D–E; Supplementary Figs. 1Bc, 2–4). This yielded more mixed equilibria and more frequent switching (Figs. 1Cc, 2Fg; Supplementary Figs. 2–4, 9).

In the Results, we first described it as follows (Lines 436–439 in the revised manuscript).

“Finally, we examined the conditions promoting switching. Increased *D_i_* tended to systematically promote mixed strategies (Figs. 1Cc, 2Fg, and Supplementary Figs. 2–4, 9). Moreover, Nash equilibrium behavior switching frequently occurred at small values of *c_b_* (Fig. 3Dg).”

Then, in the Discussion, we explained it as follows (Lines 534–553 in the revised manuscript).

“In the sensory-motor algorithm, when detection distance was long (large *D_i_*) or behavioral costs were small (*c_b_* = 0.001), we observed frequent behavioral switching (Figs. 1Cc, 2Fg, 3Dg; Supplementary Figs. 2–5, 9). Mechanistically, large *D_i_* provided both agents with earlier detection and more available headings before encounter, and the small *c_b_* reduced the weight of the cost term. Both factors increased the influence of behavior on whether attack or defense succeeds and increased the structural complexity of the payoff landscape. The indifference condition means that a player’s expected payoffs from its two behaviors are equal (given the opponent’s mixture). Greater structural complexity increases the number of *S_i_* pairs that satisfy this condition. When both players satisfy the indifference condition, the best-response curves—the relationship between the opponent’s behavior and the player’s payoff-maximizing behavior probability—meet at probabilities strictly between 0 and 1 for both players. In that case, no pure behavior is a mutual best response, so both players must randomize between the two behaviors, yielding a mixed Nash equilibrium[47]. Consistent with this mechanism, the payoff landscapes exhibited greater structural complexity (additional bends and local extrema; Fig. 1Bc, 2D–E; Supplementary Figs. 1Bc, 2–5). Together, these yielded more mixed equilibria and more frequent switching (Figs. 1Cc, 2Fg; Supplementary Figs. 2–5, 9). Importantly, the overall payoff space, that is, the spread of (*P_s_*, *P_w_*) pairs, was not substantially wider even in conditions where behavioral switching occurs (Supplementary Fig. 16).”

Response >> By “multiple-agent effects” we refer to density-dependent, population-mediated feedbacks. These indirect, population-mediated feedbacks are absent in the single-pair model (Supplementary Fig. 8), which is why the patterns we report are specific to the multi-agent setting.

In the Discussion, we explained it as follows (Lines 561–565 in the revised manuscript).

“The observed changes in agent payoffs—both agents' payoffs increase through attacks without defense, and the prey payoff decreases through behavioral switching—do not appear in a single predator–prey setting (Supplementary Fig. 8), indicating that they arise from density-dependent, population-mediated feedbacks specific to the multi-agent setting (i.e., multiple-agent effects).”

(4)The payoffs (*P_w_*, *Ps*) calculated from the NetLogo simulations are a function of what happens when the whole prey population is interacting with the whole predator population, and not what happens in an interaction between two agents on their own. The latter is what one usually puts into a payoff matrix. The authors say (line 284) that they treat the population as a pool of potential players engaging in pairwise contests. This is good, and I think it is defensible to define payoff here as “equilibrium probability of finding a prey per timestep”, but it is certainly out of the ordinary. So I think this should be said to give readers the correct context, and also emphasize the novelty of this approach.

Response >> Thank you for highlighting this point. We agree that our payoff construction is non-standard relative to the usual practice of building a payoff matrix from a single isolated pairwise interaction. To make the context and novelty explicit, we have revised the Results, Discussion, and Methods to state that: (i) although the NetLogo IBM contains many agents, we treat the whole population as a pool of potential players engaging in pairwise contests; (ii) after the transient in the simulation had settled, under a homogeneous, well-mixed, statistically stationary population, we used the population-level per-step encounter probability *p_i_* to define per-agent, normalized payoffs for the two-player game (*P_w_* = *p_w_* for predators and *P_s_* = 1 − *p_s_* for prey); and (iii) this “pool of players” representation aggregates simultaneous multi-agent interactions and thereby captures density-dependent (population-mediated) effects that single-pair payoffs cannot. We also note that this is a modeling choice that departs from the conventional construction but is appropriate for our purpose of linking IBM outcomes to a two-player game form. The new wording appears in Results, Discussion, and Methods of the revised manuscript.

In the Results (Lines 308–316 in the revised manuscript)

“Although the IBM included many agents, we treated the entire population as a pool of potential players engaging in pairwise contests in a two-player game[45], rather than assuming that every individual was a player. Under a homogeneous, well-mixed, statistically stationary population, we used the population-level per-step encounter probability to define per-agent normalized payoffs *P_i_*. Unlike the standard construction that builds a payoff matrix from an isolated single pairwise interaction, this pool of players approach aggregated simultaneous multi-agent interactions, thereby capturing density-dependent (population-mediated) effects in predator–prey games that the single-pair method did not reveal.”

In the Discussion (Lines 513–519 in the revised manuscript)

“We adopted a pool of players representation: the many-agent IBM was treated as a population from which pairwise contests were drawn, and per-agent payoffs were defined from the stationary population-level encounter probability[45]. This approach differs from the conventional single-pair construction by design. It clearly mapped IBM outcomes to a two-player game and also revealed density-dependent effects that arose from simultaneous interactions.”

In the Methods (Lines 847–853 in the revised manuscript)

“The NetLogo IBM included many agents. For game-theoretic analysis, we treated the population as a pool of potential players engaging in pairwise contests[45]. We defined normalized per-agent payoffs as *P_w_* and *P_s_*. This differed from the conventional method of constructing payoff matrices from single isolated pairwise interactions and was intended to aggregate simultaneous multi-agent interactions, thus capturing density-dependent, population-mediated effects that were absent in single-pair settings.”

(5)To reiterate, I do think the approach of empirical game theoretic analysis can be useful, and is a novel way (at least in theoretical ecology and evolution) of dealing with complex scenarios. My objections only come from the fact that the biological utility of these results depends strongly on how one chooses to analyse the empirical game.

Response >> Thank you for this thoughtful comment. We agree that empirical game-theoretic analysis is a useful and novel way to study complex systems, and that the biological value of the results depends on how the empirical game is analyzed. In the revision, we make our analysis choices explicit and explain why we made them. We also see this approach as helpful for neuroscience research. Mapping naturalistic behavior in complex environments onto game-theoretic payoffs and equilibria helps reveal the underlying algorithms and computations. We hope this work helps bridge neuroscience and theoretical ecology and evolution.

Further minor comments:

(6) In abstract

-Line 58. change to “is a central question in …”

-L71 remove the first “the”, sentence should read “…were non-constant sum and…”

Response >> We appreciate the corrections. We changed Line 58 in the revised manuscript to “is a central question in …” and Line 71 “… were, in most cases, non-constant-sum and …”.

(7)Line 722 “predators gain energy as a behavioral outcome in proportion to their encounter probability”: this choice in the model is confusing. why aren’t predators just given energy immediately when they meet and eat a prey?.

Response >> Thank you for pointing out the ambiguity. In the IBM, energy was updated event-by-event: predators gained energy only upon actually consuming a prey individual during an encounter (event gain = wolf-gain-from-food), whereas prey gained energy from grazing in steps without an encounter (event gain = sheep-gain-from-food). After each simulation run, we estimated the per-step encounter probability *p_i_* under the homogeneous, well-mixed assumption. We defined payoffs *P_i_* directly from the encounter probability *p_i_*: *P_w_* = *p_w_* and *P_s_* = 1 − *p_s_*, at each time step. The event-gain constants (wolf-gain-from-food, sheep-gain-from-food) were used only for event-driven energy bookkeeping in the IBM. For the payoff-matrix analysis, we used *P_i_* that was averaged over time steps 10,000–11,000; these averages represented post-transient, statistically stationary values. We have revised the Methods accordingly.

In the Methods, we explained it as follows (Lines 816–830 in the revised manuscript).

“In the IBM, energy was updated event-by-event: predators gained the constant wolf-gain-from-food only when they consumed a prey individual during an encounter, whereas prey gained sheep-gain-from-food from grazing in steps without a predator encounter. These constants were used solely for event-driven energy accounting in the IBM; their values were calibrated in preliminary simulations and are listed in Supplementary Table 1. For the payoff-matrix analysis used to identify Nash equilibria, we defined normalized payoffs. Accordingly, the payoff of wolves (*P_w_*) was defined as the probability of encountering prey (*P_w_* = *p_w_*), and the payoff of sheep (*P_s_*) as the probability of evading predators (*P_s_* = 1 − *p_s_*). *P_s_*, *P_w_*, and differences in payoffs (*P_diff_* = *P_s_* − *P_w_*) were calculated at each time step. Under the assumption of uniform interactions in a well-mixed environment, we approximated per-agent payoffs by the population-level payoffs based on the encounter probabilities. These values fluctuated periodically with periods shorter than 1,000 time steps. We averaged *E*, *P_i_*, and *P_diff_* over time steps 10,000–11,000; these averages represented post-transient, statistically stationary values.”

(8)In abstract -L75 “a simple non-sensory motor algorithm was sufficient to lead to their Nash equilibria.” this is misleading because a Nash equilibrium always exists (also see below). I would suggest rephrasing to something like “Nash equilibrium behaviors were also computed under a simpler non-sensory motor algorithm.”

and

Line 162 “Moreover,…equilibria”: This kind of sentence is misleading as I have already pointed out. In any game with a finite number of strategies, (mixed-strategy) Nash equilibria always exist, so it is not surprising – if a payoff matrix is constructed, a (mixed) Nash eq. can be found. The authors also present such statements on e.g. Lines 158, 409, 545. I would suggest changing these and any others I might not have found. On L409 specifically: “To investigate whether sensory input is necessary for agents to establish Nash equilibria,…” is especially misleading. Sensory input is unnecessary because an NE exists of course. But it is not unnecessary because the factor being considered is sensory input, it is unnecessary because an NE always exists..

Response >> We thank the reviewer for pointing this out. In finite games, the existence of a (mixed-strategy) Nash equilibrium is guaranteed by Nash’s theorem; our wording unintentionally suggested that agents “establish” or “compute” the equilibrium or that sensory input is required for its existence. We have revised the text to avoid this ambiguity. Throughout, we now state that the payoff matrices admit NE and that we identify the NE under each information condition, focusing on how sensory input changes the equilibrium composition and payoffs, rather than on existence.

In the Abstract, we revised as follows (Lines 65–70 in the revised manuscript).

“Under a sensory-motor algorithm without directional information, encounter probabilities were used to construct payoff matrices that admit Nash equilibria; we identified the corresponding equilibrium behaviors for the agents (predators and prey). Different detection distances yielded distinct Nash equilibrium behaviors, representing a computational mechanism that can account for diverse attack and defense behaviors.”

(Lines 76–77 in the revised manuscript)

“Moreover, Nash equilibrium behaviors were also identified under a simpler non-sensory motor algorithm.”

In the Author Summary (Lines 90–92 in the revised manuscript).

“Using individual-based models, we simulated and analyzed predator–prey interactions and computed the Nash equilibria of the resulting payoff matrices; at these equilibria, no agent can increase its payoff by a unilateral deviation.”

In the Introduction (Lines 147–148 in the revised manuscript).

“This study addresses this challenge and identifies Nash equilibria for attack–defense interactions using numerical simulations.”

(Lines 160–165 in the revised manuscript).

“Based on this framework, we computed Nash equilibria in attack and defense behaviors between the predator and prey[37]. A variety of Nash equilibrium behaviors were observed depending on the sensory abilities, including chasing and ambushing in predators, escaping and freezing in prey, and behavioral switching in both agents. Moreover, Nash equilibria were also identified in a simple algorithm without sensory input (non-sensory motor algorithm).”

In the Results, (Lines 443–444 in the revised manuscript).

“To investigate how sensory input influences equilibrium strategies and payoffs, we tested a simple non-sensory motor algorithm.”

In the Discussion (Lines 566–567 in the revised manuscript).

“Furthermore, we identified Nash equilibria under a non-sensory motor algorithm driven solely by movement variability.”

(9)Single step encounter probabilities: the outcome depends strongly on the starting position of the sheep (which determines whether or not it detects the wolf at 2,2), and in which direction they both turn after the first step (whether they detected something or not). Was the encounter probability averaged over different possible pairs of initial positions and turning possibilities?

Response >> Yes. As the reviewer notes, we computed the encounter probability as (number of encounters)/(total number of cases), where “total cases” included all initial predator–prey position pairs and both agents’ post-first-step headings, with and without detection (for *D_i_* = 2, adjacent configurations were excluded because only a single forward step was considered). We have clarified this in the Results and Methods.

In the Results, we explained single-step interaction in detail as follows (Lines 202–207 in the revised manuscript).

“*p* was calculated as (number of encounters)/(total number of cases), enumerating all initial predator–prey positions and both agents’ post-first-step headings under detection-informed and uninformed behavior (Supplementary Program, EncounterProbabilities_gridworld.txt). When *D_i_* = 2, agents could detect each other at the outer edge of the detection range, and combinations in which they were adjacent were excluded.”

In the Methods, we explained it as follows (Lines 708–715 in the revised manuscript).

“Encounter probability (*p*) was calculated in R[66] (Supplementary Program, EncounterProbabilities_gridworld.txt) as (number of encounters)/(total number of cases), where “total cases” included all combinations of initial predator–prey positions and both agents’ possible headings after the first step, for both detection-informed and uninformed behaviors. This procedure evaluated a single-step forward transition from all initial configurations where interaction could occur, given (*D_i_*, *S_i_*), rather than multi-step simulations on a finite grid. For each *D_i_*, *p* was plotted against *S_s_* and *S_w_* (Fig. 1B), and the resulting Nash equilibrium behaviors are shown in Fig. 1C.”

(10)Figures. there are no values on any colourbars, e.g. in Figure 1B, 2C,D,E what are the minimum and maximum encounter probabilities & payoffs?

Response >> We agree that the absence of numeric labels on the color bars obscured the absolute scales of encounter probabilities and payoffs. We have added numeric labels (minimum and maximum) to the color bars and reported the scale ranges (Figs. 1B, 2C–E, 4B–D, and Supplementary Figs. 1B, 2–5, 10–13).

(11)Line 110: typo, did you mean to say “differences in interaction probability”?

Response >> We thank the reviewer for catching this error. We have corrected it as follows: (In the Introduction, Lines 111–113 in the revised manuscript)

“It has been argued that predator and prey behaviors influence the spatial arrangement between them, resulting in differences in interaction probability—here defined as the encounter probability—and directly impacting predation[16].”

Ref.

Osborne & Rubinstein, A Course in Game Theory, 1994
https://sites.math.rutgers.edu/~zeilberg/EM20/OsborneRubinsteinMasterpiece.pdf
