# Supplementary material for: Nash equilibrium of attack and defense behaviors between predators and prey
Source: PLoS Comput Biol. 2025 Nov 21;21(11):e1013730. doi: 10.1371/journal.pcbi.1013730 (PMC12671891; doi:10.1371/journal.pcbi.1013730)
Supplement: Attachment — Submitted filename: R3_Response to Reviewers.docx [file pcbi.1013730.s046.docx]

2025-11-08

Dear Editor and Reviewers,

We would like to express our sincere gratitude to both reviewers for their careful and thorough reading of the manuscript from their expert perspective. We apologize for the weaknesses in the previous manuscripts and are deeply grateful for the opportunity you have patiently afforded us to improve them.

In this *Response to Reviewers*, we have addressed each point raised, provided clarifications, and incorporated the reviewers’ suggestions. Point-by-point responses are presented below. Reviewer comments are shown in blue, with our responses following in black, and revised sentences in red.

In the marked-up copy of our manuscript (*R3_**Manuscript with Track Changes.docx*), newly added or changed text is shown in red.

We sincerely appreciate the opportunity to revise and resubmit our work, and we thank the editors and reviewers for their continued, thoughtful guidance during this third round of review.

Hiroyuki Ichijo

##########

Reviewer 1

On the usage of “admit a Nash equilibrium” and similar: I am sorry to be annoying and point this out yet again, but I am afraid I have not communicated my point well enough. So to prevent even longer discussion on this topic, I will try to be a bit more direct.

Response >> We sincerely apologize for not fully understanding the Reviewer 1’s point earlier. We have carefully revised the manuscript following your valuable advice.

on L67 and L568: it is still misleading to say that “This algorithm was sufficient to modify agents’ payoffs and yielded payoff matrices that admitted Nash equilibria” (L568). This is because all algorithms would generate some payoff matrix, and all payoff matrices admit a Nash equilibrium! Therefore, no information can be gained by saying that the algorithm being considered leads to a payoff matrix that admits a NE. Wording of the form “we identify NE..” that theauthors use elsewhere is appropriate and resolves my concerns completely. The useful information in this study is to know what exactly the NE is when the simulations are analysed game-theoretically, and NOT that it exists. I suggest the authors remove the phrase “that admit Nash equilibria” on L67 and remove the sentence on L568 altogether.

Response >> Following the reviewer’s advice, we removed the phrase “that admit Nash equilibria” from the revised manuscript, as shown below.

(Line 65 [Red numbers indicate the line number in *R3_Manuscript.docx*])

“Using encounter probabilities to construct payoff matrices under a sensory–motor algorithm that lacked directional information, we identified the corresponding equilibrium behaviors for the agents (predators and prey).”

Response >> We removed the sentence “This algorithm was sufficient to modify agents’ payoffs and yielded payoff matrices that admitted Nash equilibria”, which appeared in the previous version (R2), and revised it as shown below.

(Line 567)

"Furthermore, we identified Nash equilibria under a non-sensory motor algorithm driven solely by movement variability (Fig. 4)."

(Line 576: “Given that movement variability alone can generate equilibria..”. anything will generate equilibria! rephrase. Perhaps the authors wish to differentiate between pure- and mixed-strategy equilibria in the above sentences. However I don’t think this is the case because pure- and mixed-strategy NE exist under both the sensory-motor and non-sensory-motor algorithms.

Response >> As Reviewer 1 correctly pointed out, the finite discrete strategy space and continuous payoff functions constructed in this study’s IBM satisfy the conditions of Nash’s theorem; therefore, the existence of a Nash equilibrium is automatically guaranteed. We acknowledge that the original text did not accurately reflect this point, and we have removed the corresponding phrase, “Given that movement variability alone can generate equilibria,” from the manuscript.

(Line 575)

“We further examined how the presence of sensory input influences outcomes.”

Line 629 “This study demonstrated the mathematical existence of Nash equilibria in predator–prey interactions”. This is technically true, but Nash’s result (PNAS 1950, ref below) already guaranteed the mathematical existence of this study's Nash equilibria. Another fact that is perhaps useful to reiterate: the paper of Nash (1950) applies also to non-constant-sum games since the proof only uses continuity of the payoff functions, which is satisfied here. To borrow author phrasing, this study _identifies_ what these Nash equilibria should be in predator-prey interactions.

Response >> As you correctly pointed out, the mathematical existence of Nash equilibria is already guaranteed by Nash’s theorem; therefore, we deleted the corresponding part and revised the text as follows.

(Line 627)

“A theorem by Nash (1950) guarantees the mathematical existence of Nash equilibria in any finite game[32]. This study identified the specific Nash equilibrium strategies underlying attack–defense behaviors in predator–prey interactions and clarified their behavioral significance and evolutionary implications.”

A typo that the authors might want to change: “Porf.” on Line 936

Response >> We sincerely apologize for the oversight. We are embarrassed by our careless mistake, and it has been corrected.

(Line 937)

“Prof. Rahul Savani”

We have reread the entire manuscript and checked for any other potentially misleading statements, careless mistakes, or grammatical errors.

Among them, in the beginning of the Discussion section (L506 in *R2_**Manuscript.docx*), we deleted the phrase “showed the mathematical existence of” and revised it as follows.

(Line 506)

“We developed a game-theoretic framework to analyze predator–prey interactions and identified Nash equilibria in payoff matrices derived from encounter probabilities in both the sensory–motor and non-sensory motor algorithms.”

We also found other minor mistakes. We have indicated the previously overlooked errors in red in *R3_Manuscript with Track Changes.docx*, and we have corrected them in *R3_Manuscript.docx*.

##########

Reviewer 2

The authors addressed my points in a satisfactory way.

Response >> We sincerely appreciate Reviewer 2’s constructive comments and valuable critiques on the previous versions of our manuscript.
